# Risk factors for postherpetic neuralgia: a meta-analysis based on demographic, clinical features, and treatment characteristics

**DOI:** 10.3389/fimmu.2025.1667364

**Published:** 2025-10-01

**Authors:** Jing Wang, Rong Tao, Yinghai Jiang, Zhuoya Ma, Lingjie Xia

**Affiliations:** Pain Medicine Department, Henan Provincial People’s Hospital (Zhengzhou University Affiliated People’s Hospital), Zhengzhou, China

**Keywords:** PHN, meta, analysis, risk, factor

## Abstract

**Background:**

This study aims to comprehensively analyze the independent risk factors of postherpetic neuralgia (PHN) through a systematic evaluation, including demographic characteristics, clinical manifestations, treatment regimens, comorbidities, and virological factors, in order to provide evidence-based support for the early identification of high-risk patients and the optimization of preventive strategies in clinical practice.

**Method:**

A systematic search of PubMed, Embase, and the Cochrane Library was conducted to identify studies reporting risk factors for PHN. After screening the literature according to predefined inclusion and exclusion criteria, effect size indicators such as odds ratios (OR) and 95% confidence intervals (95% CI) for each risk factor were extracted. Meta-analyses were performed using RevMan 5.4 and Stata 15.0 software, with a random-effects model applied to pool effect sizes. Publication bias was assessed using Egger’s test, and sensitivity analysis was conducted by sequentially removing individual studies to verify the robustness of the result.

**Results:**

Age (≥60 years), severe rash manifestations, prodromal pain symptoms, smoking history, alcohol abuse, immunosuppressive status, and comorbidities including diabetes mellitus, chronic obstructive pulmonary disease (COPD), hypertension, malignant tumors, or chronic kidney disease, along with high viral load, have been identified as independent risk factors for the development of PHN(p<0.05). In contrast, gender differences and socioeconomic status were not significantly associated with PHN incidence, with insufficient evidence observed (I²>50%, p>0.05).

**Conclusion:**

This meta-analysis confirms that older age (≥60 years), severe rash, prodromal pain, immunosuppressive therapy, and smoking are significant risk factors for PHN. Furthermore, it identifies alcohol abuse, T2DM, COPD, hypertension, cancer, high pain scores (as measured by VAS or NRS), and high HZ viral load as additional risk factors. COVID-19 may represent a potential risk factor that must be further investigated. The association between socioeconomic status and PHN remains inconclusive, while antibody levels against varicella-zoster virus (VZV) may serve as a protective factor.

**Systematic Review Registration:**

https://www.crd.york.ac.uk/PROSPERO/, identifier CRD42025629699.

## 1 Introduction

Herpes zoster (HZ) is a self-limiting dermatological disease caused by the varicella-zoster virus (VZV), with millions of new cases reported worldwide each year (1). Postherpetic neuralgia (PHN) is one of the most common complications following reactivation of the virus, often leading to severe neuropathic pain that requires pharmacological treatment (2). PHN, with an incidence ranging from 5% to 30% (3), represents a major global public health burden. Although various treatment options, such as pharmacological therapy and nerve blocks are available for PHN, some individuals exhibit poor pain tolerance during episodes, leading to suboptimal therapeutic outcomes. Therefore, investigating and understanding the risk factors associated with PHN should be a major focus of clinical research. However, the profile of potential risk factors for PHN has not yet to been comprehensively defined.

Several meta-analyses have investigated the risk factors for PHN, but each has several limitations. The meta-analysis by Forbes et al. (4) was published early and did not employ a standardized quality assessment tool. The study by Haiou Zhou et al. included a limited number of studies and lacked registration in a meta-analysis protocol database (5). The meta-analysis by Ding did not perform a complete sensitivity analysis and omitted evaluation of certain population-specific risk factors (3). In order to address these limitations and enhance the current evidence base regarding PHN risk factors, our study adopts the Newcastle-Ottawa Scale (NOS) for quality assessment, utilizes PubMed, Cochrane, and Embase as literature sources, and incorporates sensitivity analyses. The findings generated through this comprehensive approach are expected to offer a more robust and detailed overview of PHN risk factors, thereby supporting the development of preventive strategies.

## 2 Method

PROSPERO registration ID: CRD42025629699.

### 2.1 Study selection

A comprehensive literature search was conducted in PubMed, Embase, and the Cochrane Library for studies published from January 2001 to December 2024, using keywords related to PHN and risk factors. Detailed search strategies are provided in the **Supplementary Material**.

### 2.2 Inclusion and exclusion

This review included observational studies and meta-analyses examining risk factors for PHN. The studies were conducted in community-based, primary care, or hospital settings and involved either general populations or specific subgroups, such as older or individuals with comorbidities. The study population consisted of adults aged ≥18 years with a confirmed diagnosis of HZ, and PHN was defined as pain persisting ≥90 days after rash onset (The 90-day endpoint is standard because a 30-day period cannot reliably distinguish acute pain from established PHN, while a 120-day period introduces unacceptably high dropout rates and prolongs trial duration unnecessarily). To ensure comprehensiveness, studies from all countries and regions were considered. Studies were excluded if they focused on populations aged <18 years, involved immunocompromised individuals (e.g., those with HIV) (4) without separate analyses for the general population, were conducted exclusively in laboratory or non–patient-based settings, or did not apply standardized definitions of PHN or consistent outcome measures.

### 2.3 Data extraction

**Figure 1:**
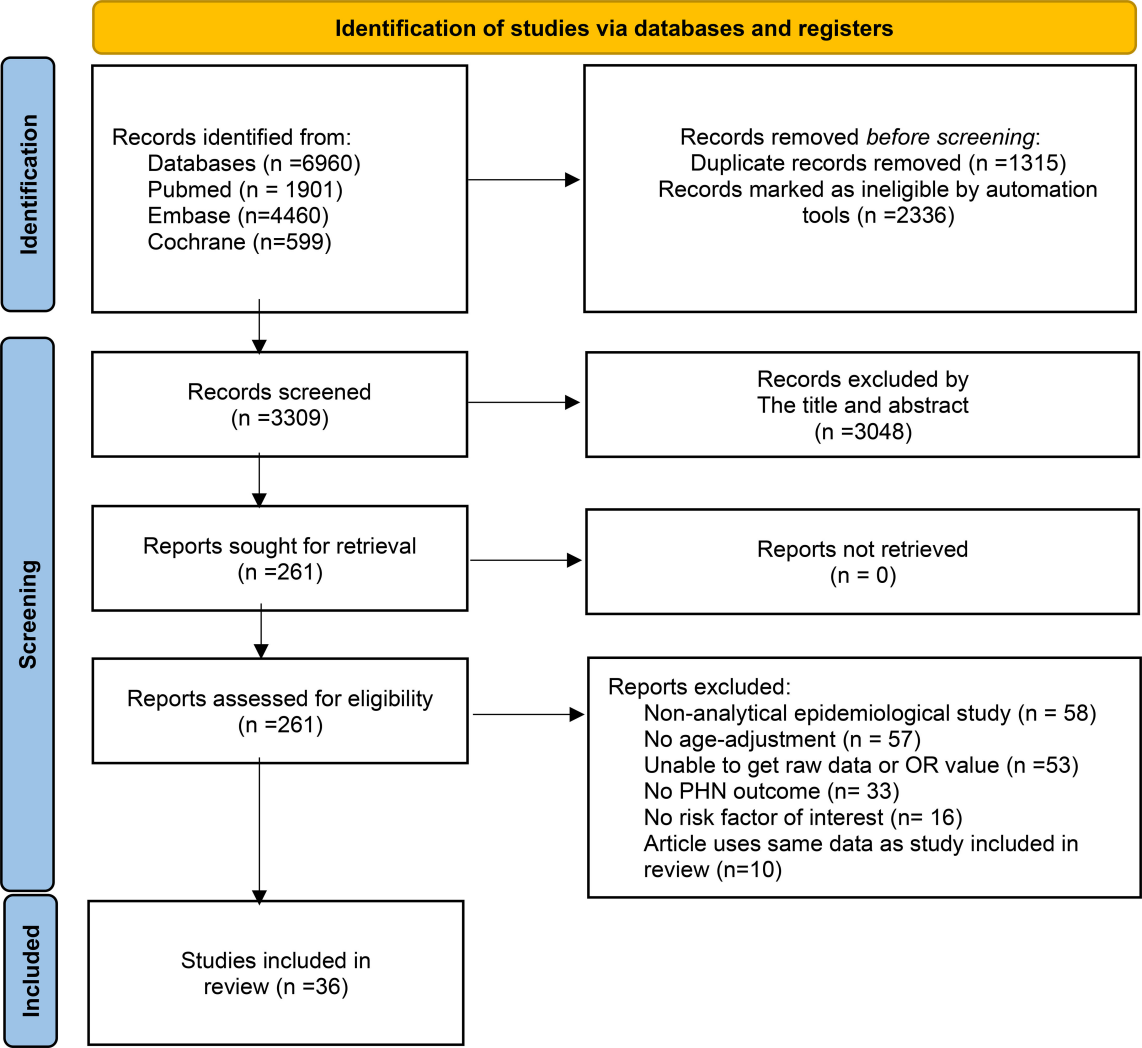
PRISMA 2020 flow diagram for new systematic reviews which included searches and assessment process.

After removing duplicate records using the EndNote, two independent reviewers screened the titles and abstracts identified through the systematic search to assess eligibility. Studies that were clearly irrelevant or failed to meet the inclusion criteria were excluded at this stage. Full-text articles of potentially relevant studies were retrieved and independently assessed, with any disagreements resolved through discussion or consultation with a third reviewer. The entire study selection process was documented using a PRISMA flow diagram, including reasons for exclusion at the full-text review stage (**Figure 1A**). Data were extracted on participants’ age, sex, comorbidities, and baseline characteristics; types of risk factors and their corresponding odds ratios; and the incidence, severity, and duration of PHN. A standardized data extraction form was developed by the research team, and for studies with missing or unclear data, corresponding authors were contacted to obtain additional information.

### 2.4 Risk of bias (quality) assessment

In this study, the NOS was used to assess the quality of original observational studies and randomized controlled trials, while the AMSTAR-2 tool was applied to evaluate the methodological quality of included meta-analyses. Two trained reviewers independently conducted the risk of bias assessment using the respective scoring systems, and cross-checked the results. Disagreements were resolved through discussion or consultation with a third reviewer. According to the NOS, studies were categorized as low quality (0–3 points), moderate quality (4–6 points), or high quality (7–9 points). For AMSTAR-2, a review was considered high quality if it met all items or had no more than one non-critical weakness, moderate quality if it had no critical weaknesses but up to two non-critical ones, and **low quality** if it had one critical item missing. The results were provided in the **Supplementary Information 1**.

### 2.5 Data analysis

All statistical analyses were performed using STATA software (version 15.0). Effect sizes were expressed as odds ratios (ORs), hazard ratios (HRs), or risk ratios (RRs), along with their corresponding 95% confidence intervals (CIs). Heterogeneity across studies was assessed using the I² statistic and Cochran’s Q test, with thresholds defined as follows: low heterogeneity (I² < 30%), moderate heterogeneity (I² = 30–60%), and high heterogeneity (I² > 60%). A random-effects model was employed for meta-analyses.

To explore potential sources of variability, subgroup analyses were conducted based on different categories of PHN risk factors. Sensitivity analyses were performed by excluding studies with a high risk of bias to evaluate the robustness of the results. Funnel plots were generated to assess publication bias. When more than 10 studies were included, meta-regression analyses were conducted to investigate potential sources of heterogeneity.

## 3 Results

### 3.1 Results of literature screening

The initial search identified 6,960 publications. After excluding literature that did not meet the inclusion criteria, removing duplicates, and considering the timeliness of the research, studies published between 1950 and 1999 were also excluded. Finally, a total of 36 articles were included in the analysis. The literature screening process is illustrated in **Figure 1**. The characteristics of these studies are showed in **Supplementary Table 1**.

### 3.2 Risk factors for PHN based on demographics

#### 3.2.1 Age

**Figure 2:**
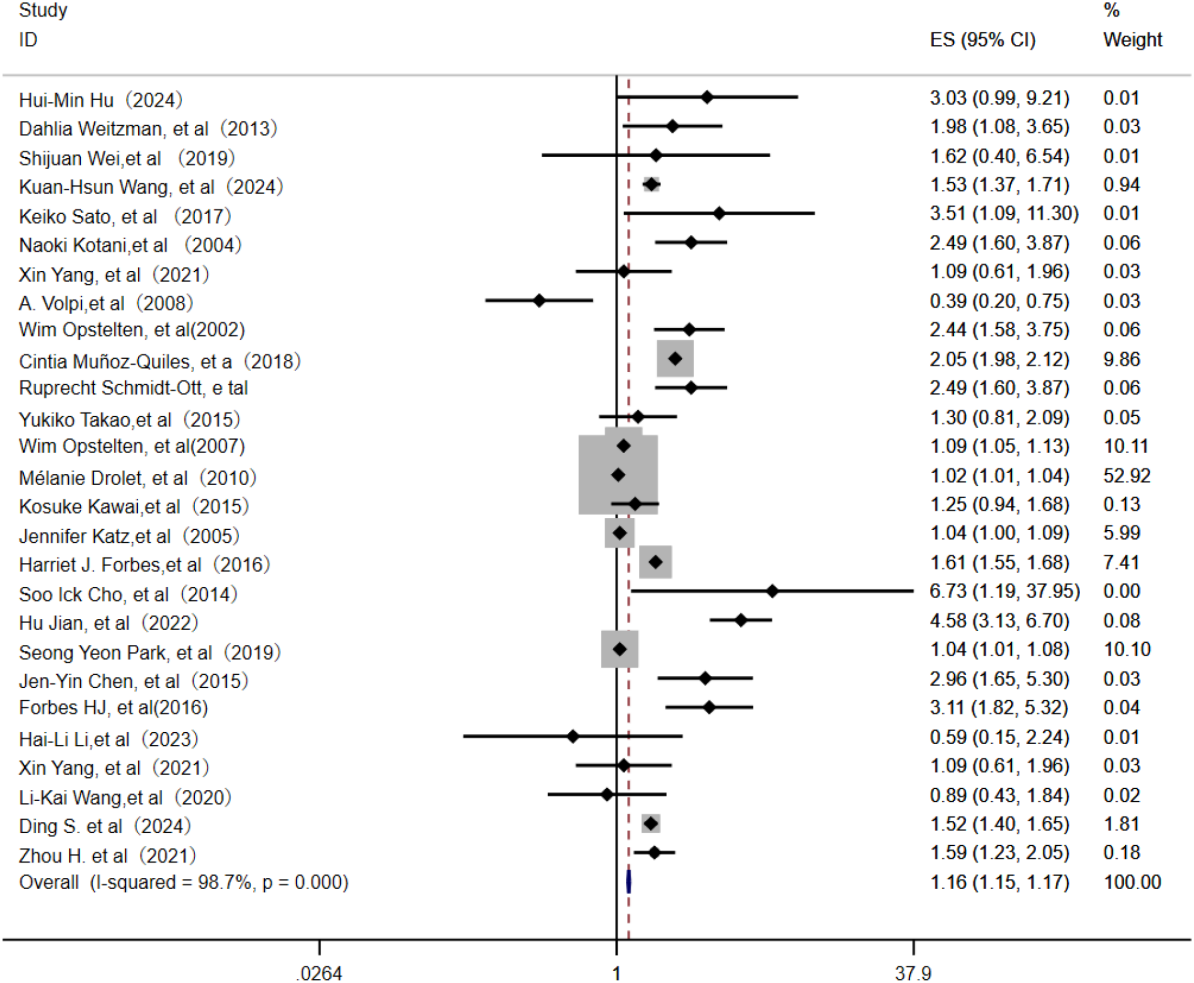
Meta-analysis of age as a risk factor of PHN. PHN, postherpetic neuralgia.

Among 36 identified studies, 27 evaluated age as an independent risk factor for PHN (3–23). In this meta-analysis of 24 studies, the random-effects model (**Figure 2**) indicated that age was an independent risk factor for PHN (OR = 1.16; 95% CI: 1.15–1.17). Subgroup analysis (**Supplementary Figure 1**) indicated age stratification (particularly “per-10-year increase” and ≥60 years) was likely source of heterogeneity, with these subgroups showing the greatest heterogeneity. Assessment of publication bias revealed some asymmetry in the funnel plot (**Supplementary Figure 2**), but Egger’s test indicated no significant publication bias (P > 0.05, **Supplementary Information**). Sensitivity analysis confirmed result stability, with only the Mélanie Drolet study affecting the OR when excluded (**Supplementary Information**).

#### 3.2.2 Gender

**Figure 3:**
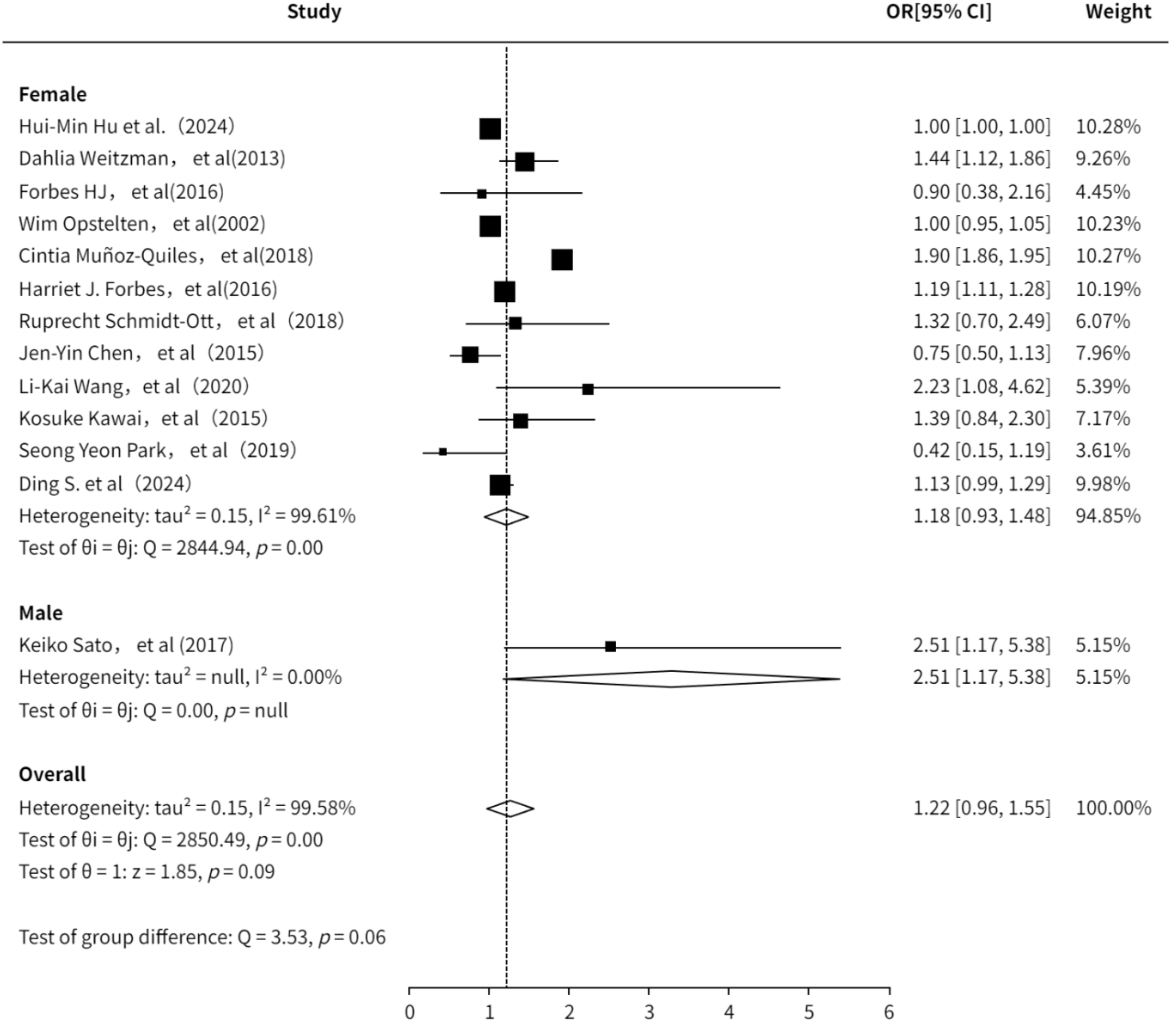
Meta-analysis of gender as a risk factor of PHN.

A total of 11 studies (3, 6, 7, 10, 13–16, 18, 20, 21, 23, 24) evaluated the association between gender and PHN. The pooled analysis indicated a positive association between gender and PHN (OR = 1.22, 95%CI: 0.96-1.55; **Supplementary Figure 3**). Subgroup analysis revealed a lower risk estimate in females compared to males, although this finding was based on only one study focusing on male participants and should be interpreted with caution. The subgroup analysis result (**Figure 3**) is consistent with previous studies suggesting that female is a risk factor for PHN (OR = 1.18, 95% CI 0.93–1.48). The sensitivity analysis also demonstrated the robustness of the meta-analysis results (**Supplementary Information**). Both funnel plot (**Supplementary Figure 4**) and Egger’s test consistently demonstrated no significant publication bias (**Supplementary Information**).

#### 3.2.3 Social economic

**Figure 4:**
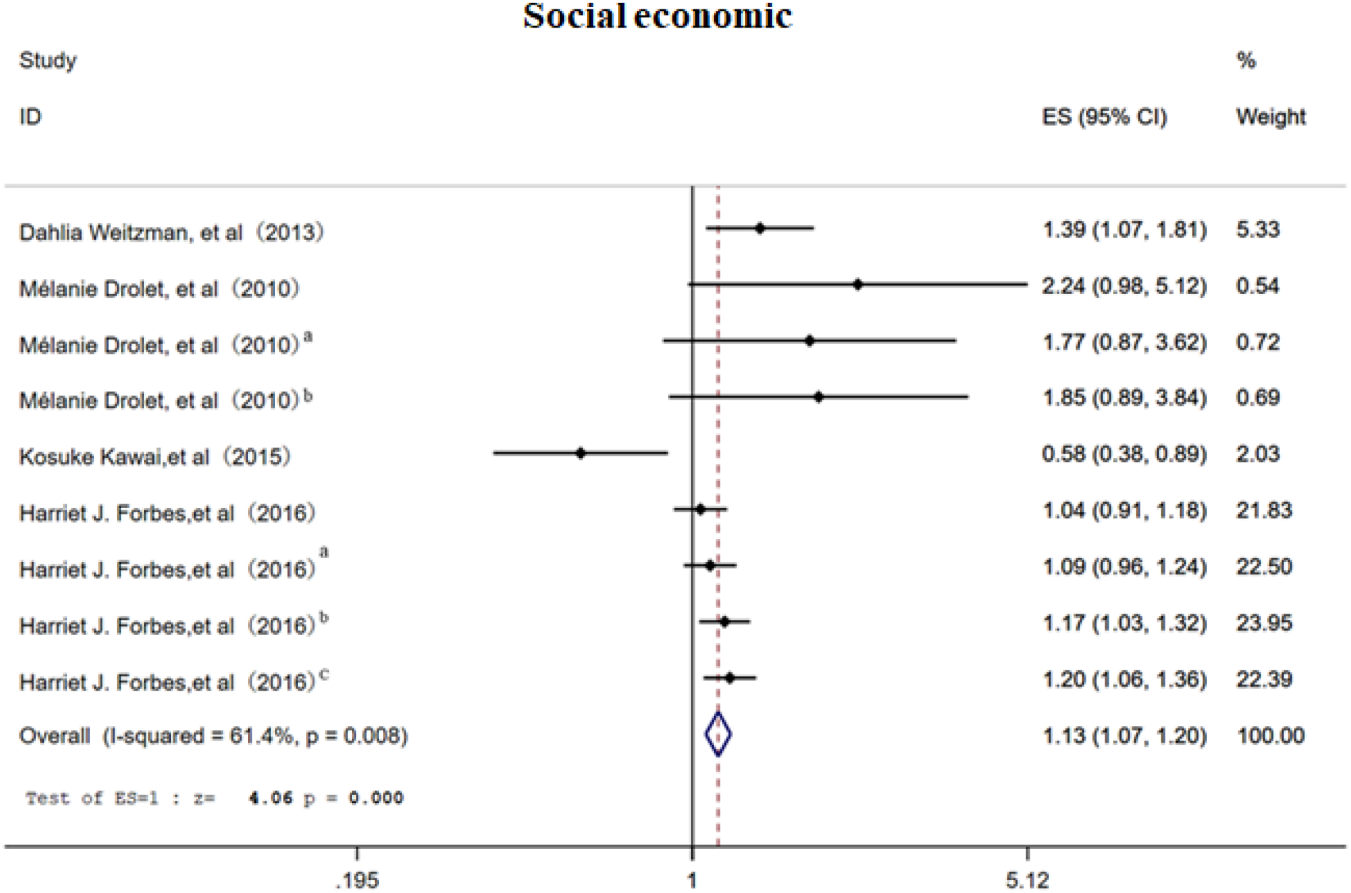
Meta-analysis of gender as a risk factor of PHN. Mélanie Drolet, a, income (20000-39999/year); b, income (<20000/year). Harriet J. Forbes, a, Socioeconomic status 3; b, Socioeconomic status 4; c, Socioeconomic status -most deprived. The higher the serial number, the lower the income.

This meta-analysis included 4 studies (7, 16, 18, 25) covering 9 distinct categories of socioeconomic status, including income level, employment status, and socioeconomic ranking, to assess the association between socioeconomic factors and the risk of PHN. The random-effects model (**Figure 4**) revealed that economic status was associated with PHN (OR = 1.13, 95% CI 1.07–1.20), with moderate heterogeneity across studies (I² = 61%). Subgroup analysis (**Supplementary Figure 5**) suggested that differences in the indicators used to assess socioeconomic status might be the primary source of heterogeneity. Notably, the limited sample sizes of the included studies may have reduced the precision and stability of the effect estimates. Furthermore, inconsistencies were observed among studies: Drolet et al. found a positive association between economic status and PHN, while Forbes et al. found a negative association. These conflicting results suggest that the association between economic status and PHN may be affected by unobserved factors, such as healthcare accessibility and differences in health behaviors. Sensitivity analysis demonstrated that the overall effect estimate remained stable when each study was excluded sequentially, indicating good robustness of the findings. Both funnel plot (**Supplementary Figure 6**) and Egger’s test (p>0.05, **Supplementary Information**) consistently demonstrated no significant publication bias.

#### 3.2.4 Life history

**Figure 5:**
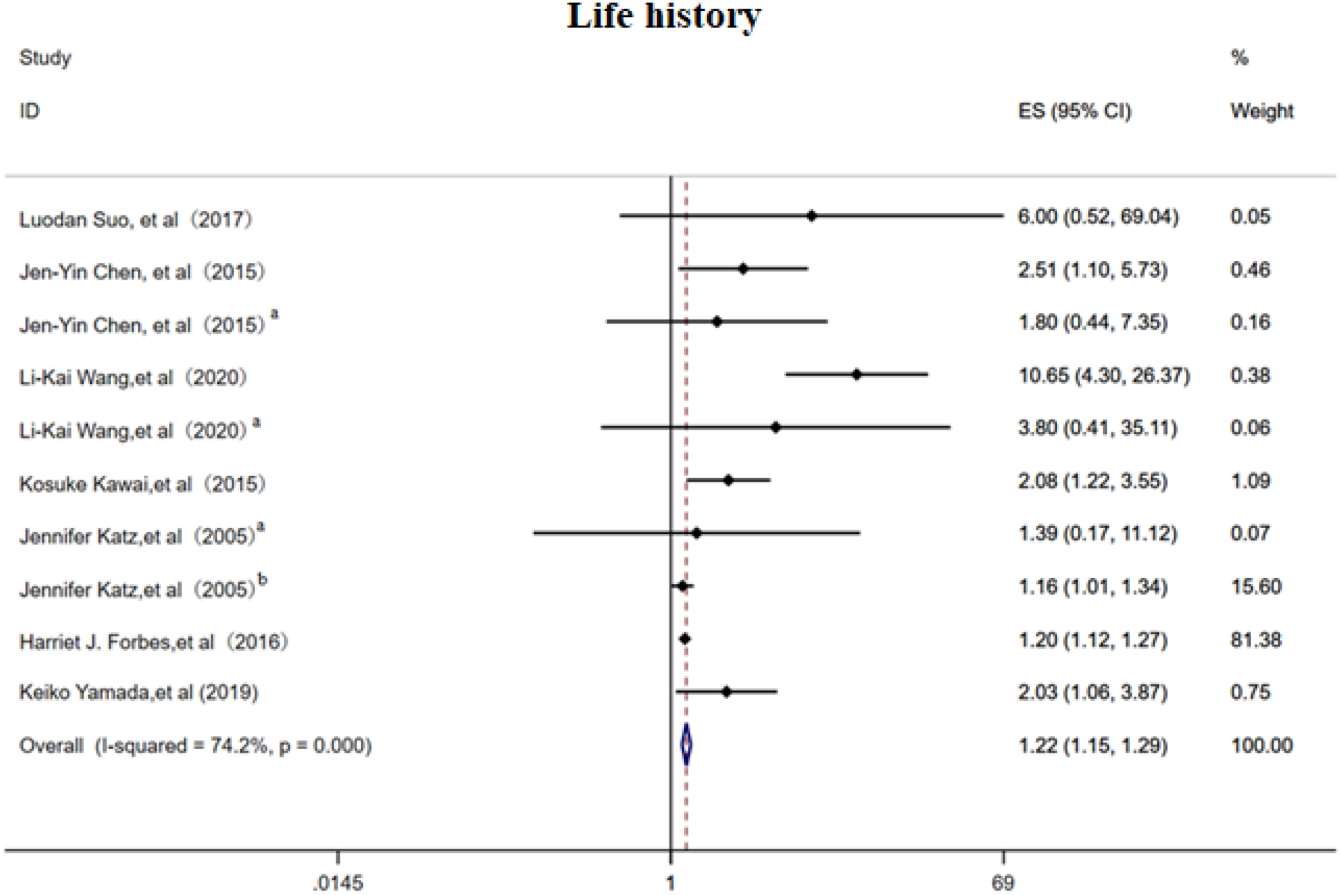
Meta-analysis of life history as a risk factor of PHN. Jen-Yin Chen, Smoking, Jen-Yin Chen ^a^ Alcoholism. Li-Kai Wang, Smoking; Li-Kai Wang^a^, Alcoholism. Jennifer Katz^a^, Immune status; Jennifer Katz^b^, Physical health.

The random-effects model analysis demonstrated that personal life history (16–18, 21, 23, 26, 27) serves as a significant risk factor for PHN (OR = 1.13, 95% CI: 1.07-1.20, **Figure 5**), with moderate-to-high heterogeneity observed across studies (I² = 74.20%). Subgroup analyses (**Supplementary Figure 7**) revealed that smoking history constituted the most substantial source of heterogeneity among personal life history factors, while maintaining a significant association with PHN risk (OR = 1.22, 95% CI: 1.15-1.30). The subgroup results should be interpreted with caution due to the limited number of studies. Sensitivity analysis confirmed the robustness of the results (**Supplementary Information**). Publication bias assessment indicated inconsistent findings: visual inspection of the funnel plot suggested potential positive bias (**Supplementary Figure 8**), while Egger’s test did not reach statistical significance (p>0.05, **Supplementary Information**), indicating no definitive evidence of publication bias. These results emphasize the need for further high-quality research to better clarify these associations.

### 3.3 Risk factors for PHN based on clinical features

#### 3.3.1 Clinical symptoms

**Figure 6:**
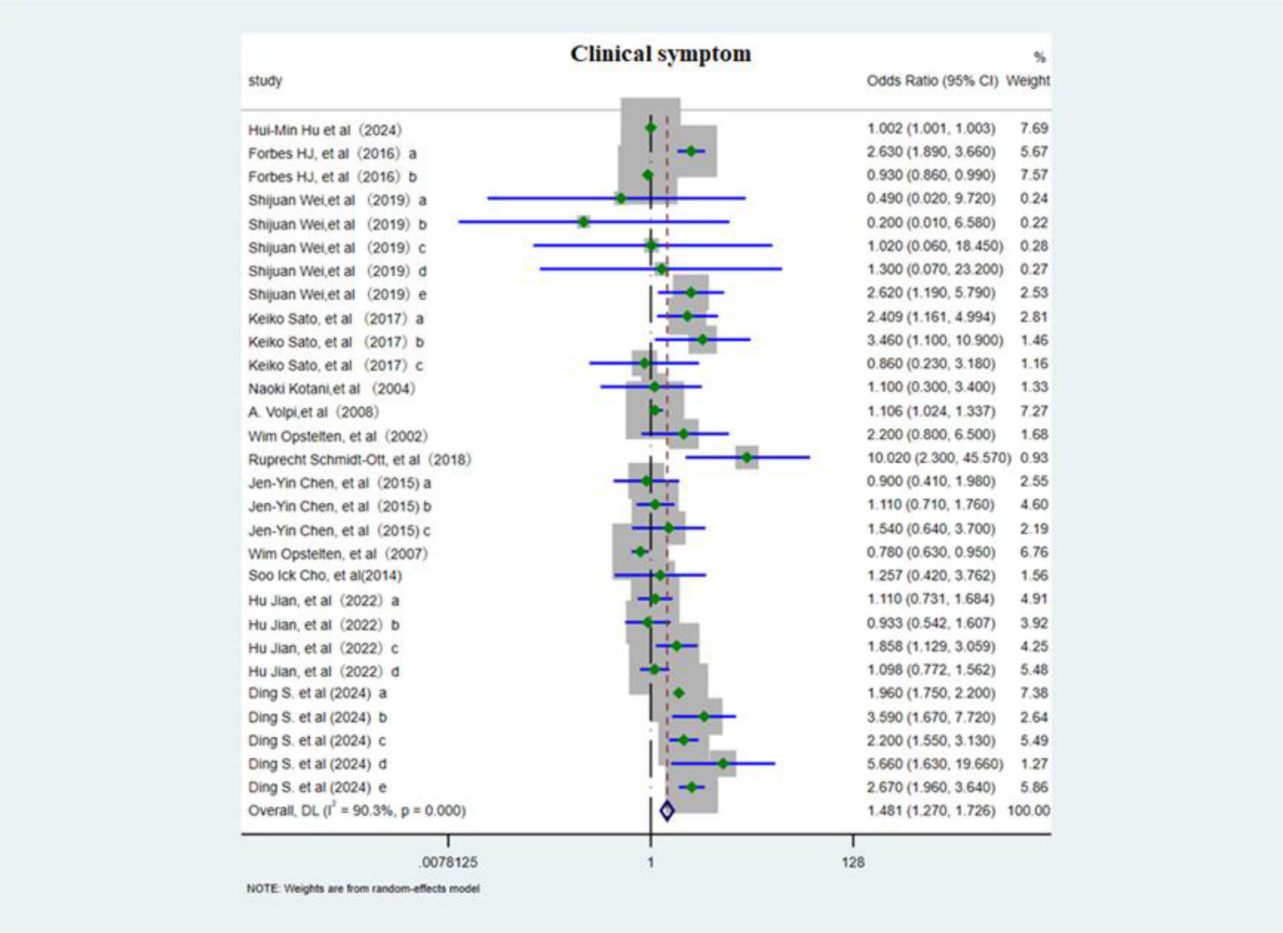
Meta-analysis of clinical symptom as a risk factor of PHN. Forbes HJ, a, Severe rash; b, Rash duration at presentation; Shijuan Wei, a, Brachial plexus; b, Cervical plexus; c, Thoracic nerve; d, Lumbosacral nerve. e, Left. Keiko Sato, a, moderate/severe HZ; b, Upper arms (rash); c, the area of rash ≥3cm^3^. Jen-Yin Chen, The rash location. a, Cervical; b, Thoracic; c, Lumbar and sacral. Hu Jian, The rash location. a, Facial; b, Shoulder and neck; c, Upper arms; d, lower legs. Ding S, The rash location and severe. a, Ophthalmic; b, thoracic nerve; c, nontrunk; d, skin lesion (greater than 5% of the body surface area); e, moderate/severe rash.

This meta-analysis included 13 studies (3, 6, 10, 12–14, 18, 19, 21, 28, 29) evaluating 29 distinct risk factors and employed a random effects model to assess the association between various clinical symptoms and PHN. The OR was 1.48 (95% CI 1.27–1.73; **Figure 6**), showing a positive association, although considerable heterogeneity was present. In subgroup analysis (**Supplementary Figure 9**), greater rash extent and severity conferred a higher risk of PHN (OR 1.53, 95% CI 1.25–1.88), whereas nerve involvement may potentially serve as a protective factor against PHN (OR 0.93, 95% CI 0.87–0.997). An acute episode of HZ markedly elevated risk of PHN (OR 4.21, 95% CI 1.08–16.45). Sensitivity analysis (**Supplementary Information**) confirmed that the pooled estimate remained stable when any single study was excluded, and Egger’s test (p<0.05, **Supplementary Information**) and funnel plot (**Supplementary Figure 10**) asymmetry consistently indicated publication bias.

#### 3.3.2 Pain

**Figure 7:**
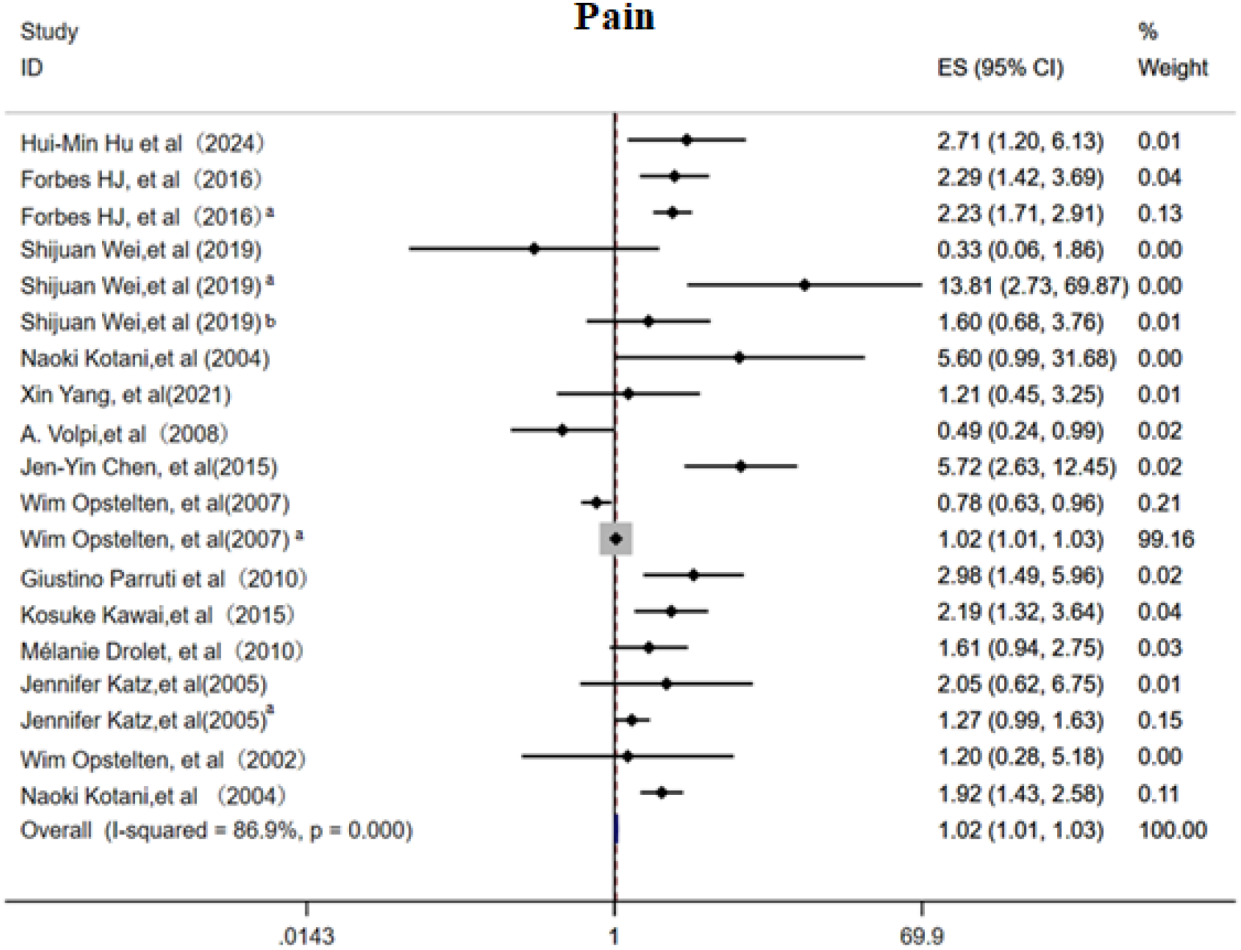
Meta-analysis of pain as a risk factor of PHN. Prodromal phase and severe, Shijuan Wei, Pain after rash; Shijuan Wei^a^, moderate/severe pain; Shijuan Wei^b^, Rash and pain occur simultaneously. Wim Opstelten, Rash and pain occur simultaneously; Wim Opstelten^a^, Severity acute pain (per VAS unit). Jennifer Katz, Prodromal phase; Jennifer Katz ^a^, Acute phrase pain.

This meta-analysis included 14 cohort studies (6, 11–13, 15–18, 21, 25, 28, 30) to systematically evaluate the association between acute phase and post rash pain characteristics and the risk of PHN. The overall random effects model demonstrated a significant positive correlation between acute phase pain intensity and PHN risk (OR 1.02, 95%CI 1.01-1.03, **Figure 7**), with high heterogeneity observed across studies. Subgroup analysis (**Supplementary Figure 11**) revealed that patients with moderate to severe pain had the highest PHN risk (OR 2.50, 95% CI 1.80–3.46), and persistent post rash pain also significantly increased PHN risk (OR 2.05, 95% CI 1.59-2.65). Prodromal pain showed a positive but non-significant overall trend, with neither a duration >3 days nor the simultaneous occurrence with rash proving to be significant predictors of PHN. Sensitivity analyses (**Supplementary Information**) revealed that while the overall results were robust, the exclusion of studies examining “pain severity per one-unit increase in VAS score” significantly altered the pooled effect estimates. Egger’s test (**Supplementary Information**) and the funnel plot (**Supplementary Figure 12**) suggested slightly publication bias(p<0.05).

#### 3.3.3 Clinical index

**Figure 8:**
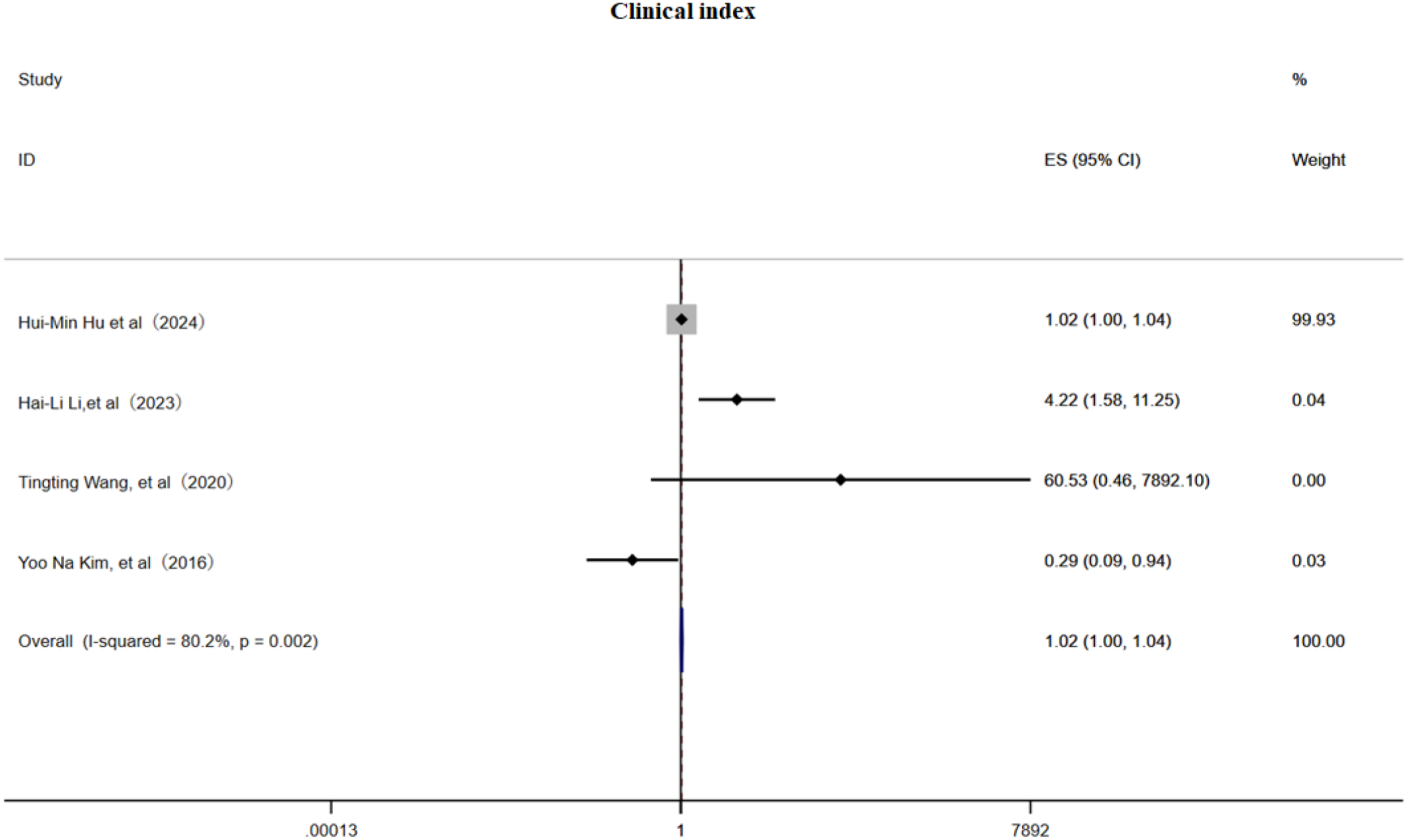
Meta-analysis of clinical index as a risk factor of PHN.

This meta-analysis included 4 cohort studies (6, 22, 31, 32) and employed a random effects model to examine the association between clinical pain assessment scores and the risk of PHN. The pooled estimate indicated a slightly but significant positive relationship (OR 1.02, 95% CI 1.00–1.04, **Figure 8**), although substantial heterogeneity was present (I² = 80.2%). Subgroup analysis (**Supplementary Figure 13**) demonstrated that a VAS score > 3 was associated with PHN (OR = 1.02, 95% CI 1.00–1.04), an NRS-11 score > 3 (OR = 4.22, 95% CI 1.58–11.25), and an NRS-11 score (OR = 60.53, 95% CI 0.46–7 892.10);while one study reporting NRS-11 scores may be a protective factor (OR = 0.29, 95% CI 0.09–0.94), indicating that scale selection and cutoff values markedly influence effect estimates. Sensitivity analysis (**Supplementary Information**) showed that omitting any single study had only a minimal impact on the OR, confirming the robustness of the results; Egger’s test (**Supplementary Information**) and a funnel plot (**Supplementary Figure 14**) provided no evidence of publication bias. In summary, clinical pain scores exhibit a weak to moderate positive correlation with PHN risk, underscoring the need for standardized assessment tools in future investigations.

#### 3.3.4 Others

**Figure 9:**
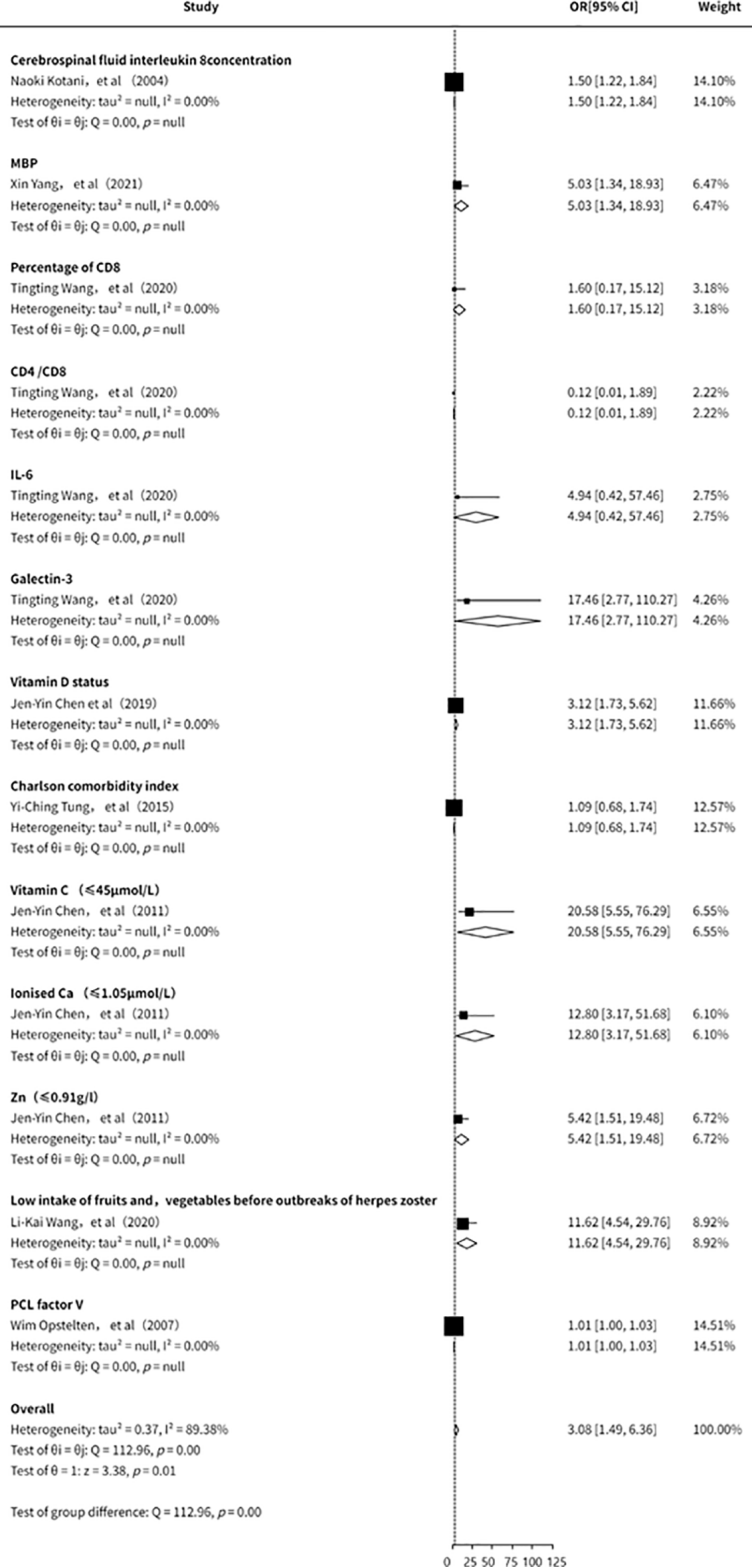
Meta-analysis of other clinical features as a risk factor of PHN.

This study also conducted an analysis of other clinical laboratory indicators in relation to the risk of PHN (11, 15, 21, 23, 28, 31, 33). However, due to the limited number of included studies, the overall findings were inconclusive. Existing evidence (**Figure 9**) suggests that IL-8, myelin basic protein (MBP), CD8+ cell proportion, IL-6, Galectin-3, vitamin D, vitamin C, and zinc may be associated with an increased risk of PHN. Nevertheless, the small number of studies, insufficient strength of evidence, and potential publication bias indicate that these findings should be interpreted with caution. Further high-quality studies are warranted to validate these associations.

### 3.3 Therapy

**Figure 10:**
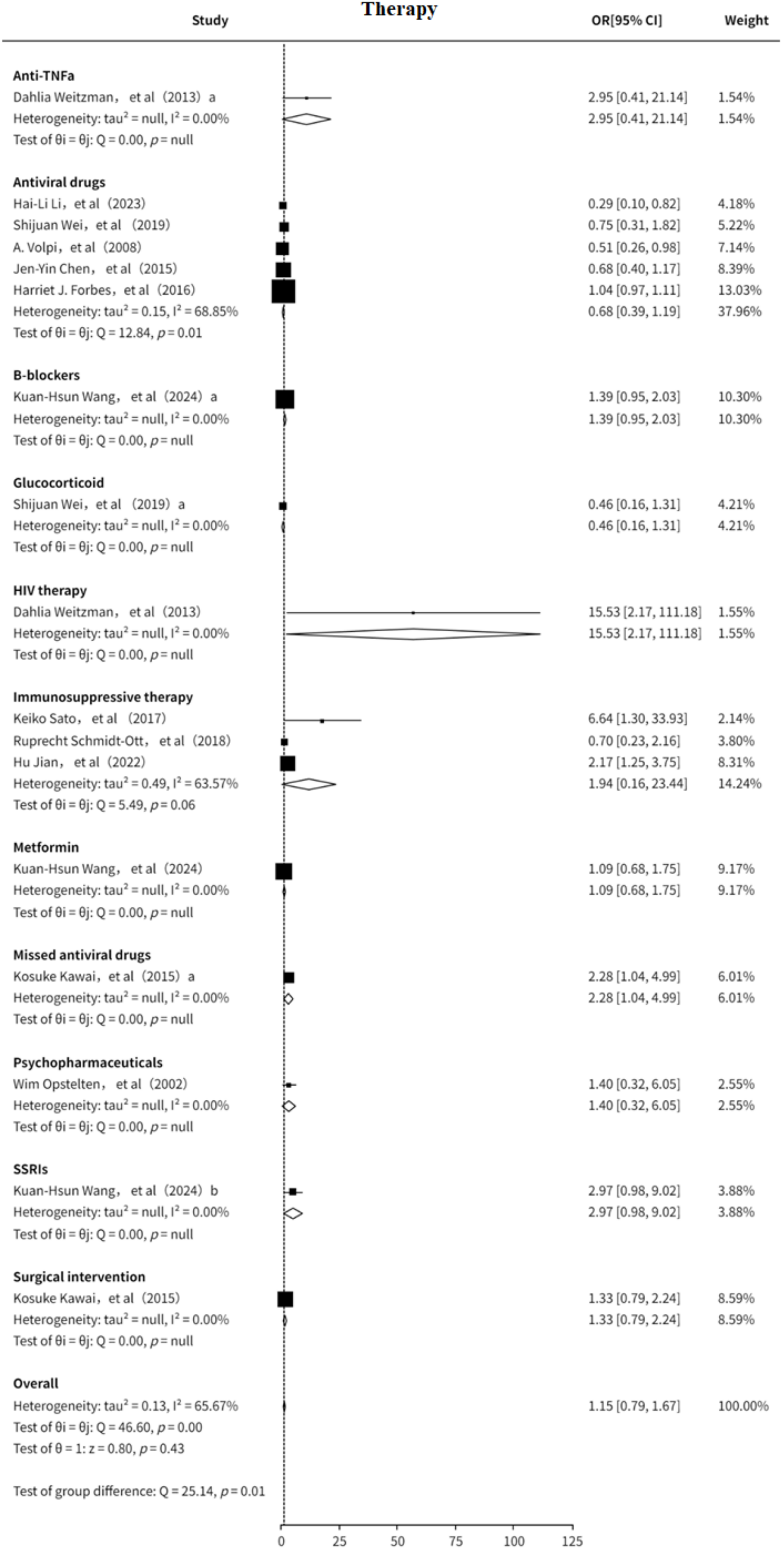
Meta-analysis of therapy as a risk factor of PHN.

This study also analyzed the association between treatment strategies and the risk of PHN (7–10, 12–14, 16, 18, 19, 21, 22). The results suggested that the included treatment modalities may be slightly positively associated with PHN risk (OR = 1.15, 95% CI: 0.89-1.49), although significant heterogeneity was observed (I² = 65.67%, **Supplementary Figure 15**). Subgroup analysis (**Figure 10**) indicated that antiviral therapy may serve as a protective factor against PHN (OR = 0.68, 95% CI: 0.39–1.19). In contrast, immunosuppressive therapy appeared to be a potential risk factor for PHN (OR = 1.94, 95% CI: 0.16–23.44). Other treatment strategies were evaluated in only a limited number of studies, and thus could only be preliminarily suggested as potential risk factors. Funnel plot analysis revealed no significant publication bias, and sensitivity analysis confirmed the stability of the results. The sensitivity analysis (**Supplementary Information**) confirmed the robustness of the results. Both Egger’s test (**Supplementary Information**) and funnel plot (**Supplementary Figure 16**) analysis showed no evidence of publication bias (p > 0.05) Overall, immunosuppressive therapy may increase the risk of PHN, while antiviral treatment may offer a degree of protective benefit findings that warrant further clinical investigation.

### 3.4 Virus

**Figure 11:**
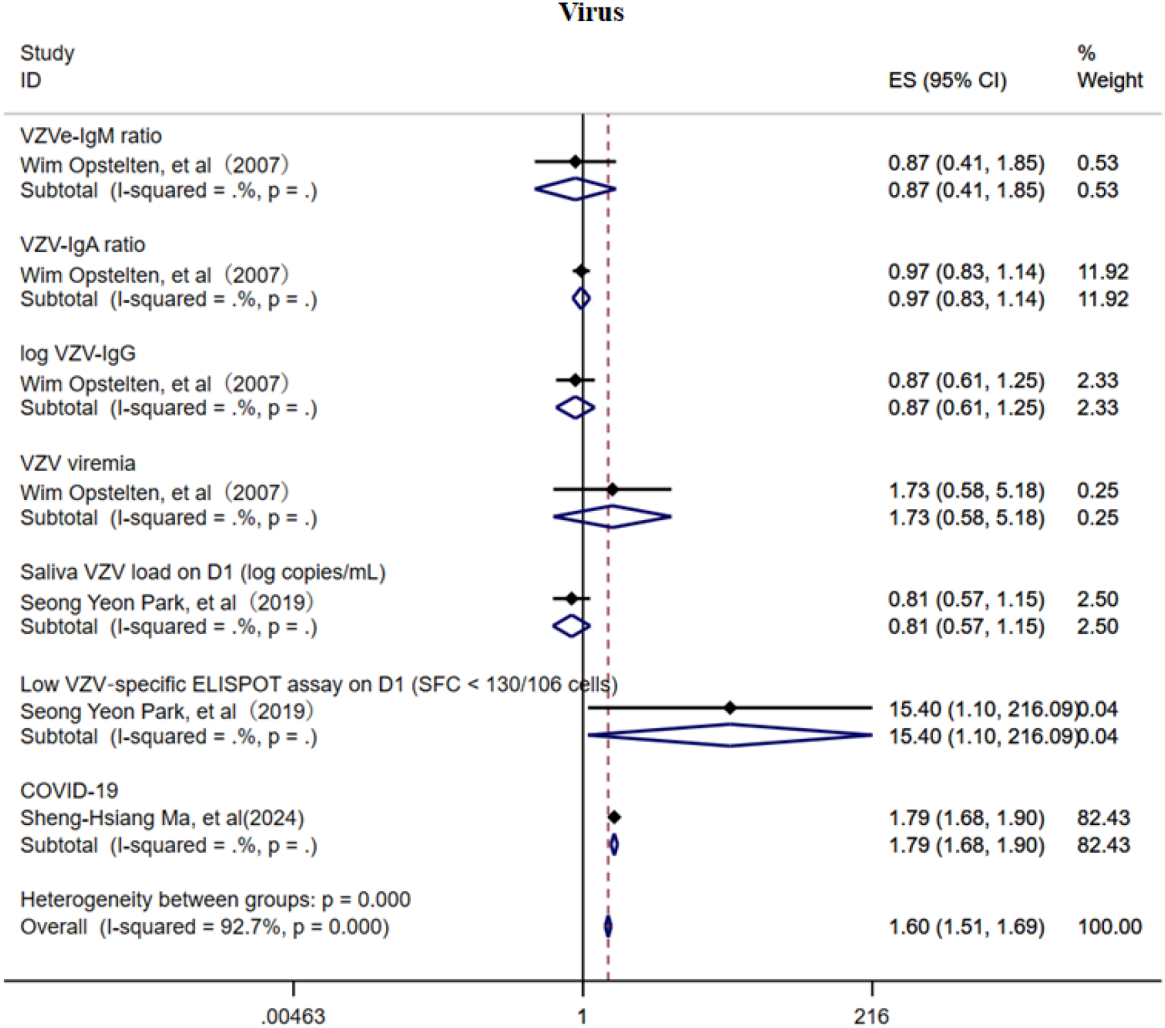
Meta-analysis of virus as a risk factor of PHN.

This study also investigated the association between VZV load, VZV-specific antibodies, and the risk of PHN (**Figure 11**) (15, 20, 34). Due to the limited number of included studies, current evidence suggests that VZV-specific antibodies (IgM, IgA, and IgG) may offer a degree of protective effect against PHN, whereas elevated VZV viral load may be a potential risk factor. In addition, the COVID-19 infection may increase the risk of PHN (OR = 1.79, 95% CI: 1.68–1.90). However, given the small number of studies available, these factors did not demonstrate consistent subgroup effects, and the strength of the evidence remains insufficient. The sensitivity analysis revealed that only the study related to COVID-19 substantially influenced the pooled effect estimate (**Supplementary Information**), suggesting a notable impact of that study on the overall result. The Egger’s test and funnel plot (**Supplementary Figure 17**) analysis showed no publication bias (p > 0.05, **Supplementary Information**).

### 3.5 Comorbidity

#### 3.5.1 Type2 diabetes mellitus

**Figure 12:**
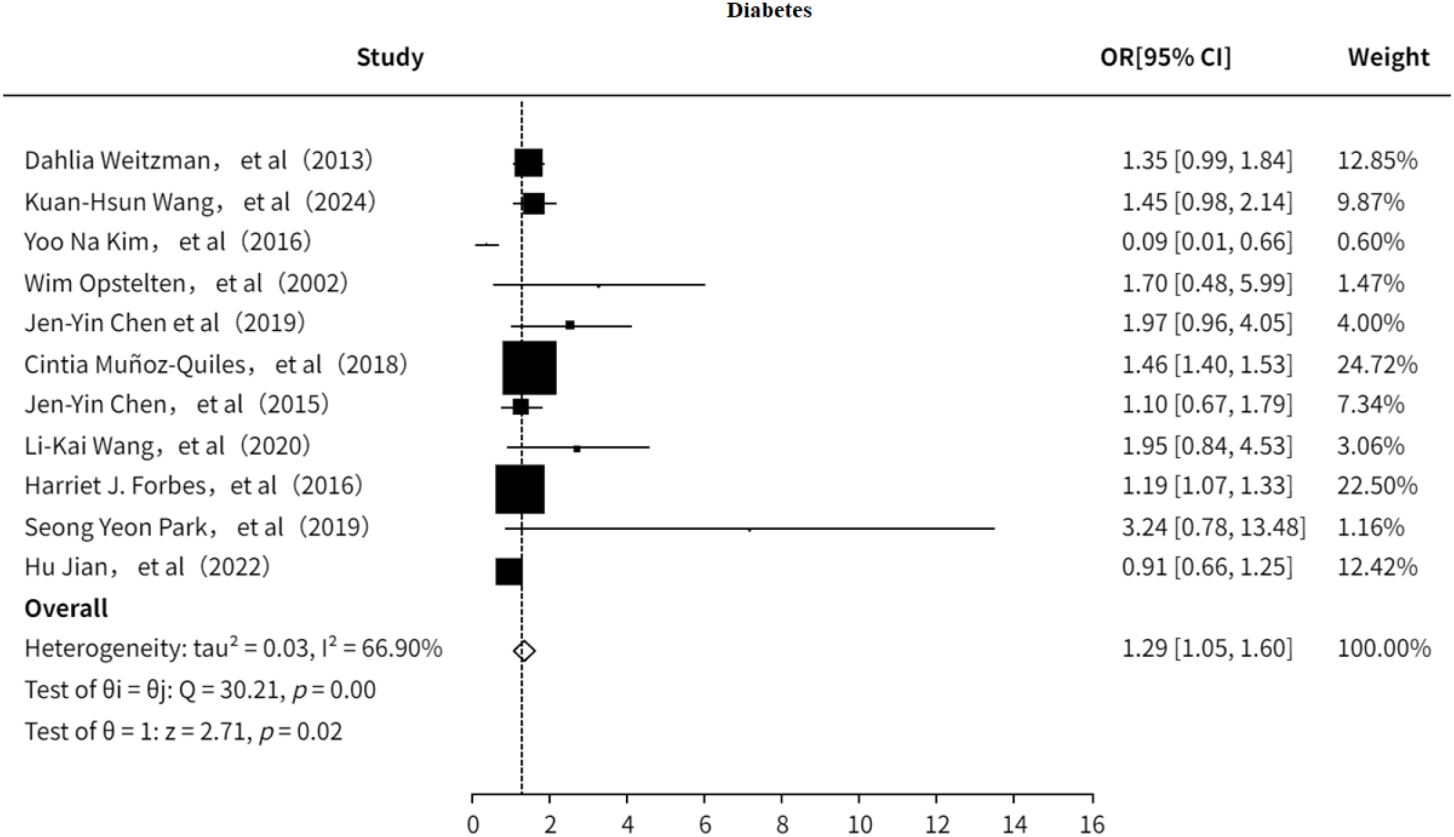
Meta-analysis of diabetes mellitus as a risk factor of PHN.

The study also investigated the association between T2DM and PHN (7, 9, 13, 18–21, 23, 24, 32, 33). The results indicated (**Figure 12**) that T2DM is a risk factor for PHN (OR = 1.29, 95% CI: 1.05–1.60), although moderate heterogeneity was observed (I² = 66.90%). Sensitivity analysis (**Supplementary Information**) showed that no single study significantly influenced the pooled effect size, suggesting the results are relatively robust. Furthermore, both the funnel plot (**Supplementary Figure 18**) and Egger’s test (**Supplementary Information**) revealed no significant publication bias.

### 3.5.2 Cancer history

**Figure 13:**
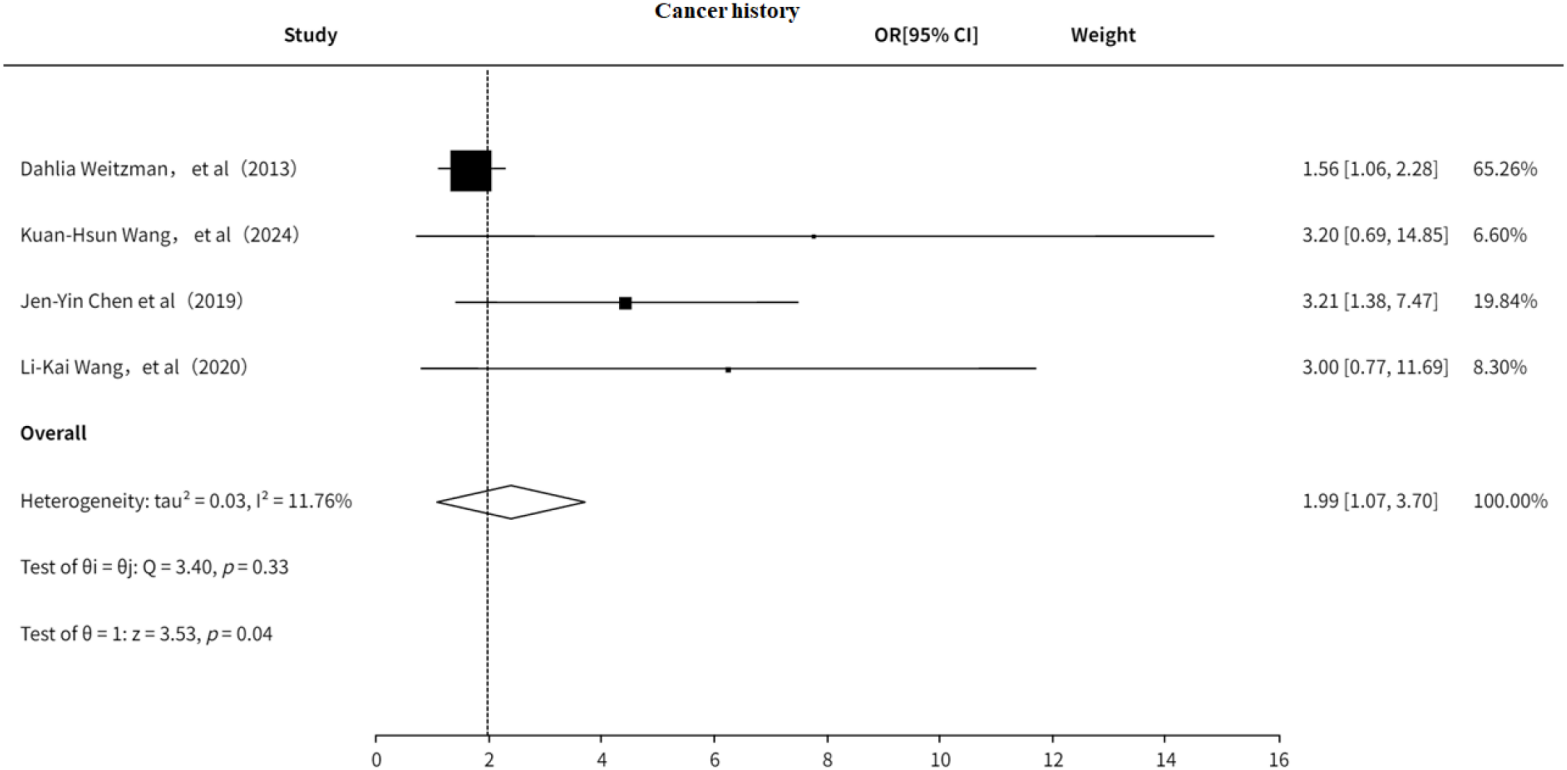
Meta-analysis of cancer history as a risk factor of PHN.

The study also explored the association between cancer history and PHN. The results (**Figure 13**) indicated that a history of cancer is a risk factor for PHN (OR = 1.99, 95% CI: 1.07–3.70), with no significant heterogeneity observed. Additionally, the funnel plot (**Supplementary Figure 20**) revealed significant publication bias.

#### 3.5.3 Chronic kidney disease

**Figure 14:**
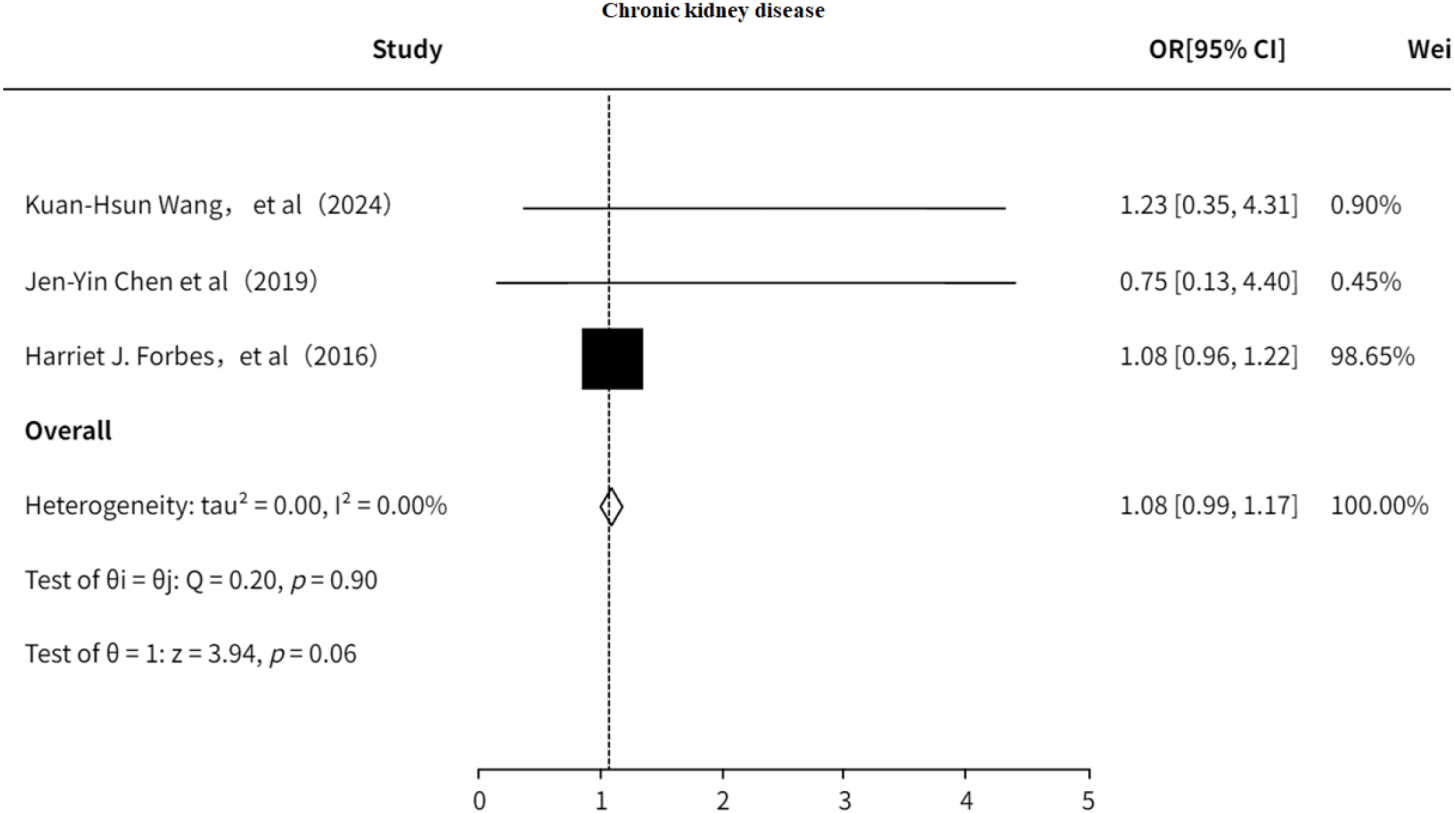
Meta-analysis of CKD as a risk factor of PHN.

This study examined the association between CKD and PHN (9, 18, 33). The results suggested (**Figure 14**) that CKD may be a risk factor for PHN (OR = 1.08, 95% CI: 0.99–1.17); however, the association was not statistically significant, and no significant heterogeneity was detected. The funnel plot revealed no significant publication bias (**Supplementary Figure 20**).

#### 3.5.4 COPD

**Figure 15:**
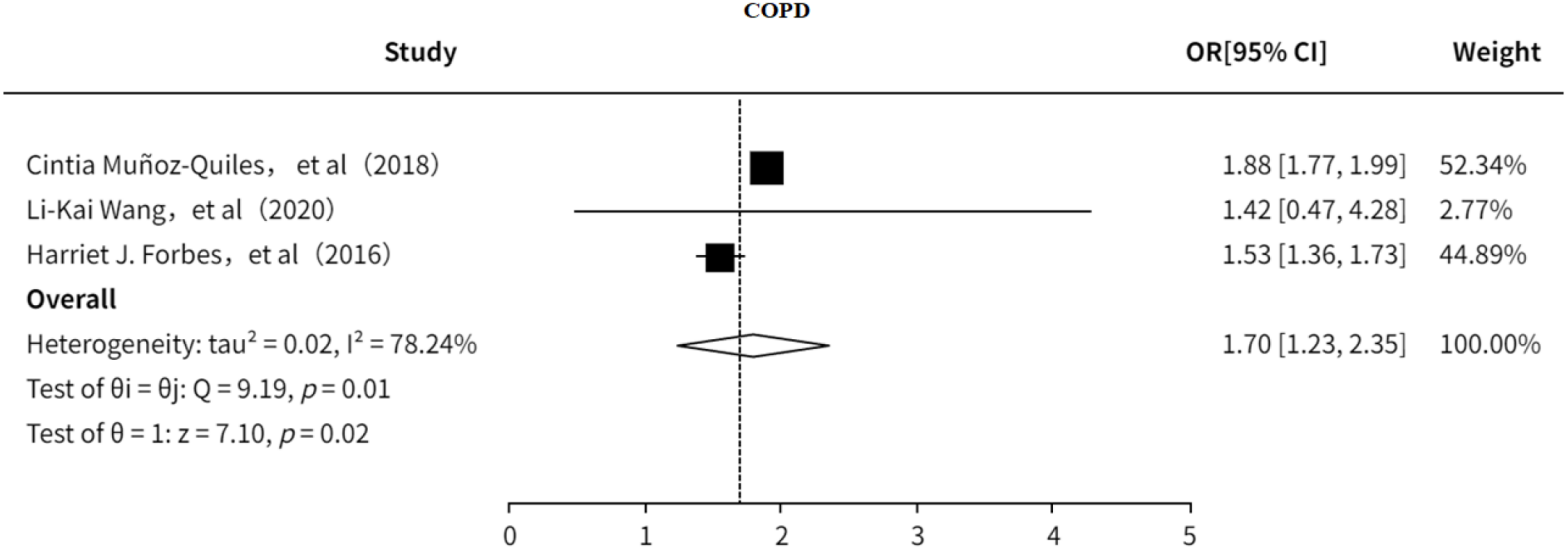
Meta-analysis of COPD as a risk factor of PHN.

This study investigated the association between COPD and PHN (18, 23, 24). The results indicated that (**Figure 15**) COPD is a risk factor for PHN (OR = 1.70, 95% CI: 1.23–2.35), with substantial heterogeneity observed. However, sensitivity analysis (**Supplementary Information**) showed that the pooled effect size remained stable after excluding any single study, suggesting that the findings are relatively robust. No significant publication bias was detected (**Supplementary Figure 21**).

#### 3.5.5 Hypertension

This study explored the association between hypertension and PHN (19, 21, 23, 33). The results indicated that hypertension (**Supplementary Figure 22**) is a risk factor for PHN (OR = 1.82, 95% CI: 1.28–2.58), with no significant heterogeneity observed. Furthermore, neither the funnel plot nor Egger’s test revealed significant publication bias (**Supplementary Figure 23**, **Supplementary Information**).

## 4 Discussion

### 4.1 Main findings

This systematic review included 15 prospective studies, 5 case-control studies, 13 retrospective studies, and 3 systematic reviews, to comprehensively investigate the risk factors for PHN. Current evidence indicates that including older age and female sex among demographic factors, and including smoking and alcohol consumption among lifestyle factors, are associated with an increased risk of PHN. Regarding clinical features, greater severity of rash-related pain, the timing of disease onset, and the acute phase of HZ have all been identified as significant risk factors for PHN, whereas rash occurring in nerve-related areas may confer a certain protective effect. Moreover, pain assessment scales such as the VAS and the NRS can also serve as predictors of PHN risk. In terms of treatment-related factors, antiviral therapy against HZ is considered protective, whereas immunotherapy may significantly increase the risk of PHN. From a virological perspective, the type and concentration of serum antibodies to VZV may exert a protective effect against PHN. However, because the evidence is limited to a small number of studies, COVID-19 cannot yet be considered a definitive risk factor for PHN and remains a possible association requiring confirmation. Comorbidities such as T2DM, malignancies, CKD, COPD, and hypertension have also been shown to be associated with an elevated risk of PHN. This review systematically summarizes the risk factors for PHN from multiple perspectives, including demographic characteristics, clinical examinations and symptoms, virological factors, comorbidities, and treatment-related factors. It can provide clinicians with valuable information to identify high-risk populations for PHN and to implement early interventions, thereby offering practical guidance and population-based evidence for the prevention of PHN.

### 4.2 Potential mechanisms of PHN pathogenesis and their relationship to the current evidence

The development of PHN involves multilevel pathophysiological mechanisms. In the early stages of the disease, the reactivation of latent VZV in sensory ganglia (such as the dorsal root ganglia or trigeminal ganglia) triggers a significant inflammatory response, leading to damage to peripheral nerve tissues (35). This persistent nerve injury further induces a series of changes in the central nervous system. On the one hand, dorsal horn neurons become hyperexcitable due to prolonged exposure to abnormal afferent signals; on the other hand, changes in synaptic plasticity induced by nerve injury promote structural remodeling of the spinal dorsal horn (4). These mechanisms collectively contribute to the persistence of chronic pain. Thus, the pathogenesis of PHN essentially results from the combined effects of direct viral damage and secondary nerve injury, reflecting the complex interplay between the peripheral and central nervous systems in the pathological process.

Our findings confirm that the severity of HZ rashes, along with acute pain intensity, are significant risk factors for PHN, consistent with the prevailing hypothesis that greater neural damage predisposes to chronic pain (5, 36). However, the paradoxical protective effect observed with neural involvement suggests anatomical and pathophysiological heterogeneity underlying PHN development. This may be attributed to differential viral tropism, neural repair capacity, or inflammatory responses among distinct neural subtypes (e.g., cranial vs. spinal nerve**s**) (37). Age remains the most robust predictor of PHN (38), likely due to immune senescence and diminished axonal regeneration in older adults. Early antiviral therapy demonstrated significant protective effects, reinforcing current clinical guidelines, while immunosuppression emerged as a critical modifiable risk factor (39). While current evidence is insufficient to establish COVID-19 as a risk factor for PHN, the potential for post-viral immune dysregulation suggests that COVID-19 may increase PHN risk and therefore merits further study. These results underscore the multifactorial nature of PHN pathogenesis, involving complex interactions between viral, neural, and immune mechanisms. Future studies should explore targeted interventions for high-risk populations, including enhanced antiviral protocols and immunomodulatory strategies.

### 4.3 Strengths and innovations

Our findings are largely consistent with previous studies. In terms of demographic factors, older age, particularly ≥60 years, was confirmed as a significant risk factor for PHN, in line with prior evidence. Similarly, although female showed a weak association in this study, the wide confidence interval (95% CI: 0.93–1.47) indicates that its role as a risk factor remains inconclusive, which is also consistent with previous reports. Regarding clinical features, this study confirmed that prodromal pain, severe acute-phase pain, severe rash, extensive skin involvement, and delayed antiviral therapy are well-recognized risk factors for PHN, further supporting the notion that PHN development is closely related to clinical characteristics.

Importantly, compared with previous work, this study updates and broadens the spectrum of PHN risk factors. In the clinical domain, earlier studies mainly linked rash severity or overall disease severity with PHN. Our findings demonstrate that higher NOS and VAS pain scores are also significantly associated with increased risk. From a virological perspective, serum VZV-specific antibodies such as IgG and IgM may be a protective effect, although the supporting evidence remains limited. COVID-19 was evaluated for the first time as a potential risk factor. Because the available studies are few, the current evidence only suggests a possible association that requires confirmation. Socioeconomic status also emerged as a candidate factor. However, findings were inconsistent, as some studies associated higher income with greater PHN risk while others reported the opposite, making the relationship inconclusive.

Our analysis further suggests that malignancy may increase PHN risk, which is consistent with prior indications that did not reach significance due to small sample sizes. Earlier meta-analyses did not confirm T2DM as a risk factor. By including more recent studies and applying a random-effects model, our study provides preliminary evidence supporting this association. Additional comorbidities, including COPD and hypertension, were identified as possible risk factors, while CKD showed only a suggestive trend and did not achieve statistical significance.

In terms of treatment, we found that delayed antiviral therapy and immunosuppressive treatment significantly increased the risk of PHN, consistent with previously published studies. In addition, several serological markers, including IL-8, myelin basic protein, CD8+ cell proportion, IL-6, Galectin-3, vitamin D, and vitamin C, were preliminarily associated with PHN risk. By integrating modifiable therapeutic strategies with emerging serological indicators, our study provides the first comprehensive framework for risk assessment with potential implications for targeted prevention. However, given the limited number of studies, further validation is required to establish the predictive value of these markers.

### 4.4 The implications of our findings

Based on the risk factors identified in this study, we recommend that the prevention and management of PHN should emphasize early intervention and individualized care. Older age (≥60 years) emerged as a clear risk factor, and elderly patients often present with multiple comorbidities such as malignancy, COPD, hypertension, and T2DM. Although the strength of evidence for these comorbidities is relatively modest, they are highly prevalent in older populations and warrant particular attention. In these high-risk patients, prompt recognition of HZ and the initiation of antiviral therapy within 72 hours are essential to reduce PHN risk. While the role of sex remains inconclusive, most studies suggest a higher risk among females. Therefore, elderly women should be considered a reference subgroup in clinical risk stratification. For immunocompromised patients, it is crucial to balance the requirements of underlying disease management with timely and adequate antiviral therapy once HZ occurs. In certain cases, extending the duration of antiviral treatment according to individual immune status may help minimize the risk of persistent viral replication.

Furthermore, this study confirmed that acute-phase pain severity is strongly associated with subsequent PHN, underscoring the need for early multimodal analgesia and intervention to prevent the transition from acute to chronic neuropathic pain. Notably, our findings also suggest that HZ specific antibodies (e.g., IgG, IgM) may exert a protective effect. This observation provides a rationale for considering HZ vaccination as a preventive strategy in uninfected individuals or those at high risk. In addition, COVID-19 emerged as a potential risk factor for PHN. Although current evidence remains limited, careful documentation of infection history is warranted, particularly in the post-pandemic era, to provide epidemiological insights into the possible long-term impact of COVID-19 on PHN incidence and to inform preventive measures.

### 4.5 Limitation and future direction

This study has several limitations. First, the current evidence on certain risk factors (such as history of COVID-19 infection, socioeconomic status, and inflammatory serological markers) remains relatively limited, which may affect the robustness of the conclusions. Second, most included studies relied on clinical diagnoses and lacked unified serological or virological detection indicators to confirm herpes virus infection. Such diagnostic discrepancies may lead to case classification bias. Third, the available evidence predominantly comes from observational studies, making it difficult to fully control for confounding factors, thereby limiting the reliability of causal inferences. Fourth, significant heterogeneity was observed among some studies, posing challenges for the integration and interpretation of results.

Based on these limitations, future research could focus on the following directions: In the post-pandemic era, various SARS-CoV-2 variants may have differential impacts on certain populations. Therefore, it is necessary to conduct large-scale, multi-center studies to further validate the association between COVID-19 and the occurrence of PHN. Second, objective biomarkers should be combined with standardized assessment tools to improve the reliability and comparability of soft indicators such as socioeconomic status. Third, it is essential to strengthen control over potential confounding variables and implement methods such as PCR for the confirmation of HZ infection to enhance diagnostic precision. Finally, cross-regional studies involving diverse populations are essential for improving the generalizability and broader applicability of the results.

## Data Availability

The original contributions presented in the study are included in the article/**Supplementary Material**. Further inquiries can be directed to the corresponding author.

## Supplementary material

Supplementary Figure 1 The subgroup analyze of age as risk for PHN.

Supplementary Figure 2 Funnel plot of age study.

Supplementary Figure 3 Meta-analysis of gender as a risk factor of PHN.

Supplementary Figure 4 Funnel plot of gender study.

Supplementary Figure 5 The subgroup analyze of social economic as risk for PHN.

Supplementary Figure 6 Funnel plot of social economic study.

Supplementary Figure 7 The subgroup analyze of life history as risk for PHN.

Supplementary Figure 8 Funnel plot of social economic study.

Supplementary Figure 9 The subgroup analyze of clinical as risk for PHN. Forbes HJ, a, Severe rash; b, Rash duration at presentation; Shijuan Wei, a, Brachial plexus; b, Cervical plexus;c, Thoracic nerve; d, Lumbosacral nerve. e, Left. Keiko Sato, a, moderate/severe HZ; b, Upper arms (rash); c, the area of rash ≥3cm^3^. Jen-Yin Chen, The rash location. a, Cervical; b, Thoracic; c, Lumbar and sacral. Hu Jian, The rash location. a, Facial; b, Shoulder and neck; c, Upper arms; d, lower legs. Ding S, The rash location and severe. a, Ophthalmic; b, thoracic nerve; c, nontrunk; d, skin lesion (greater than 5% of the body surface area); e, moderate/severe rash.

Supplementary Figure 10 Funnel plot of social economic study.

Supplementary Figure 11 The subgroup analyze of pain as risk for PHN.

Supplementary Figure 12 Funnel plot of social economic study.

Supplementary Figure 13 The subgroup analyze of clinical index as risk for PHN.

Supplementary Figure 14 Funnel plot of clinical index study.

Supplementary Figure 15 The analyze of therapy factor as risk for PHN. Dahlia Weitzman, HIV therapy; Dahlia Weitzman ^a^, Anti-TNFa; Shijuan Wei, Antiviral drugs, Shijuan Wei^a^, Glucocorticoid; Kuan-Hsun Wang, Metformin, Kuan-Hsun Wang^a^, B-blockers, Kuan-Hsun Wang^b^, SSRIs; Kosuke Kawai, Surgical intervention, Kosuke Kawai^a^, Missed antiviral drugs.

Supplementary Figure 16 Funnel plot of therapy factor study.

Supplementary Figure 17 Funnel plot of virus factor study.

Supplementary Figure 18 Funnel plot of DM factor study.

Supplementary Figure 19 Funnel plot of cancer history factor study.

Supplementary Figure 20 Funnel plot of CKD factor study.

Supplementary Figure 21 Funnel plot of COPD factor study.

Supplementary Figure 22 Meta-analysis of hypertension as a risk factor of PHN.

Supplementary Figure 23 Funnel plot of hypertension factor study.
