# Supplementary material for: Risk factors for postherpetic neuralgia: a meta-analysis based on demographic, clinical features, and treatment characteristics
Source: Front Immunol. 2025 Oct 1;16:1667364. doi: 10.3389/fimmu.2025.1667364 (PMC12521459; doi:10.3389/fimmu.2025.1667364)

sFigure1: The subgroup analyze of age as risk for PHN.


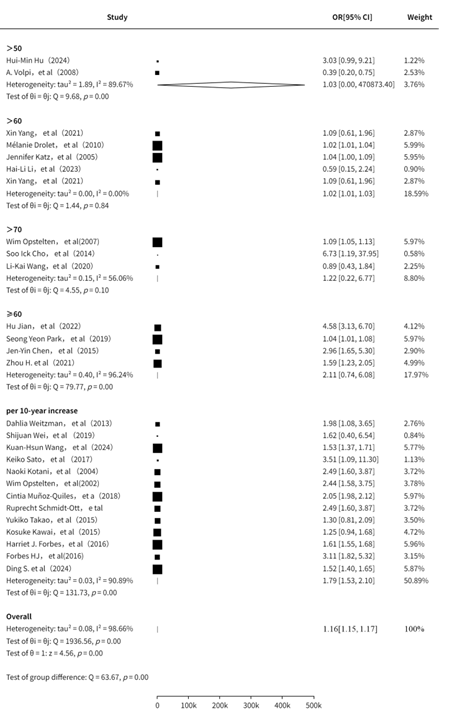


sFigure2: Funnel plot of age study.


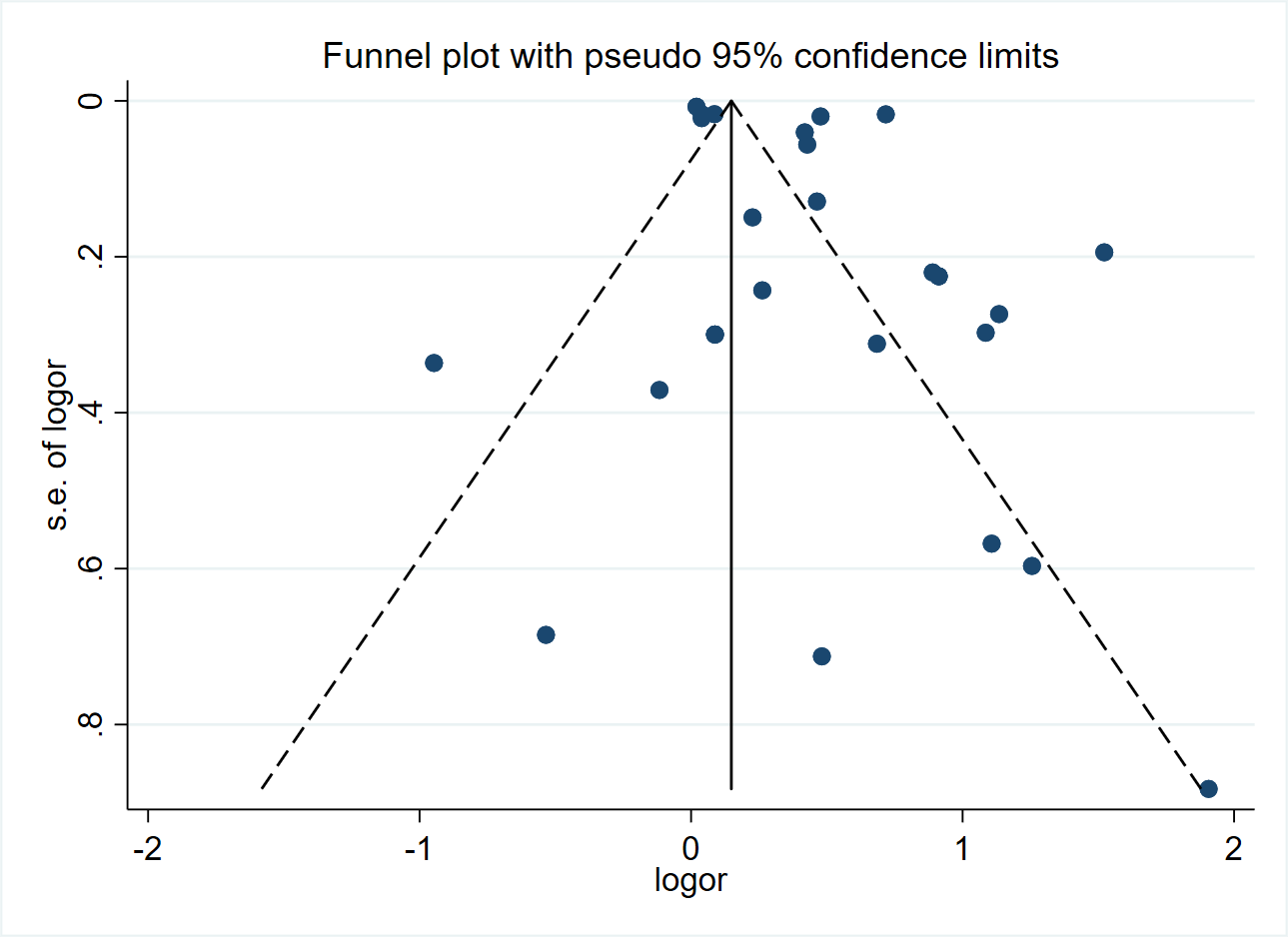


sFigure3: Meta-analysis of gender as a risk factor of PHN


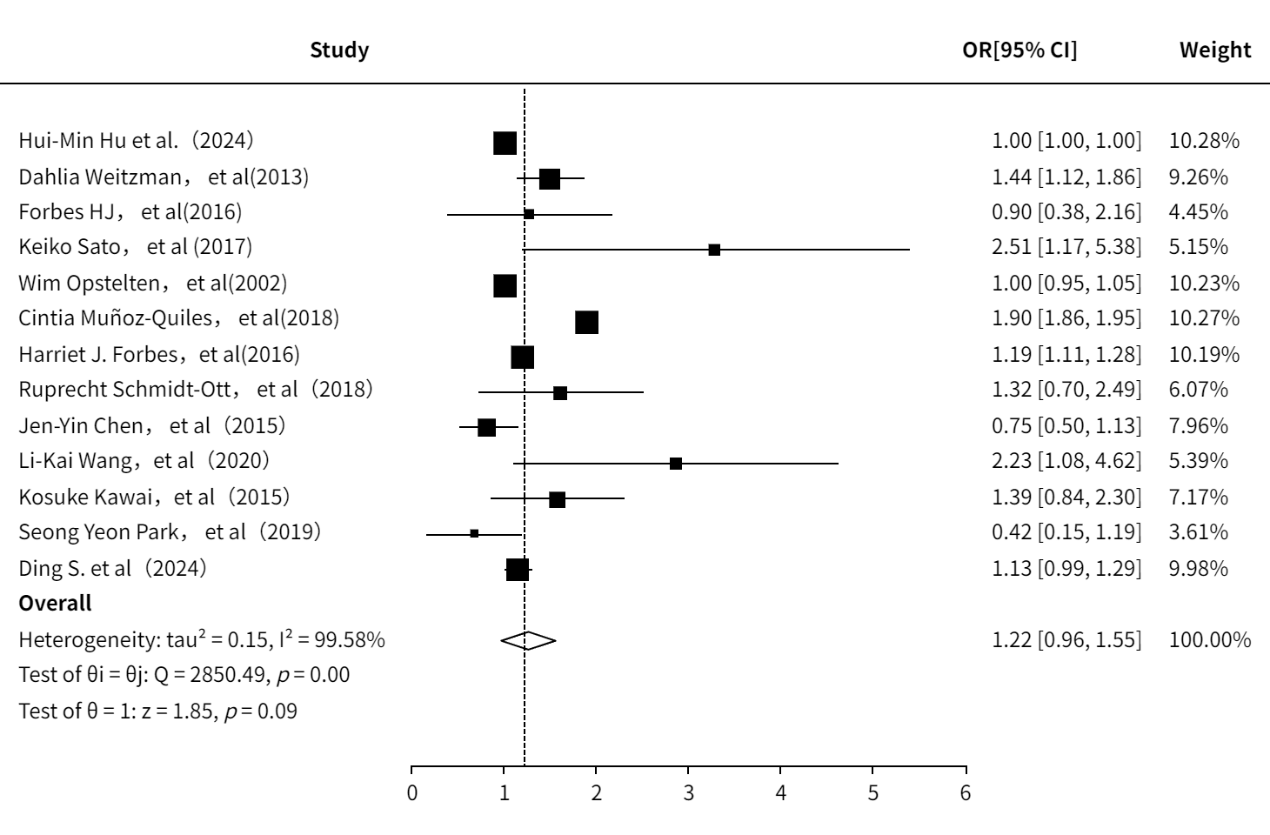


sFigure4: Funnel plot of gender study.


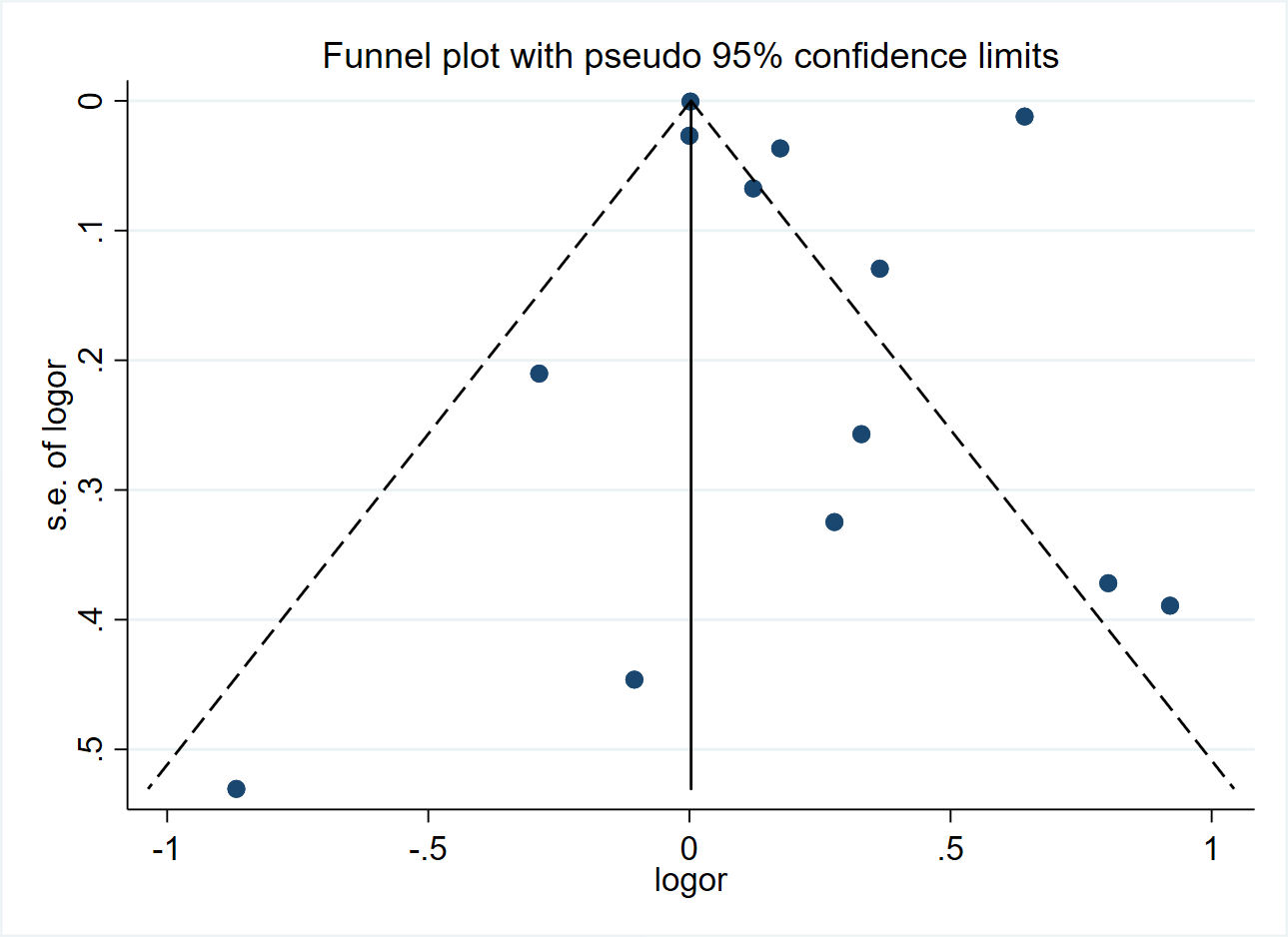


sFigure5: The subgroup analyze of social economic as risk for PHN.


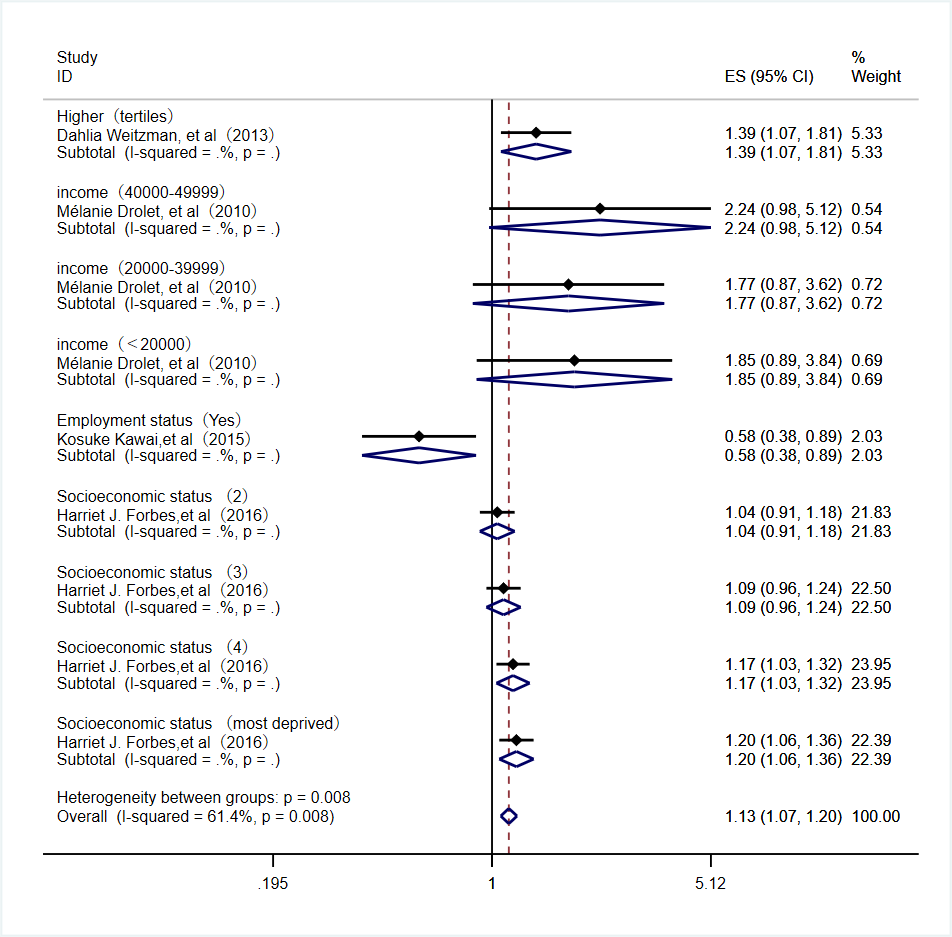


sFigure6: Funnel plot of social economic study


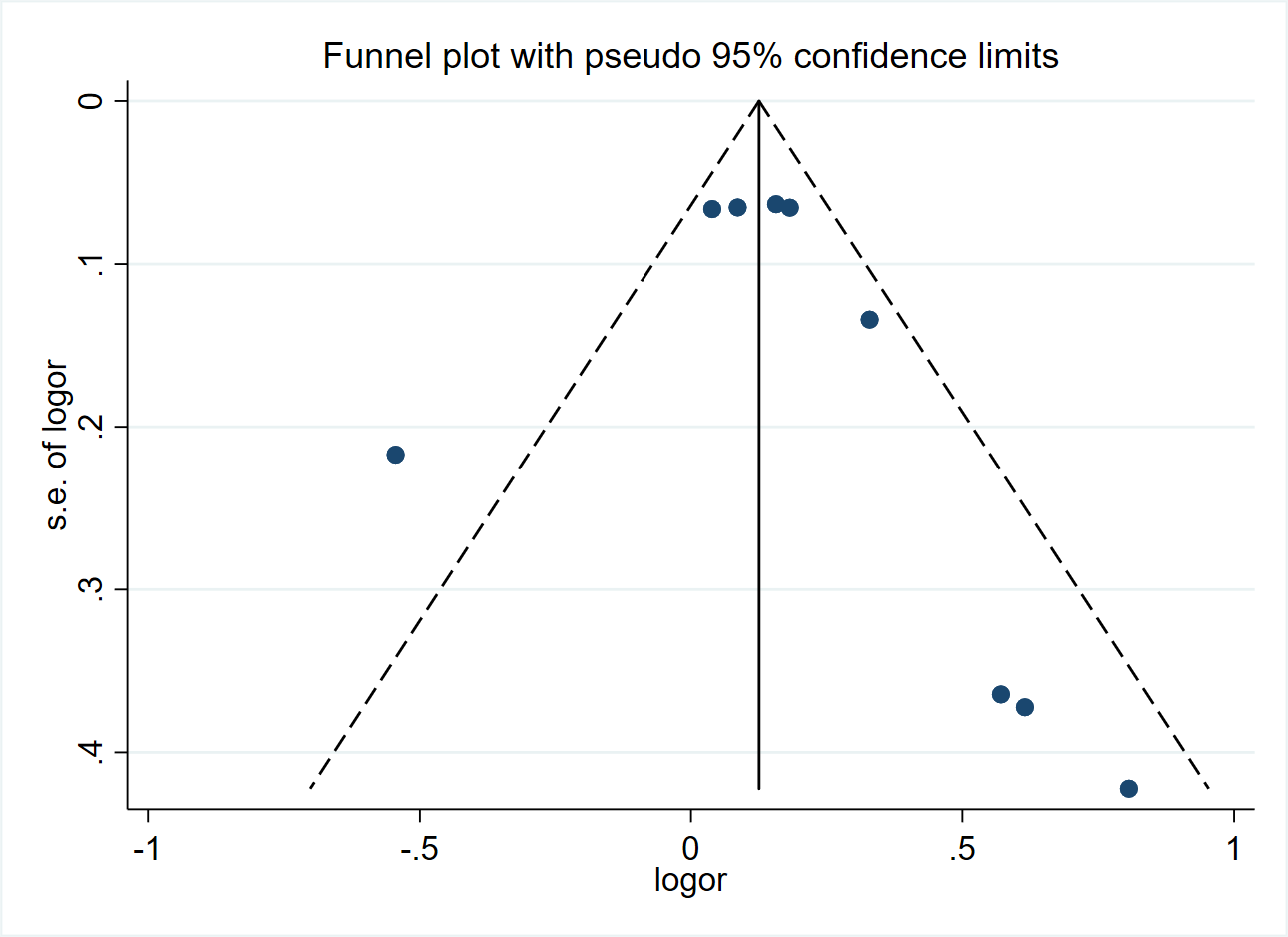


sFigure7: The subgroup analyze of life history as risk for PHN.


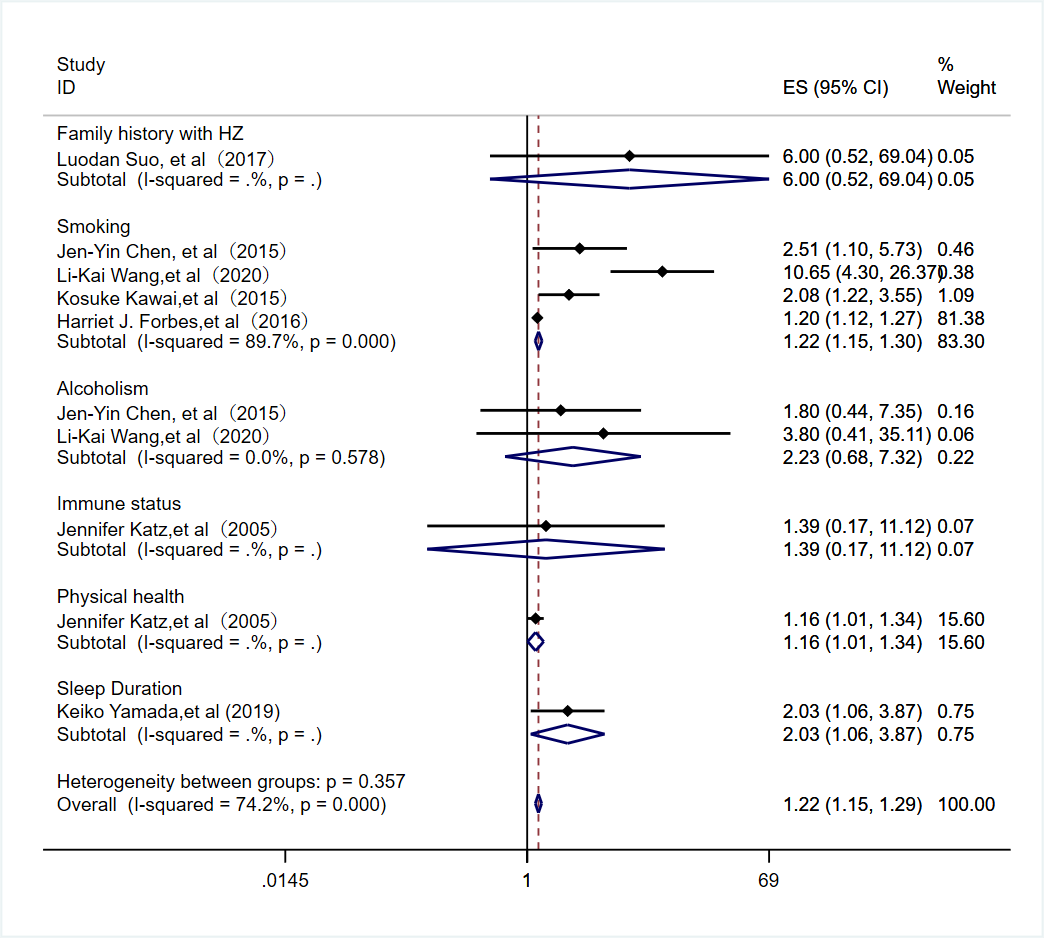


sFigure8: Funnel plot of social economic study.


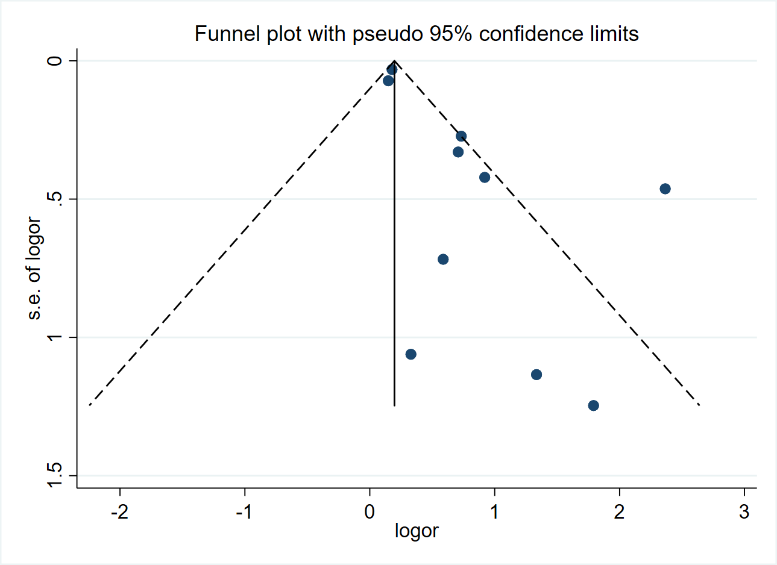


sFigure9: The subgroup analyze of clinical as risk for PHN.

Forbes HJ, a, Severe rash; b, Rash duration at presentation;

Shijuan Wei, a, Brachial plexus; b, Cervical plexus;c, Thoracic nerve; d, Lumbosacral nerve. e, Left.

Keiko Sato, a, moderate/severe HZ; b, Upper arms (rash); c, the area of rash ≥3cm^3^.

Jen-Yin Chen, The rash location. a, Cervical; b, Thoracic; c, Lumbar and sacral.

Hu Jian, The rash location. a, Facial; b, Shoulder and neck; c, Upper arms; d, lower legs.

Ding S, The rash location and servere. a, Opththalmic; b, thoracic nerve; c, nontrunk; d, skin lesion (greater than 5% of the body surface area); e, moderate/severse rash.


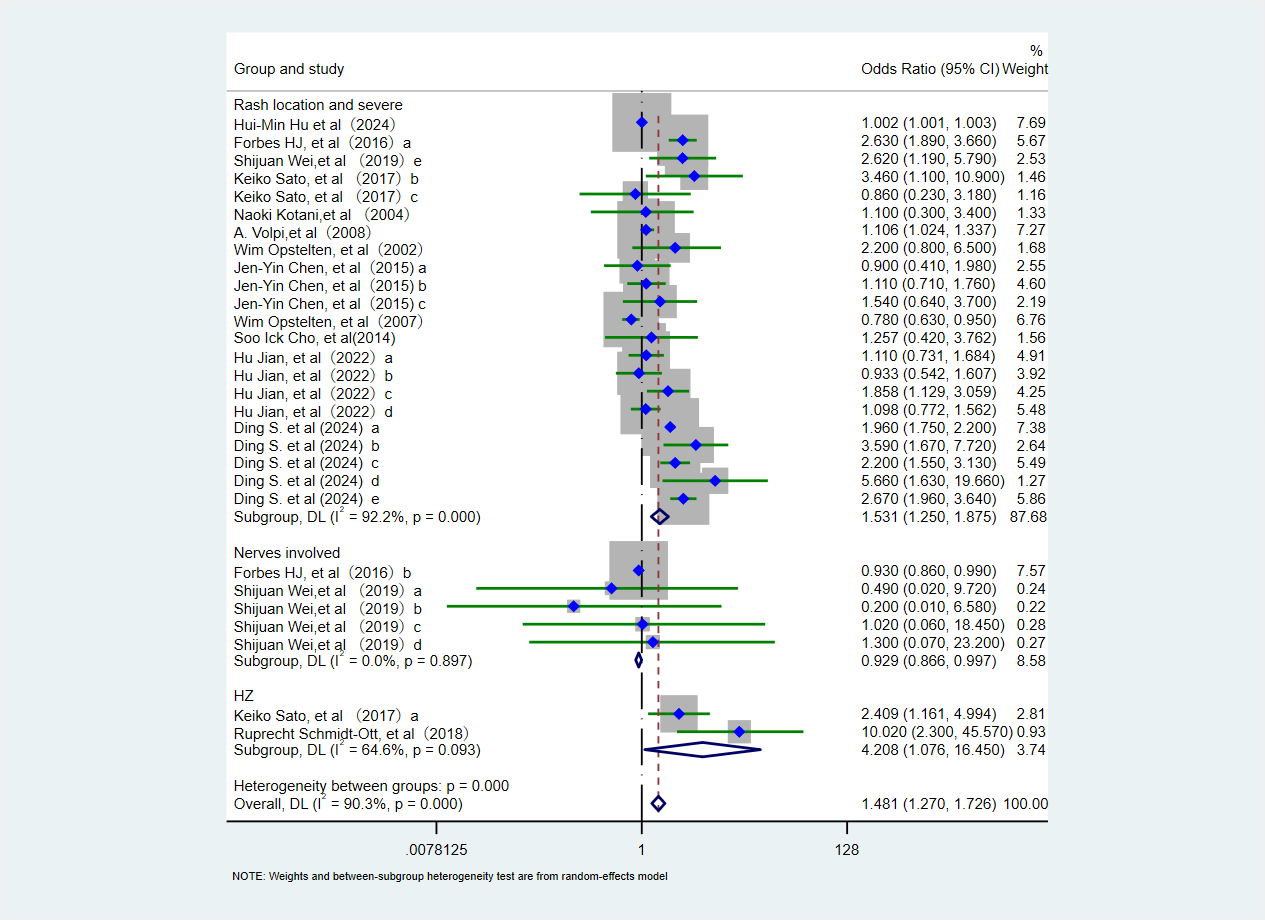


sFigure10: Funnel plot of social economic study.


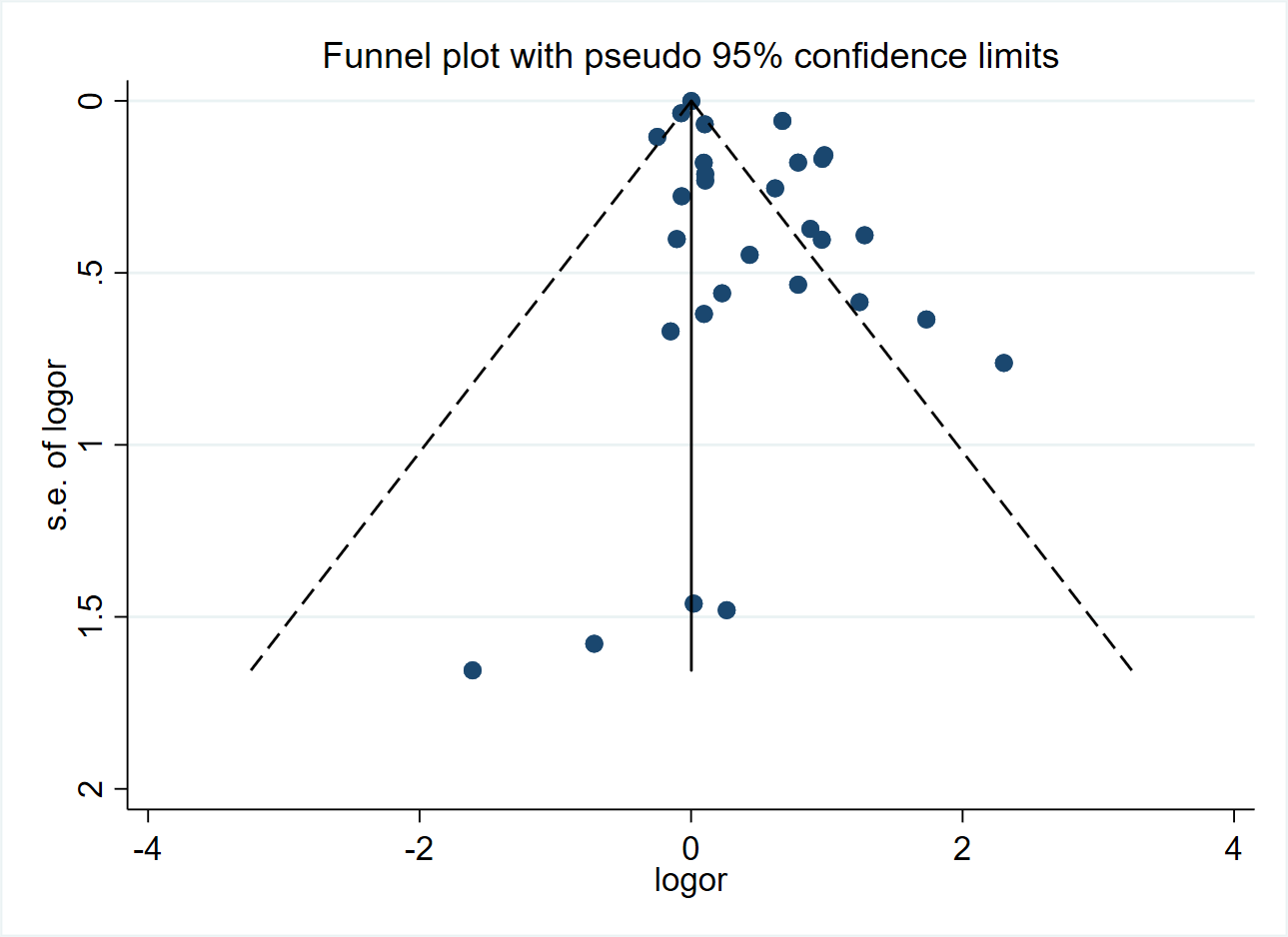


sFigure11: The subgroup analyze of pain as risk for PHN.


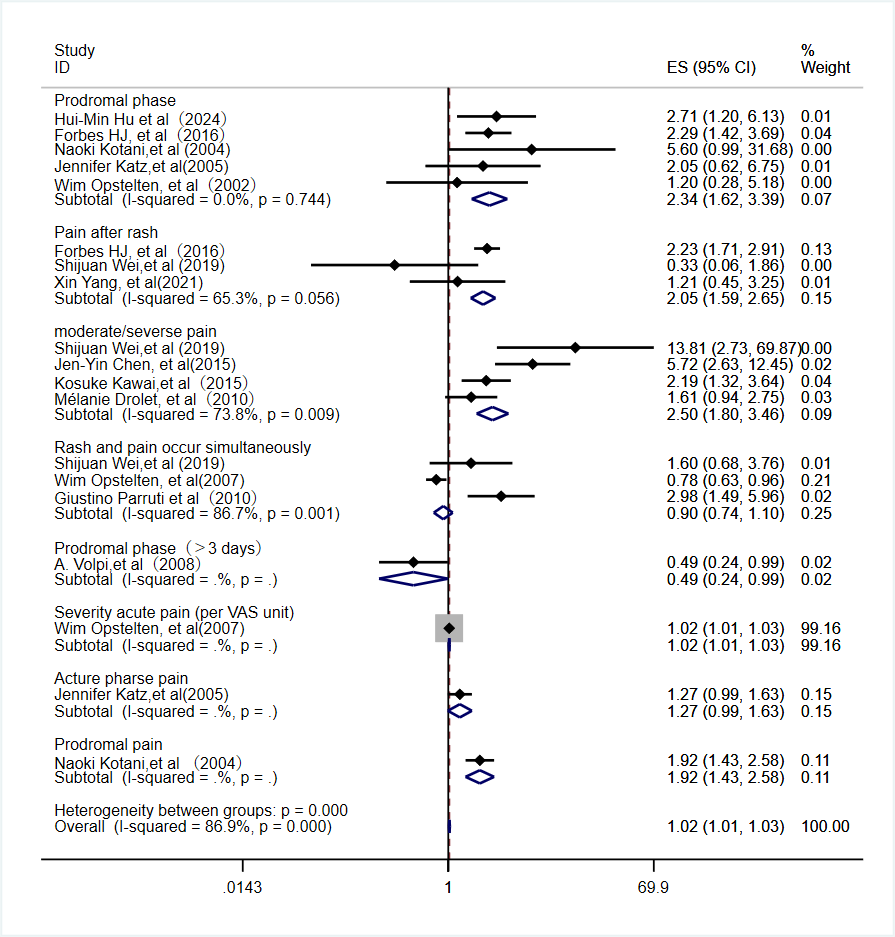


sFigure12: Funnel plot of social economic study.


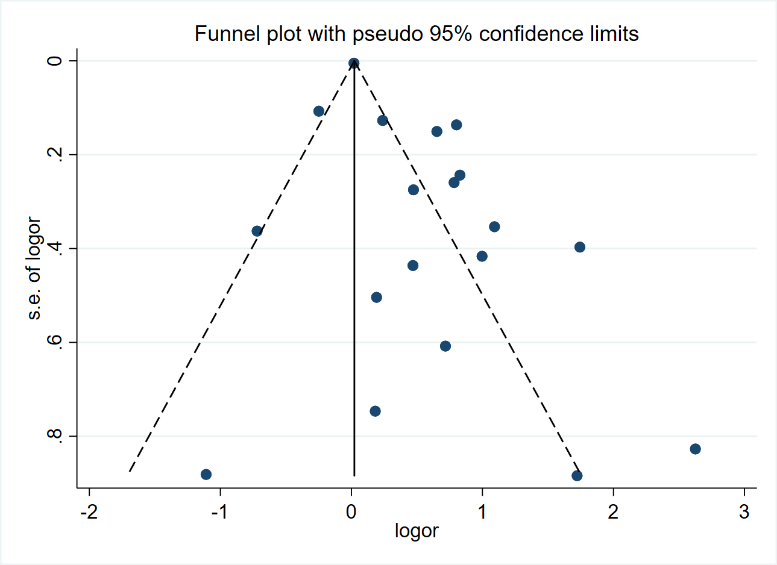


sFigure13: The subgroup analyze of clinical index as risk for PHN.


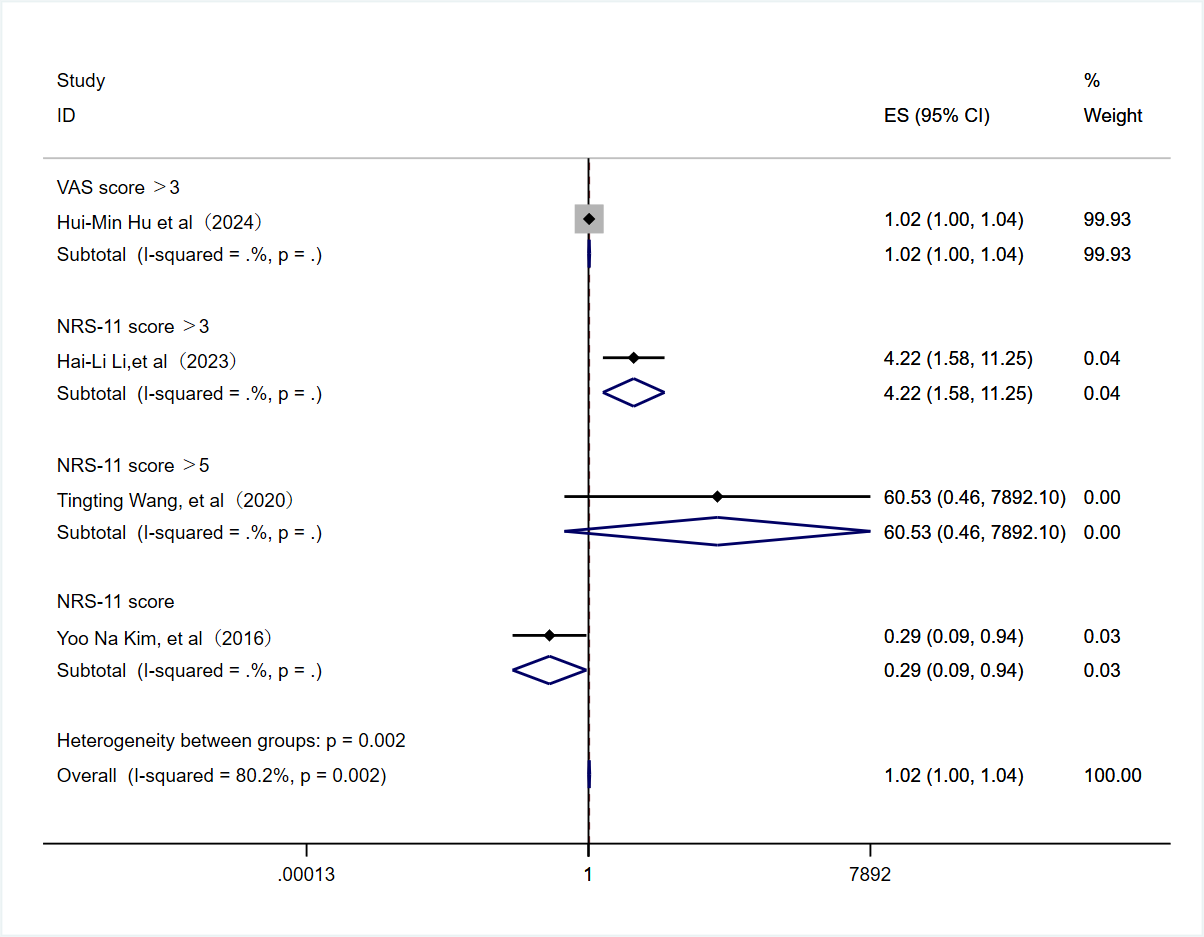


sFigure14: Funnel plot of clinical index study.


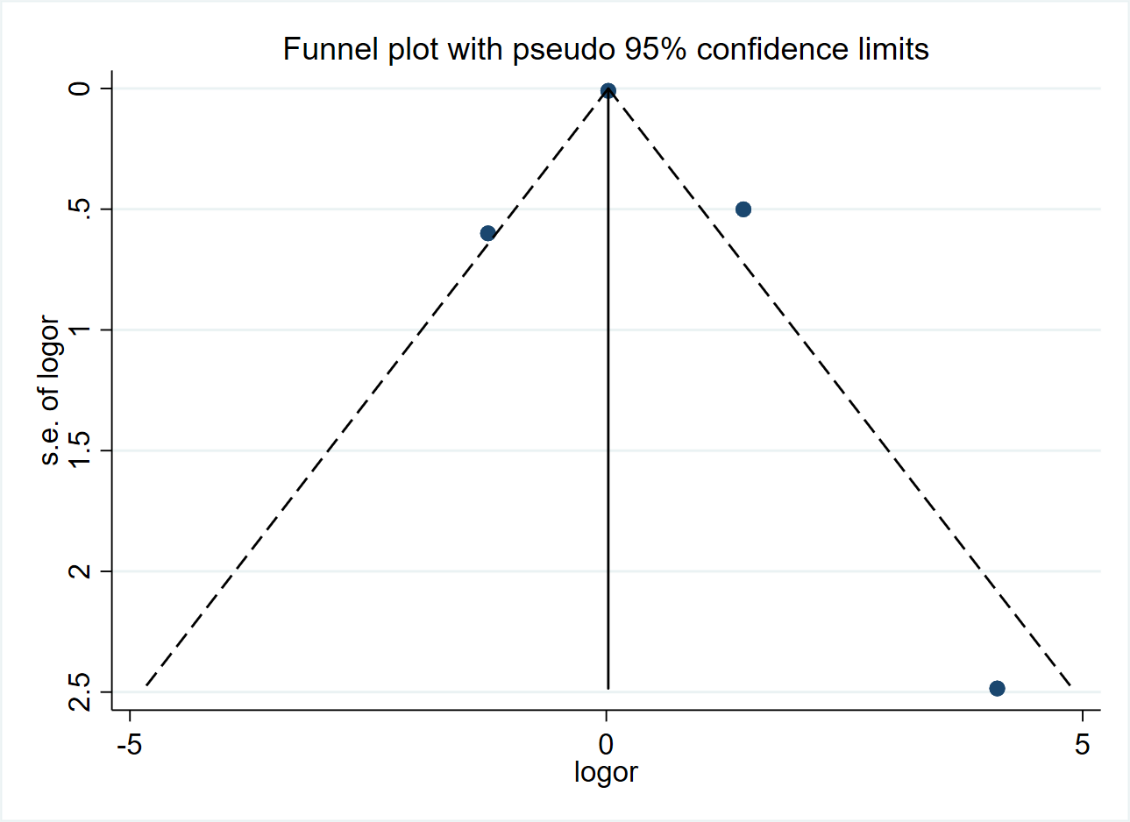


sFigure15: The analyze of therapy factor as risk for PHN.

Dahlia Weitzman, HIV therapy; Dahlia Weitzman ^a^, Anti-TNFa; Shijuan Wei, Antiviral drugs, Shijuan Wei^a^, Glucocorticoid; Kuan-Hsun Wang, Metformin, Kuan-Hsun Wang^a^, B-blockers, Kuan-Hsun Wang^b^, SSRIs; Kosuke Kawai, Surgical intervention, Kosuke Kawai^a^, Missed antiviral drugs.


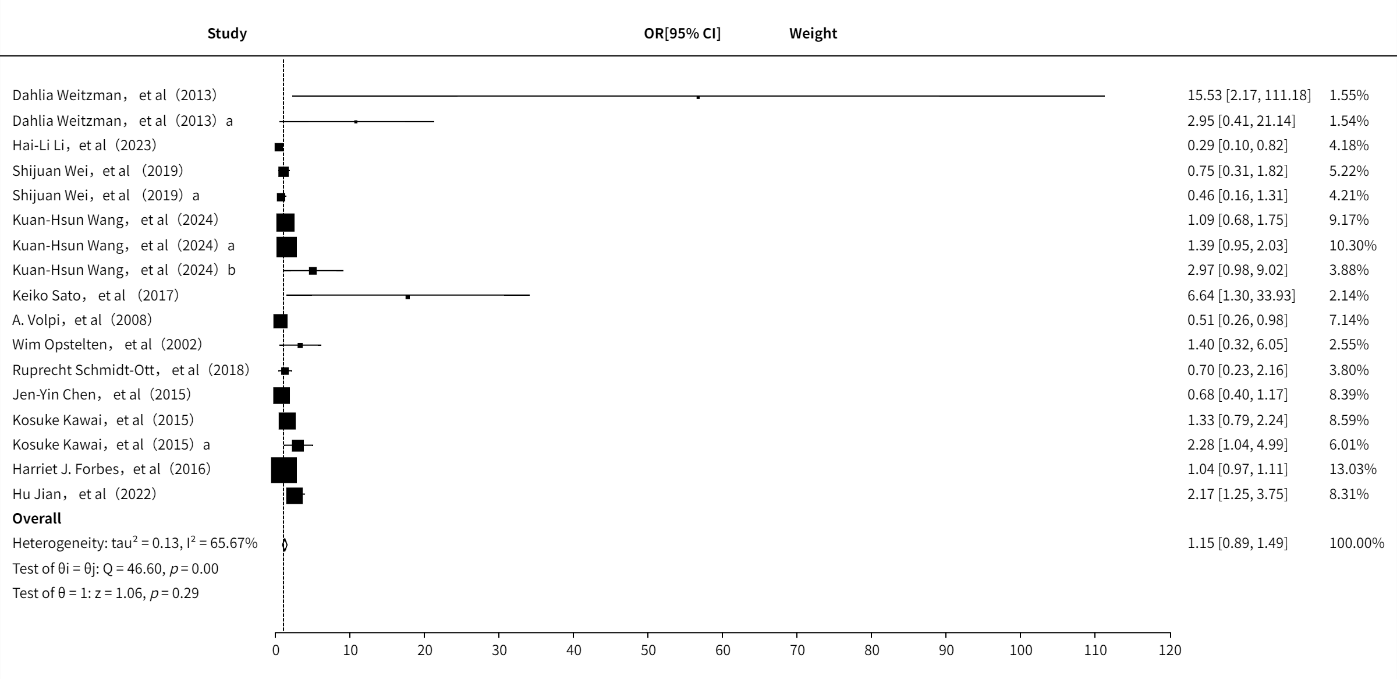


sFigure16: Funnel plot of therapy factor study.


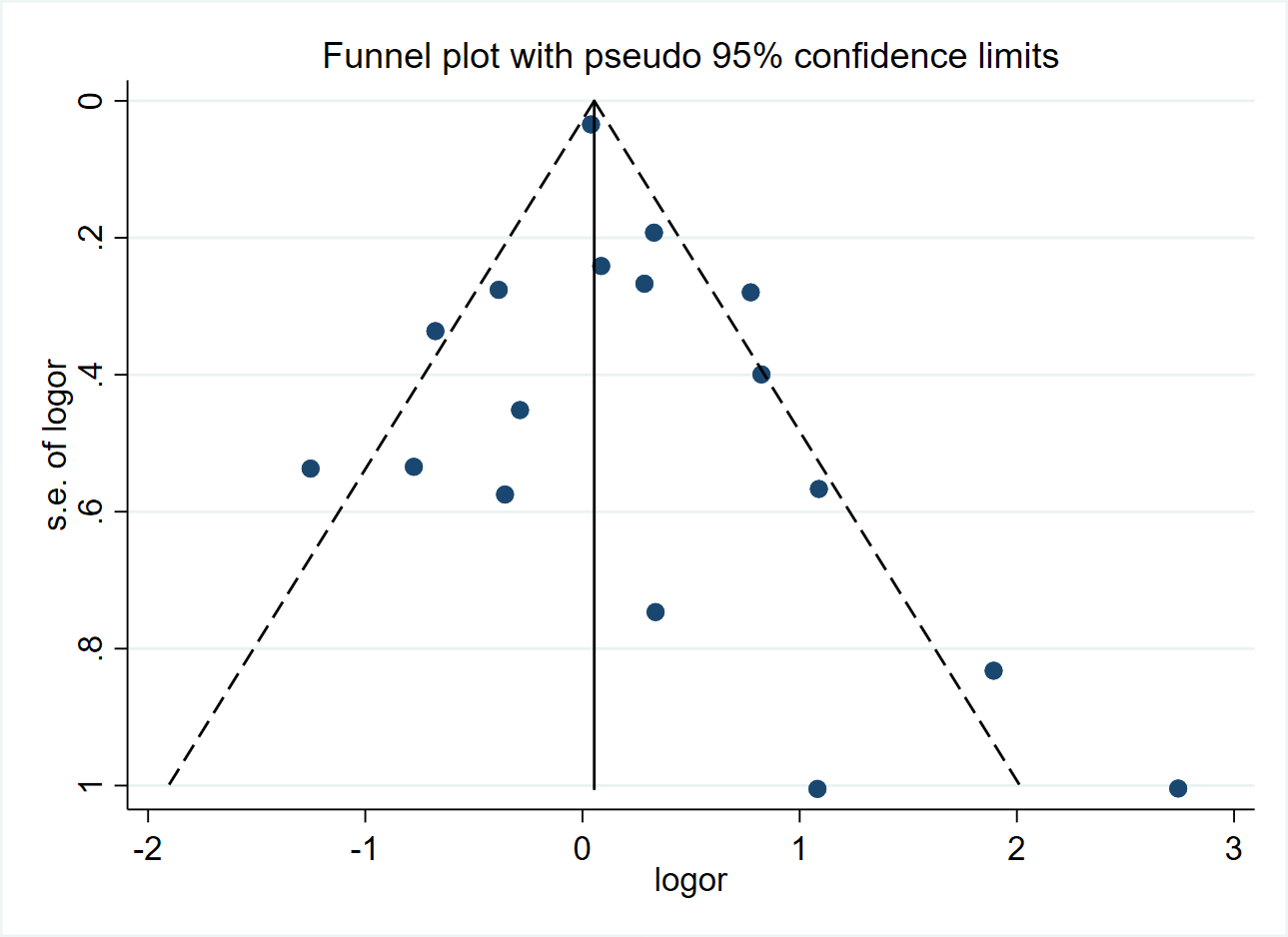


sFigure17: Funnel plot of virus factor study.


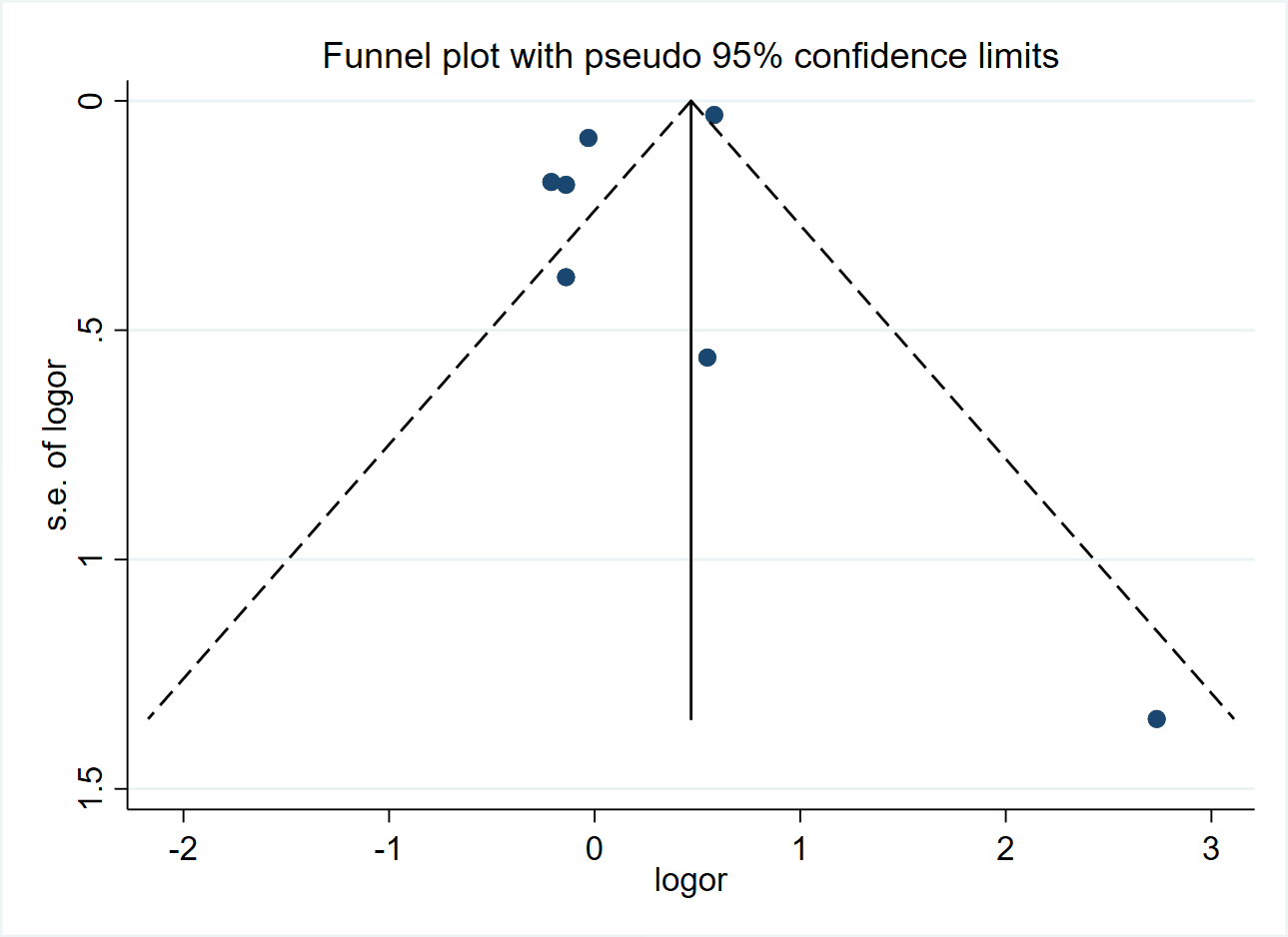


sFigure18: Funnel plot of DM factor study.


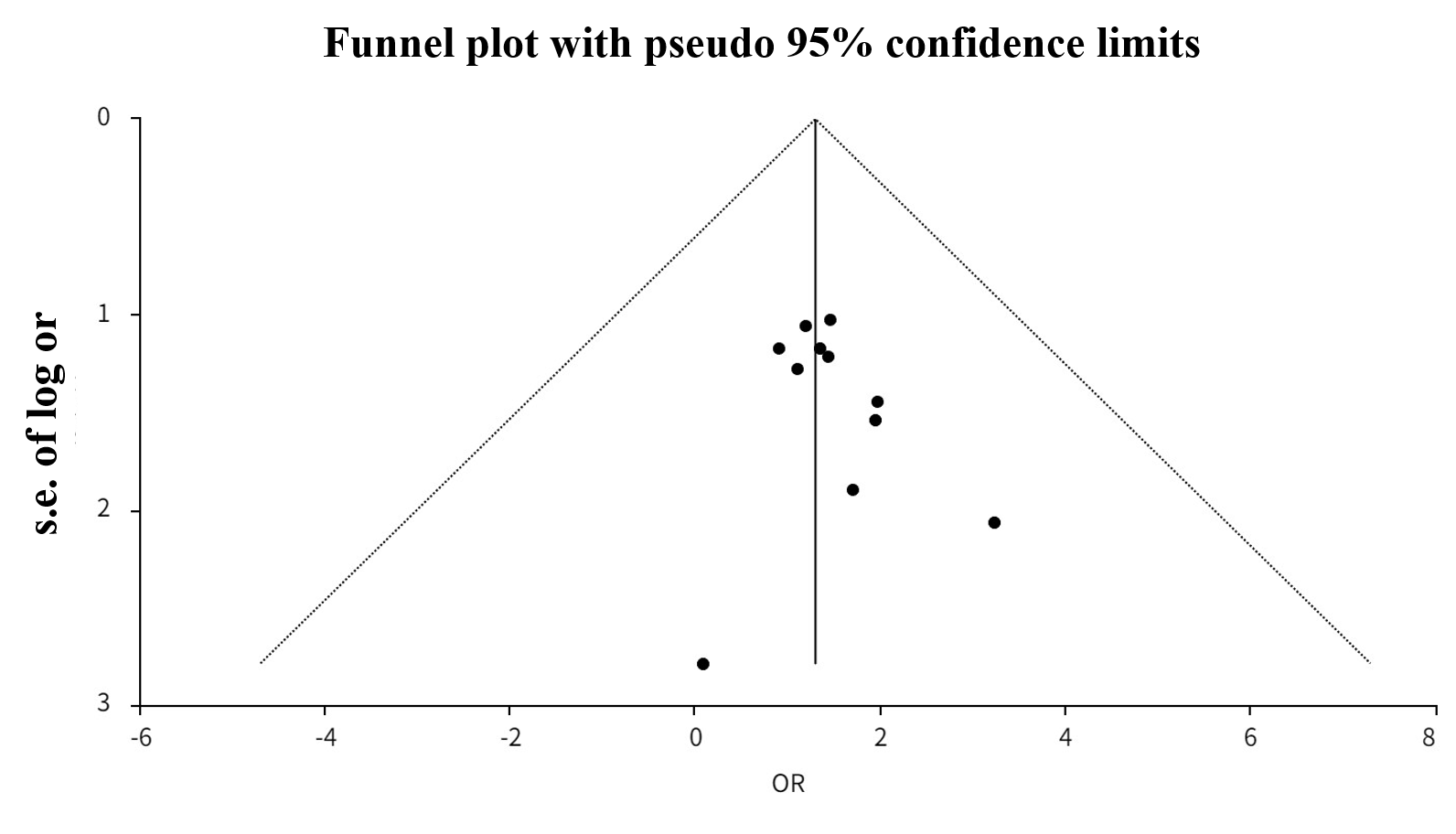


sFigure19: Funnel plot of cancer history factor study.


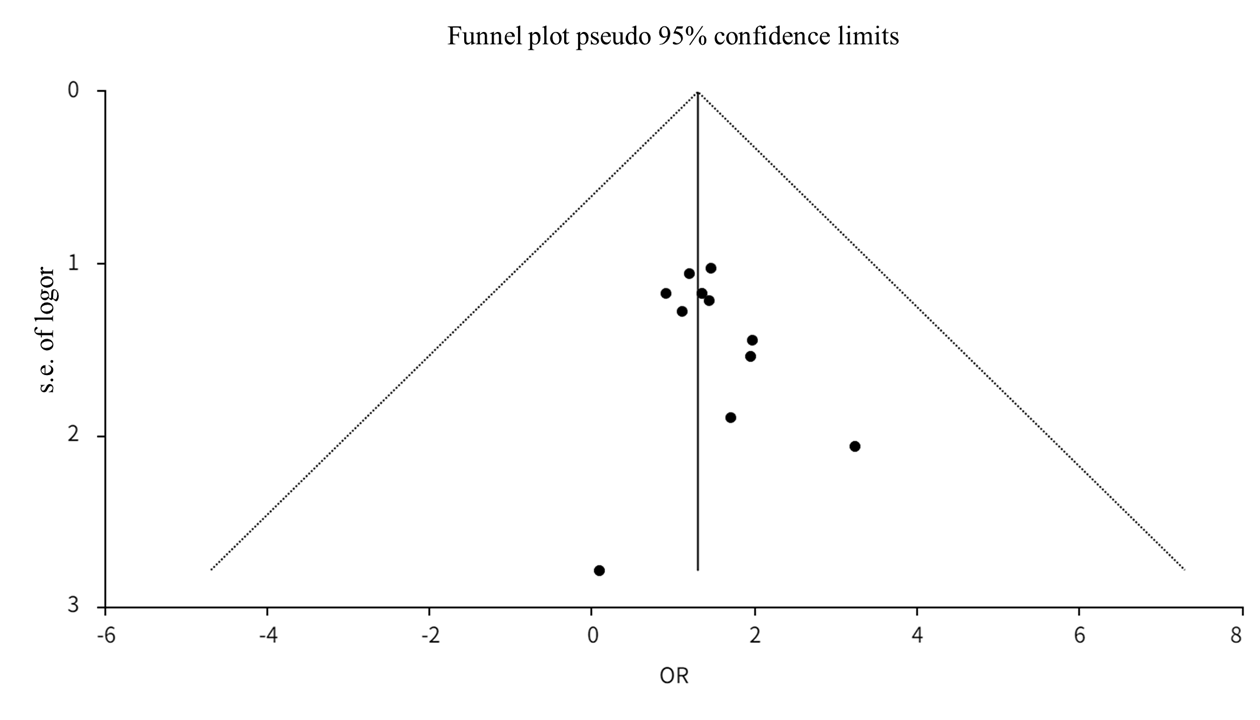


sFigure20: Funnel plot of CKD factor study.


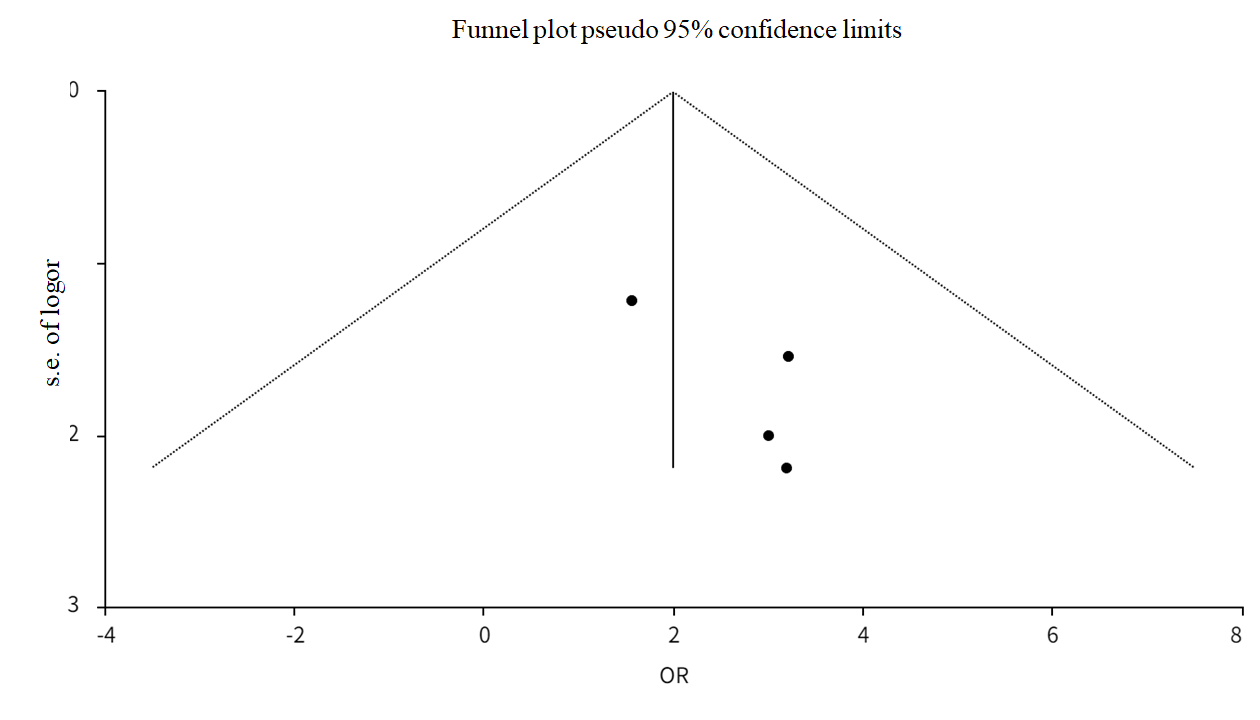


sFigure21: Funnel plot of COPD factor study.


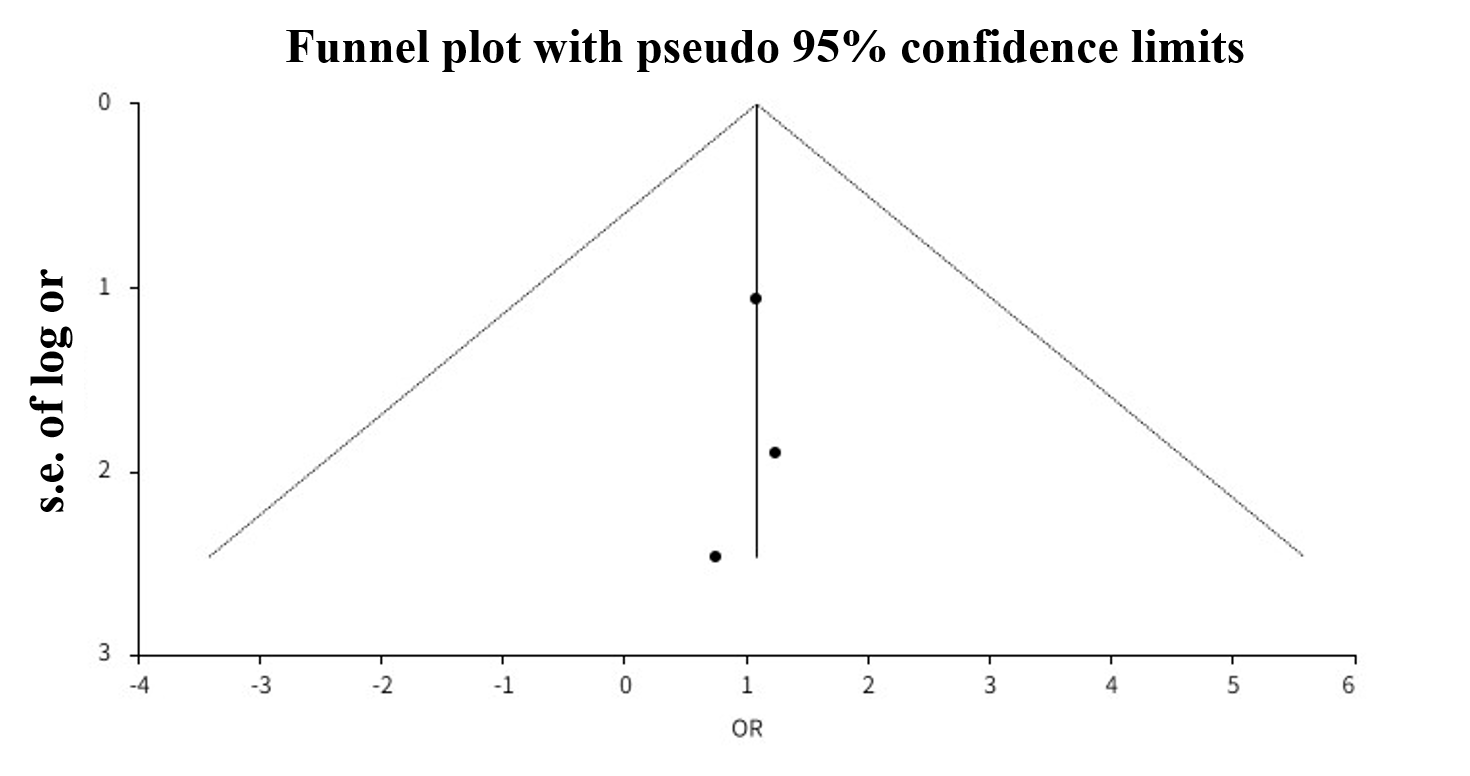


sFigure22: Meta-analysis of hypertension as a risk factor of PHN.


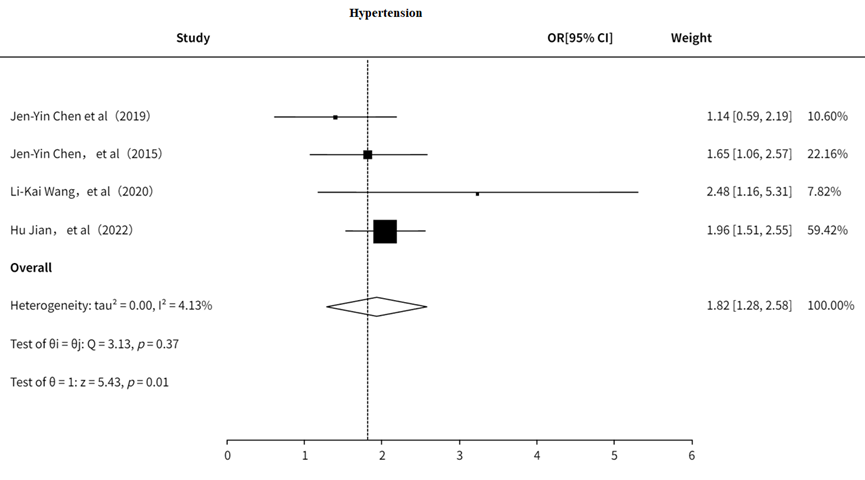


sFigure23：Funnel plot of hypertension factor study.


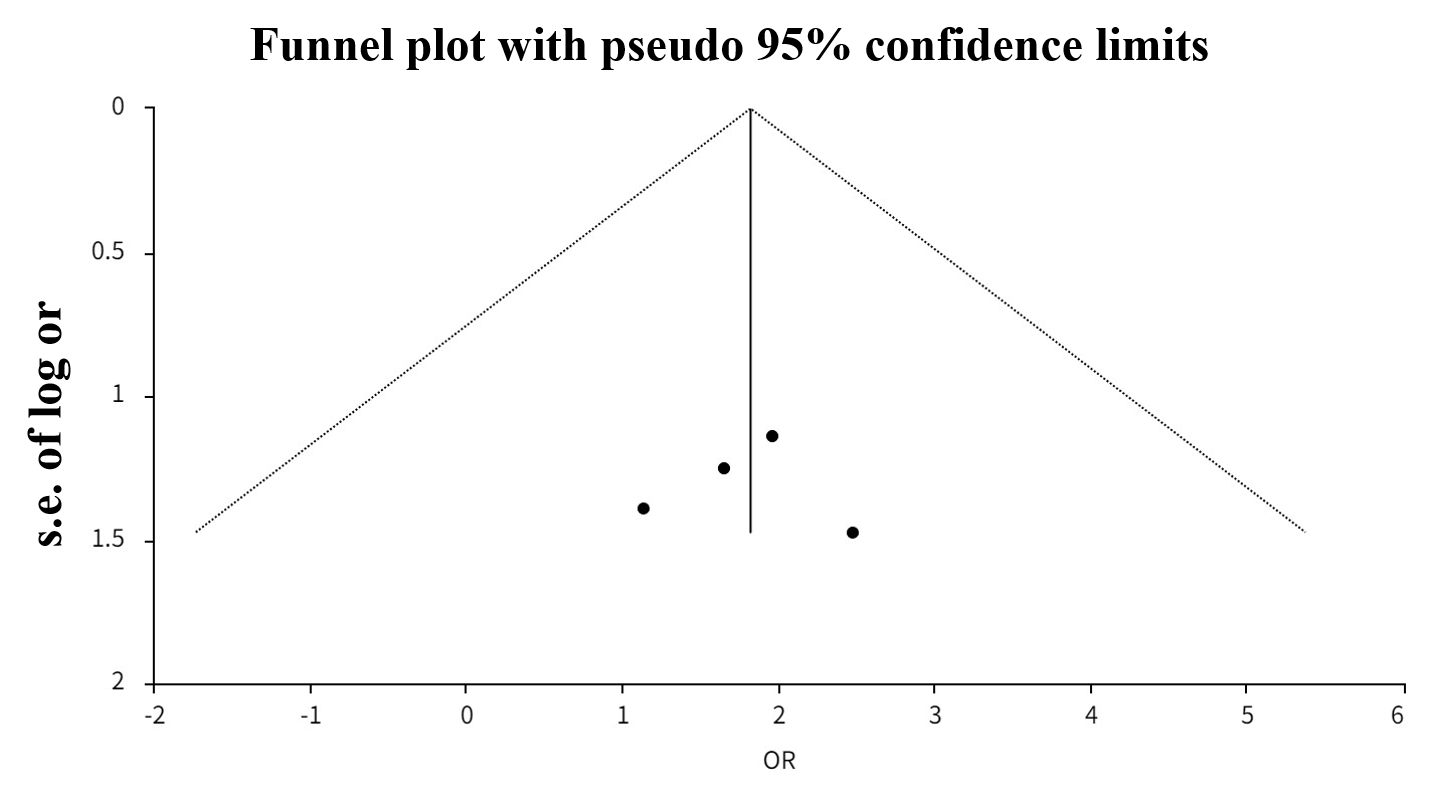

Supplement: Supplementary Figure 1 — The subgroup analyze of age as risk for PHN. [file DataSheet1.docx]
