# Supplementary material for: Risk factors for postherpetic neuralgia: a meta-analysis based on demographic, clinical features, and treatment characteristics
Source: Front Immunol. 2025 Oct 1;16:1667364. doi: 10.3389/fimmu.2025.1667364 (PMC12521459; doi:10.3389/fimmu.2025.1667364)
Supplement: Supplementary file 3 [file Table2.docx]

# Quality Assessment Report

## 1-1. Basic Information

Title: A case control study on family history as a risk factor for herpes zoster and associated outcomes, Beijing, China

Authors: Luodan Suo, Li Lu, Juan Li, et al.

Published Year: 2017

Journal: BMC Infectious Diseases

Study Design: Case-Control Study

## 2. Newcastle-Ottawa Scale (NOS) Assessment

### Selection (4/4)

| Criterion | Description | Score |
| --- | --- | --- |
| Case Definition | Clear case definition based on medical records and patient reports; reliable data from a specific geographic region (Beijing). | 1 |
| Case Representativeness | Cases were selected from urban, suburban, and rural areas in Beijing, ensuring some representativeness. | 1 |
| Control Selection | Controls were selected from the same communities/villages without a history of herpes zoster (HZ); matched by age (±5 years). | 1 |
| Control Definition | Controls were clearly defined as individuals without a history of HZ; partially self-reported, with minor recall bias. | 1 |

### Comparability (1/2)

| Criterion | Description | Score |
| --- | --- | --- |
| Adjusted Key Factors | Analysis adjusted for major confounders, including age, gender, and chronic diseases (e.g., hypertension, diabetes). | 1 |
| Adjusted Other Factors | Did not adjust for other potential factors like socioeconomic status or occupation. | 0 |

### Exposure (3/3)

| Criterion | Description | Score |
| --- | --- | --- |
| Exposure Ascertainment | Exposure (family history of HZ) was assessed through standardized questionnaires; validated through field surveys. | 1 |
| Same Method for Groups | Exposure was assessed using identical methods for both cases and controls, minimizing measurement bias. | 1 |
| Non-Response Rate | Non-response rate was not explicitly reported, but large sample size (227 cases, 678 controls) reduces potential bias. | 1 |

## 3. Total Score

Selection: 4/4

Comparability: 1/2

Exposure: 3/3

Total Score: 8/9

## 4. Quality Rating

Based on the NOS scoring system:

- High-Quality Study: Total score ≥ 6

- Moderate-Quality Study: Total score 4–5

- Low-Quality Study: Total score < 4

This study scored 8/9, indicating it is a high-quality study.

**1-2. Basic Information**

- **Title**: A Nomogram Model for Predicting Postherpetic Neuralgia in Patients with Herpes Zoster
- **Authors**: Hui-Min Hu et al.
- **Country**: China
- **Published Year**: 2024
- **Journal**: Pain Physician
- **Study Design**: Prospective Observational Study

## 2. Newcastle-Ottawa Scale (NOS) Scoring

### ****Selection (4/4)****

| **Criterion** | **Description** | **Score** |
| --- | --- | --- |
| Representativeness of the Cohort | Patients were recruited from a tertiary hospital with clear inclusion and exclusion criteria, ensuring representativeness. | 1 |
| Selection of the Non-Exposed Cohort | All included patients had Herpes Zoster but were followed to determine the development of PHN, ensuring appropriate selection. | 1 |
| Ascertainment of Exposure | Exposure (e.g., rash size, pain severity) was assessed via validated clinical data, questionnaires, and medical records. | 1 |
| Demonstration that Outcome Was Not Present at Start | PHN was not present at baseline; all participants were enrolled during the acute phase of Herpes Zoster. | 1 |

Comparability (2/2)

| **Criterion** | **Description** | **Score** |
| --- | --- | --- |

| Comparability of Cohorts | Adjusted for major confounders, including age, gender, pain severity, and rash size. | 1 |
| --- | --- | --- |

| Control for Additional Factors | Further adjusted for prodromal pain, a significant clinical predictor of PHN. | 1 |
| --- | --- | --- |

Outcome (2/3)

| Assessment of Outcome | PHN was defined as pain persisting ≥3 months after rash onset and was validated via follow-ups. | 1 |
| --- | --- | --- |

| Follow-Up Adequacy | Follow-up was conducted for all patients, but the completeness of follow-up duration was not explicitly detailed. | 1 |
| --- | --- | --- |

| Completeness of Follow-Up | Lost to follow-up rate was not reported, which may introduce potential bias. | 0 |
| --- | --- | --- |

**3. Total Score**

- **Selection**: 4/4
- **Comparability**: 2/2
- **Outcome**: 2/3
- **Total Score**: **8/9**

**1-3. Basic Information**

- **Title**: A population-based study of the epidemiology of Herpes Zoster and its complications
- **Authors**: Dahlia Weitzman, Oren Shavit, Michal Stein, Raanan Cohen, Gabriel Chodick, Varda Shalev.
- **Country**: Israel
- **Published Year**: 2013
- **Journal**: Journal of Infection
- **Study Design**: Retrospective Cohort Study

## 2. Newcastle-Ottawa Scale (NOS) Scoring

**1. Selection (4/4)**

| **Criterion** | **Description** | **Score** |
| --- | --- | --- |
| **Representativeness of the Cohort** | MHS database included a diverse population covering 25% of Israeli residents. | 1 |
| **Selection of the Non-Exposed Cohort** | Non-exposed individuals were clearly defined in the cohort. | 1 |
| **Ascertainment of Exposure** | Exposure (e.g., HZ, PHN) was ascertained via ICD-9 codes and validated with internal MHS codes. | 1 |
| **Outcome Was Not Present at Start** | HZ and PHN were newly identified during the study period, avoiding pre-existing cases. | 1 |

**2. Comparability (2/2)**

| **Criterion** | **Description** | **Score** |
| --- | --- | --- |
| **Comparability of Cohorts** | Adjusted for major confounders (age, gender, socioeconomic status, comorbidities). | 1 |
| **Control for Additional Factors** | Further adjusted for cancer history, HIV treatment, and diabetes. | 1 |

**3. Outcome (3/3)**

| **Criterion** | **Description** | **Score** |
| --- | --- | --- |
| **Assessment of Outcome** | PHN was defined based on ICD-9 codes, validated with clinical data. | 1 |
| **Follow-Up Adequacy** | Follow-up for PHN cases was comprehensive, spanning 3 months post-HZ diagnosis. | 1 |
| **Completeness of Follow-Up** | Clear documentation ensure |  |

**4.Total NOS Score**

- **Selection**: 4/4
- **Comparability**: 2/2
- **Outcome**: 3/3
- **Total Score**: **9/9**

**1-4. Basic Information**

- **Title: Analysis of Risk Factors for Postherpetic Neuralgia in Patients With Postmalignancy Herpes Zoster**
- **Authors: Hai-Li Li, Gege Gong, Jiang-Lin Wang, Fu-Bo Li**
- **Country: China**
- **Published Year: 2023**
- **Journal: Pain Physician**
- **Study Design: Retrospective Observational Study**

**2. Newcastle-Ottawa Scale (NOS) Quality Assessment**

**1. Selection (4/4)**

| **Criterion** | **Description** | **Score** |
| --- | --- | --- |
| **Representativeness of the Cohort** | **Patients were selected from a tertiary hospital and represent typical postmalignancy HZ cases in clinical practice.** | **1** |
| **Selection of the Non-Exposed Cohort** | **Non-exposed cohort (patients without PHN) was drawn from the same hospital-based HZ population.** | **1** |
| **Ascertainment of Exposure** | **Risk factors (e.g., age, NRS score, antiviral use) were extracted from hospital electronic records.** | **1** |
| **Outcome Was Not Present at Start** | **PHN was defined as pain persisting ≥3 months post-HZ onset, ensuring the outcome was not present at baseline.** | **1** |

**2. Comparability (1/2)**

| **Criterion** | **Description** | **Score** |
| --- | --- | --- |
| **Comparability of Cohorts** | **Multivariate analysis adjusted for main confounders such as acute pain severity, antiviral therapy, and tumor stage.** | **1** |
| **Control for Additional Factors** | **Other potential factors (e.g., comorbidities, sex) were not included in the multivariate model.** | **0** |

**3. Outcome (3/3)**

| **Criterion** | **Description** | **Score** |
| --- | --- | --- |
| **Assessment of Outcome** | **PHN was defined using NRS score and clinical documentation, but not independently validated.** | **1** |
| **Follow-Up Adequacy** | **Follow-up extended to at least 3 months, sufficient for PHN determination.** | **1** |
| **Completeness of Follow-Up** | **Only 4 of 74 patients were lost to follow-up (<10%), indicating acceptable cohort retention.** | **1** |

**Total NOS Score**

- **Selection: 4/4**
- **Comparability: 1/2**
- **Outcome: 3/3**
- **Total Score: 8/9**

**Quality Rating**

- **High-Quality Study (Total Score: 8/9)**

**1-5. Basic Information**

- **Title**: Analysis of the Risk Factors for Postherpetic Neuralgia
- **Authors**: Shijuan Wei, et al
- **Country**: China
- **Published Year**: 2019
- **Journal**: Dermatology
- **Study Design**: Retrospective Observational Study

**2. Newcastle-Ottawa Scale (NOS) Quality Assessment**

**1. Selection (4/4)**

| **Criterion** | **Description** | **Score** |
| --- | --- | --- |
| **Representativeness of the Cohort** | **Patients were selected from a tertiary hospital, ensuring representativeness for the study population.** | **1** |
| **Selection of the Non-Exposed Cohort** | **Non-exposed cohort (patients without PHN) was clearly defined.** | **1** |
| **Ascertainment of Exposure** | **Risk factors such as age, pain severity, and type of skin lesions were well documented in medical records.** | **1** |
| **Outcome Was Not Present at Start** | **PHN was defined as pain persisting ≥3 months, ensuring outcomes were measured post-exposure.** | **1** |

**2. Comparability (2/2)**

| **Criterion** | **Description** | **Score** |
| --- | --- | --- |
| **Comparability of Cohorts** | **Adjusted for major confounders such as age, pain severity, and antiviral therapy initiation.** | **1** |
| **Control for Additional Factors** | **Controlled for additional factors such as glucocorticoid use and affected nerves.** | **1** |

**3. Outcome (2/3)**

| **Criterion** | **Description** | **Score** |
| --- | --- | --- |
| **Assessment of Outcome** | **PHN was defined using standard clinical criteria and patient-reported data.** | **1** |
| **Follow-Up Adequacy** | **Limited follow-up details, but retrospective design ensured data completeness for all participants.** | **1** |
| **Completeness of Follow-Up** | **No explicit mention of follow-up loss, introducing potential bias.** | **0** |

**Total NOS Score**

- **Selection: 4/4**
- **Comparability: 2/2**
- **Outcome: 2/3**
- **Total Score: 8/9**

**Quality Rating**

- **High-Quality Study (Total Score: 8/9)**

**1-6. Basic Information**

- **Title**: Associations between organic erectile dysfunction and the risk of herpes zoster and postherpetic neuralgia in men
- **Authors**: Kuan-Hsun Wang, et al
- **Country**: Chian,Taiwan, 2024
- **Published Year**: 2024
- **Journal**: The Aging Male
- **Study Design**: Retrospective cohort study

**Extracted Information**

| **Field** | **Details** |
| --- | --- |
| **Author** | **Kuan-Hsun Wang, Wen-Che Hsieh, Heng-Jun Lin, Fuu-Jen Tsai, Chao-Yu Hsu** |
| **Country, Year of Publication** | **Taiwan, 2024** |
| **Journal** | **The Aging Male** |
| **Title** | **Associations between organic erectile dysfunction and the risk of herpes zoster and postherpetic neuralgia in men** |
| **Study Design** | **Retrospective cohort study** |
| **Study Population** | **Men with organic erectile dysfunction (OED) and controls without OED from Taiwan’s National Health Insurance Research Database** |
| **Study Size** | **Case group: 20,808 patients with OED; Control group: 20,808 individuals without OED** |
| **Outcome** | **Risk of herpes zoster (HZ) and postherpetic neuralgia (PHN)** |
| **Definition and Method of Ascertaining PHN** | **Pain persisting ≥3 months after rash onset; diagnosed through ICD-9 and ICD-10 codes** |
| **Method of Ascertaining Risk Factor(s)** | **Patient demographics, comorbidities, and medication usage identified through clinical records** |
| **Risk Factors Assessed** | **Age, diabetes mellitus, chronic kidney disease, coronary artery disease, depression, medication usage (e.g., beta-blockers)** |
| **Statistical Analysis** | **Cox proportional hazards models, Kaplan-Meier analysis, hazard ratios with 95% confidence intervals** |
| **NOS Score** | **8/9** |
| **Literature Quality** | **High Quality** |

**Newcastle-Ottawa Scale (NOS) Quality Assessment**

**1. Selection (4/4)**

| **Criterion** | **Description** | **Score** |
| --- | --- | --- |
| **Representativeness of the Cohort** | **Patients were selected from a large national database, ensuring representativeness.** | **1** |
| **Selection of the Non-Exposed Cohort** | **Non-exposed cohort (patients without OED) was clearly defined.** | **1** |
| **Ascertainment of Exposure** | **Risk factors were accurately extracted from clinical data.** | **1** |
| **Outcome Was Not Present at Start** | **Outcomes (HZ and PHN) were diagnosed after OED exposure.** | **1** |

**2. Comparability (2/2)**

| **Criterion** | **Description** | **Score** |
| --- | --- | --- |
| **Comparability of Cohorts** | **Controlled for key confounders, including age, comorbidities, and medications, using propensity score matching.** | **1** |
| **Control for Additional Factors** | **Additional adjustment for medication use and comorbidities.** | **1** |

**3. Outcome (2/3)**

| **Criterion** | **Description** | **Score** |
| --- | --- | --- |
| **Assessment of Outcome** | **HZ and PHN were assessed using validated ICD-9 and ICD-10 diagnostic codes.** | **1** |
| **Follow-Up Adequacy** | **Follow-up period was adequate (2000–2018).** | **1** |
| **Completeness of Follow-Up** | **No specific mention of losses to follow-up, potentially limiting the reliability of the results.** | **0** |

**Total NOS Score**

- **Selection: 4/4**
- **Comparability: 2/2**
- **Outcome: 2/3**
- **Total Score: 8/9**

**Quality Rating**

- **High-Quality Study (Total Score: 8/9)**

**1-7. Basic Information**

- **Title**: Burden of herpes zoster and postherpetic neuralgia in Japanese adults 60 years of age or older
- **Authors**: Keiko Sato, et al
- **Country**: Japan
- **Published Year**: 2017
- **Journal**: Journal of Dermatology
- **Study Design**: Prospective cohort study

**2. Newcastle-Ottawa Scale (NOS) Quality Assessment**

**1. Selection (4/4)**

| **Criterion** | **Description** | **Score** |
| --- | --- | --- |
| **Representativeness of the Cohort** | **Subjects were recruited from multiple centers, ensuring representativeness of the population aged ≥60 years.** | **1** |
| **Selection of the Non-Exposed Cohort** | **Non-exposed cohort (participants without PHN) was clearly defined.** | **1** |
| **Ascertainment of Exposure** | **Risk factors were accurately recorded using validated tools and clinical data.** | **1** |
| **Outcome Was Not Present at Start** | **PHN was defined and measured prospectively, ensuring outcomes were measured post-exposure.** | **1** |

**2. Comparability (2/2)**

| **Criterion** | **Description** | **Score** |
| --- | --- | --- |
| **Comparability of Cohorts** | **Controlled for key confounders such as age, sex, and comorbidities using multivariate analysis.** | **1** |
| **Control for Additional Factors** | **Adjusted for pain severity and rash location, which are significant predictors of PHN.** | **1** |

**3. Outcome (3/3)**

| **Criterion** | **Description** | **Score** |
| --- | --- | --- |
| **Assessment of Outcome** | **PHN was assessed using a validated pain inventory (ZBPI).** | **1** |
| **Follow-Up Adequacy** | **Follow-up duration (up to 270 days) was sufficient to capture PHN development.** | **1** |
| **Completeness of Follow-Up** | **Minimal loss to follow-up ensured reliable outcome measurements.** | **1** |

**Total NOS Score**

- **Selection: 4/4**
- **Comparability: 2/2**
- **Outcome: 3/3**
- **Total Score: 9/9**

**Quality Rating**

- **High-Quality Study (Total Score: 9/9)**

**1-8. Basic Information**

- **Title: Cerebrospinal Fluid Interleukin 8 Concentrations and the Subsequent Development of Postherpetic Neuralgia**
- **Authors: Naoki Kotani, Ryoko Kudo, Yutaka Sakurai, et al.**
- **Country: Japan**
- **Published Year: 2004**
- **Journal: The American Journal of Medicine**
- **Study Design: Prospective Observational Cohort Study**

**2. Newcastle-Ottawa Scale (NOS) Quality Assessment**

**1. Selection (4/4)**

| **Criterion** | **Description** | **Score** |
| --- | --- | --- |
| **Representativeness of the Cohort** | **Patients aged ≥50 years with acute herpes zoster were recruited from university-affiliated hospitals, reflecting a general elderly HZ population.** | **1** |
| **Selection of the Non-Exposed Cohort** | **Patients without PHN were drawn from the same cohort as the PHN group.** | **1** |
| **Ascertainment of Exposure** | **Exposure variables (e.g., cerebrospinal fluid IL-8, pain scores) were objectively measured using ELISA and standardized pain scales.** | **1** |
| **Outcome Was Not Present at Start** | **PHN (pain ≥6 months) was not present at baseline, only newly developed during follow-up.** | **0** |

**2. Comparability (1/2)**

| **Criterion** | **Description** | **Score** |
| --- | --- | --- |
| **Comparability of Cohorts** | **Adjusted for key confounders such as age, acute pain severity, and cerebrospinal fluid IL-8 concentrations.** | **1** |
| **Control for Additional Factors** | **Other variables (e.g., sex, comorbidities) were explored in univariate analysis but not included in final multivariate adjustment.** | **0** |

**3. Outcome (3/3)**

| **Criterion** | **Description** | **Score** |
| --- | --- | --- |
| **Assessment of Outcome** | **PHN was defined using a 10-cm visual analog scale and assessed at multiple follow-up points (2, 6, and 12 months).** | **1** |
| **Follow-Up Adequacy** | **Follow-up extended to 12 months, which is adequate for assessing the development of PHN.** | **1** |
| **Completeness of Follow-Up** | **All 170 patients completed the study; no dropouts were reported.** | **1** |

**Total NOS Score**

- **Selection: 3 / 4**
- **Comparability: 1 / 2**
- **Outcome: 3 / 3**
- **Total Score: 7 / 9**

**Quality Rating**

- **High-Quality Study (Total Score: 7/9)**

**1-9. Basic Information**

- **Title**: Circulating Level of Myelin Basic Protein Predicts Postherpetic Neuralgia A Prospective Study
- **Authors**: Xin Yang, et al
- **Country**: China
- **Published Year**: 2021
- **Journal**: Clinical Journal of Pain
- **Study Design**: Prospective Nested Case-Control Study

**2. Newcastle-Ottawa Scale (NOS) Quality Assessment**

**1. Selection (3/4)**

| **Criterion** | **Description** | **Score** |
| --- | --- | --- |
| **Representativeness of the Cohort** | **Patients were recruited from dermatologists in private practice, potentially limiting generalizability to the general population.** | **0** |
| **Selection of the Non-Exposed Cohort** | **Non-PHN patients (controls) were clearly defined and served as an appropriate comparison group.** | **1** |
| **Ascertainment of Exposure** | **Risk factors were documented via clinical evaluations and validated questionnaires.** | **1** |
| **Outcome Was Not Present at Start** | **PHN was measured after herpes zoster onset, ensuring it was not present at baseline.** | **1** |

**2. Comparability (2/2)**

| **Criterion** | **Description** | **Score** |
| --- | --- | --- |
| **Comparability of Cohorts** | **Adjusted for key confounders such as age, acute pain intensity, and rash extent in multivariate analyses.** | **1** |
| **Control for Additional Factors** | **Controlled for additional psychosocial factors like depression and quality of life.** | **1** |

**3. Outcome (2/3)**

| **Criterion** | **Description** | **Score** |
| --- | --- | --- |
| **Assessment of Outcome** | **PHN was assessed using VAS scores, but no external validation or independent clinical assessment was mentioned.** | **0** |
| **Follow-Up Adequacy** | **The follow-up period of 6 months was adequate to capture PHN outcomes.** | **1** |
| **Completeness of Follow-Up** | **Some patients were lost to follow-up, potentially introducing bias (not explicitly addressed).** | **0** |

**Total NOS Score**

- **Selection: 3/4**
- **Comparability: 2/2**
- **Outcome: 2/3**
- **Total Score: 7/9**

**Quality Rating**

- **Moderate-Quality Study (Total Score: 7/9)**

**1-10. Basic Information**

- **Title: Clinical and Psychosocial Correlates of Post-Herpetic Neuralgia**
- **Authors: A. Volpi, A. Gatti, F. Pica, S. Bellino, L.T. Marsella, A.F. Sabato**
- **Country: Italy**
- **Published Year: 2008**
- **Journal: Journal of Medical Virology**
- **Study Design: Prospective Observational Study**

**2. Newcastle-Ottawa Scale (NOS) Quality Assessment**

**1. Selection (3/4)**

| **Criterion** | **Description** | **Score** |
| --- | --- | --- |
| **Representativeness of the Cohort** | **Patients were enrolled by dermatologists in private practices, which may not fully reflect the general HZ population.** | **0** |
| **Selection of the Non-Exposed Cohort** | **Non-PHN patients were from the same source cohort as PHN cases.** | **1** |
| **Ascertainment of Exposure** | **Data on clinical and psychosocial risk factors were collected using physician assessment and structured tools.** | **1** |
| **Outcome Was Not Present at Start** | **PHN was clearly defined as pain ≥3 months post-rash, ensuring absence of outcome at baseline.** | **1** |

**2. Comparability (1/2)**

| **Criterion** | **Description** | **Score** |
| --- | --- | --- |
| **Comparability of Cohorts** | **Multivariate analysis adjusted for key predictors: age, acute pain intensity, rash extent, and prodromal pain.** | **1** |
| **Control for Additional Factors** | **No adjustment for other relevant confounders (e.g., sex, depression, education) in final model.** | **0** |

**3. Outcome (1/3)**

| **Criterion** | **Description** | **Score** |
| --- | --- | --- |
| **Assessment of Outcome** | **PHN diagnosis was based on self-reported pain score (≥3/10) without independent clinical confirmation.** | **0** |
| **Follow-Up Adequacy** | **A 6-month follow-up is appropriate to capture PHN cases.** | **1** |
| **Completeness of Follow-Up** | **Only 219 of 533 patients returned for follow-up; >50% lost, posing serious risk of selection bias.** | **0** |

**Total NOS Score**

- **Selection: 3/4**
- **Comparability: 1/2**
- **Outcome: 1/3**
- **Total Score: 5/9**

**Quality Rating**

- **Moderate-Quality Study (Total Score: 5/9)**

**1-11. Basic Information**

- **Title**: Correlation between Galectin-3 and Early Herpes Zoster Neuralgia and Postherpetic Neuralgia: A Retrospective Clinical Observation
- **Authors**: Tingting Wang, et al
- **Country**: China
- **Published Year**: 2020
- **Journal**: Pain Research and Management
- **Study Design**: Retrospective Observational Study

**2. Newcastle-Ottawa Scale (NOS) Quality Assessme**

**1. Selection (3/4)**

| **Criterion** | **Description** | **Score** |
| --- | --- | --- |
| **Representativeness of the Cohort** | **Patients were recruited from a single hospital, limiting generalizability to the broader population.** | **0** |
| **Selection of the Non-Exposed Cohort** | **Non-HZN neuralgia and healthy control groups were well-defined, serving as appropriate comparisons.** | **1** |
| **Ascertainment of Exposure** | **Biomarkers and clinical factors were measured using validated laboratory and clinical methods.** | **1** |
| **Outcome Was Not Present at Start** | **PHN outcomes were measured post-exposure, ensuring they were not present at baseline.** | **1** |

**2. Comparability (1/2)**

| **Criterion** | **Description** | **Score** |
| --- | --- | --- |
| **Comparability of Cohorts** | **Analysis adjusted for age and NRS scores but not comprehensively for other confounders.** | **1** |
| **Control for Additional Factors** | **Adjustment for additional variables like IL-6 and immune markers was incomplete.** | **0** |

**3. Outcome (1/3)**

| **Criterion** | **Description** | **Score** |
| --- | --- | --- |
| **Assessment of Outcome** | **PHN was assessed clinically and through NRS scores, but lacked external validation or blinding.** | **0** |
| **Follow-Up Adequacy** | **Follow-up duration of 1 month was insufficient for assessing long-term PHN outcomes.** | **0** |
| **Completeness of Follow-Up** | **No loss to follow-up was explicitly mentioned, ensuring data completeness.** | **1** |

**Corrected Total NOS Score**

- **Selection: 3/4**
- **Comparability: 1/2**
- **Outcome: 1/3**
- **Total Score: 5/9**

**Revised Quality Rating**

- **Low-Moderate Quality Study (Total Score: 5/9)**

**1-12. Basic Information**

- **Title: Efficacy of Continuous Epidural Block in Acute Herpes Zoster: Incidence and Predictive Factors of Postherpetic Neuralgia**
- **Authors: Yoo Na Kim, Dae Woo Kim, Eung Don Kim**
- **Country: South Korea**
- **Published Year: 2016**
- **Journal: Medicine**
- **Study Design: Retrospective Observational Study**

**2. Newcastle-Ottawa Scale (NOS) Quality Assessment**

**1. Selection (3/4)**

| **Criterion** | **Description** | **Score** |
| --- | --- | --- |
| **Representativeness of the Cohort** | **Patients were selected from a single tertiary hospital and all received a specific intervention (epidural block), limiting generalizability.** | **0** |
| **Selection of the Non-Exposed Cohort** | **All patients received the intervention; no traditional non-exposed (no-epidural) comparison group was included.** | **0** |
| **Ascertainment of Exposure** | **Clinical data (age, pain scores, catheterization duration) were extracted from detailed hospital medical records.** | **1** |
| **Outcome Was Not Present at Start** | **PHN (defined as pain ≥3 on NRS beyond 1, 3, or 6 months) was not present at baseline and occurred during follow-up.** | **1** |

**2. Comparability (1/2)**

| **Criterion** | **Description** | **Score** |
| --- | --- | --- |
| **Comparability of Cohorts** | **Multivariate regression adjusted for key risk factors (age, catheter duration, pain scores, diabetes, ESR).** | **1** |
| **Control for Additional Factors** | **No non-intervention group for external comparison; other demographic variables not fully controlled.** | **0** |

**3. Outcome (2/3)**

| **Criterion** | **Description** | **Score** |
| --- | --- | --- |
| **Assessment of Outcome** | **PHN defined using NRS scale and follow-up data, but not independently verified (e.g., by blinded assessors).** | **1** |
| **Follow-Up Adequacy** | **1-, 3-, and 6-month follow-up assessments were completed for all included patients.** | **1** |
| **Completeness of Follow-Up** | **63 out of 93 eligible patients were retained; ~32% were excluded due to late procedure or dropout.** | **0** |

**Total NOS Score**

- **Selection: 2 / 4**
- **Comparability: 1 / 2**
- **Outcome: 2 / 3**
- **Total Score: 5 / 9**

**Quality Rating**

- **Moderate-Quality Study (Total Score: 5/9)**

**1-13. Basic Information**

- **Title**: Herpes zoster and postherpetic neuralgia: Incidence and risk indicators using a general practice research database
- **Authors**: Wim Opstelten, et al
- **Country**: Netherlands
- **Published Year**: 2002
- **Journal**: Family Practice
- **Study Design**: Retrospective Cohort Study

**2.** **Newcastle-Ottawa Scale (NOS) Quality Assessment**

**Revised NOS Evaluation**

**1. Selection (3/4)**

| **Criterion** | **Description** | **Score** |
| --- | --- | --- |
| **Representativeness of the Cohort** | **Patients were selected from general practices, but limited to 22 practices, which might not fully represent the broader population.** | **0** |
| **Selection of the Non-Exposed Cohort** | **Non-PHN patients were clearly defined and used as an appropriate comparison group.** | **1** |
| **Ascertainment of Exposure** | **Risk factors were documented in medical records, which were considered reliable sources.** | **1** |
| **Outcome Was Not Present at Start** | **PHN was assessed after herpes zoster onset, ensuring it was not present at baseline.** | **1** |

**2. Comparability (2/2)**

| **Criterion** | **Description** | **Score** |
| --- | --- | --- |
| **Comparability of Cohorts** | **Controlled for major confounders like age, gender, and key risk factors (e.g., prodromal pain) using multivariate models.** | **1** |
| **Control for Additional Factors** | **Additional risk factors like ophthalmic localization and psychopharmaceutical use were included.** | **1** |

**3. Outcome (1/3)**

| **Criterion** | **Description** | **Score** |
| --- | --- | --- |
| **Assessment of Outcome** | **PHN was defined using patient-reported symptoms, but no external validation or blinding was performed.** | **0** |
| **Follow-Up Adequacy** | **Patients were followed for 3 months post-diagnosis, adequate to assess short-term PHN outcomes.** | **1** |
| **Completeness of Follow-Up** | **No explicit mention of follow-up losses; however, follow-up appeared complete for the study cohort.** | **1** |

**Corrected Total NOS Score**

- **Selection: 3/4**
- **Comparability: 2/2**
- **Outcome: 1/3**
- **Total Score: 6/9**

**Quality Rating**

- **Moderate-Quality Study (Total Score: 6/9)**

**1-14. Basic Information**

- **Title**: Hypovitaminosis D in Postherpetic Neuralgia—High Prevalence and Inverse Association with Pain: A Retrospective Study.
- **Authors**: Jen-Yin Chen et al.
- **Country**: China，Taiwan
- **Published Year**: 2019
- **Journal**: Taiwan
- **Study Design**: Case-control and cross-sectional

**2. Newcastle-Ottawa Scale (NOS) Quality Assessment**

**1. Selection (3/4)**

| **Criterion** | **Description** | **Score** |
| --- | --- | --- |
| **Representativeness of the Cohort** | **PHN patients were selected from a tertiary hospital in Taiwan, limiting generalizability to broader populations.** | **0** |
| **Selection of the Non-Exposed Cohort** | **Non-PHN patients (controls) were clearly defined and appropriately matched to PHN cases.** | **1** |
| **Ascertainment of Exposure** | **Serum vitamin D levels and risk factors were assessed using validated laboratory and clinical methods.** | **1** |
| **Outcome Was Not Present at Start** | **PHN was retrospectively evaluated after the onset of herpes zoster, ensuring it was not present initially.** | **1** |

**2. Comparability (2/2)**

| **Criterion** | **Description** | **Score** |
| --- | --- | --- |
| **Comparability of Cohorts** | **Adjusted for age, gender, and comorbidities using multivariate analysis.** | **1** |
| **Control for Additional Factors** | **Additional confounders, such as Helicobacter pylori infection and malignancy, were accounted for.** | **1** |

**3. Outcome (1/3)**

| **Criterion** | **Description** | **Score** |
| --- | --- | --- |
| **Assessment of Outcome** | **PHN was assessed using clinical records and questionnaires but lacked external validation or blinding.** | **0** |
| **Follow-Up Adequacy** | **PHN was assessed at ≥3 months post-onset, sufficient to evaluate short-term outcomes.** | **1** |
| **Completeness of Follow-Up** | **No explicit mention of follow-up losses, but data appeared complete for included participants.** | **1** |

**Corrected Total NOS Score**

- **Selection: 3/4**
- **Comparability: 2/2**
- **Outcome: 1/3**
- **Total Score: 6/9**

**Corrected Quality Rating**

- **Moderate-Quality Study (Total Score: 6/9)**

**1-15. Basic Information**

- **Title: Impact of Postherpetic Neuralgia: A Six-Year Population-Based Analysis on People Aged 50 Years or Older**
- **Authors: Cintia Muñoz-Quiles, Mónica López-Lacort, Alejandro Orrico-Sánchez, Javier Díez-Domingo**
- **Country: Spain**
- **Published Year: 2018**
- **Journal: Journal of Infection**
- **Study Design: Retrospective Population-Based Cohort Study**

**2. Newcastle-Ottawa Scale (NOS) Quality Assessment**

**1. Selection (3/4)**

| **Criterion** | **Description** | **Score** |
| --- | --- | --- |
| **Representativeness of the Cohort** | **Individuals aged ≥50 years were selected from the Valencia Region health registry. However, patients with immunosuppression were excluded, limiting full population representativeness.** | **0** |
| **Selection of the Non-Exposed Cohort** | **Herpes zoster patients who did not develop PHN were drawn from the same registry and follow-up period.** | **1** |
| **Ascertainment of Exposure** | **Exposure to HZ and comorbidities was assessed through ICD-9-CM codes and validated health system databases.** | **1** |
| **Outcome Was Not Present at Start** | **PHN was defined as persistent pain ≥90 days post-HZ diagnosis; outcome occurred after cohort entry.** | **1** |

**2. Comparability (2/2)**

| **Criterion** | **Description** | **Score** |
| --- | --- | --- |
| **Comparability of Cohorts** | **Multivariate Poisson regression adjusted for age, sex, year, diabetes, COPD, HF, and health department effects.** | **1** |
| **Control for Additional Factors** | **Included socioeconomic and geographical covariates (urban/rural, exclusion risk) enhancing internal validity.** | **1** |

**3. Outcome (2/3)**

| **Criterion** | **Description** | **Score** |
| --- | --- | --- |
| **Assessment of Outcome** | **PHN was identified using diagnostic codes, analgesic prescriptions, and chronic pain codes. No independent clinical validation was conducted.** | **1** |
| **Follow-Up Was Long Enough** | **Patients were followed for up to 6 years, sufficient to observe PHN development and outcomes.** | **1** |
| **Completeness of Follow-Up** | **The study excluded immunosuppressed patients and had no reporting on loss to follow-up. Thus, potential for untracked attrition exists.** | **0** |

**Total NOS Score**

- **Selection: 3 / 4**
- **Comparability: 2 / 2**
- **Outcome: 2 / 3**
- **Total Score: 7 / 9**

**Quality Rating**

- **Moderate-to-High Quality Study (Total Score: 7/9)**

**1-16. Basic Information**

- **Title: Incidence and costs of herpes zoster and postherpetic neuralgia in German adults aged ≥50 years: A prospective study**
- **Authors: Ruprecht Schmidt-Ott, e tal**
- **Country: Germany**
- **Published Year: 2018**
- **Journal: Journal of Infection**
- **Study Design: Retrospective Population-Based Cohort Study**

**2. Newcastle-Ottawa Scale (NOS) Quality Assessment**

| **Criterion** | | **Description** | | **Score** | |  |  |
| --- | --- | --- | --- | --- | --- | --- | --- |
| **Representativeness of the Cohort** | | **Patients were recruited from a single hospital, which limits generalizability to the broader transplant population.** | | **0** | |  |  |
| **Selection of the Non-Exposed Cohort** | | **Non-HZ patients were clearly defined and served as appropriate controls for comparison.** | | **1** | |  |  |
| **Ascertainment of Exposure** | | **Risk factors and outcomes were documented through validated medical records, ensuring accuracy.** | | **1** | |  |  |
| **Outcome Was Not Present at Start** | | **PHN was assessed post-HZ onset, ensuring it was not present at baseline.** | | **1** | |  |  |
| **Domain** | | **Criterion** | | **Description** | | **Score** | |
| **Selection** | | **Representativeness of the Cohort** | | **Patients were recruited from a single hospital, which limits generalizability to the broader transplant population.** | | **0** | |
| **Selection** | | **Selection of the Non-Exposed Cohort** | | **Non-HZ patients were clearly defined and served as appropriate controls for comparison.** | | **1** | |
| **Selection** | | **Ascertainment of Exposure** | | **Risk factors and outcomes were documented through validated medical records, ensuring accuracy.** | | **1** | |
| **Selection** | | **Outcome Was Not Present at Start** | | **PHN was assessed post-HZ onset, ensuring it was not present at baseline.** | | **1** | |
| **Comparability** | | **Comparability of Cohorts** | | **Controlled for age and gender but did not adjust for all critical confounders (e.g., pre-transplant VZV history).** | | **1** | |
| **Comparability** | | **Control for Additional Factors** | | **Did not adjust for certain immunological factors beyond basic demographics and comorbidities.** | | **0** | |
| **Outcome** | | **Assessment of Outcome** | | **PHN diagnosis relied on clinical evaluations but lacked external validation or blinding.** | | **0** | |
| **Outcome** | | **Follow-Up Adequacy** | | **Median follow-up of 38 months was sufficient to assess PHN outcomes.** | | **1** | |
| **Outcome** | | **Completeness of Follow-Up** | | **No explicit mention of follow-up losses, but the reported data appears complete for all patients.** | | **1** | |

**Corrected Total NOS Score**

- **Selection: 2 / 4**
- **Comparability: 1 / 2**
- **Outcome: 1 / 3**
- **Total Score: 4 / 9**

**Corrected Quality Rating**

- **Low–Moderate Quality Study (Total Score: 4 / 9)**

**1-17. Basic Information**

- **Title**: Incidences of Herpes Zoster and Postherpetic Neuralgia in Japanese Adults Aged 50 Years and Older From a Community-based Prospective Cohort Study: The SHEZ Study..
- **Authors**: Ioanna D. Pavlopoulou, et al.
- **Country**: Germany
- **Published Year**: 2018
- **Journal**: Journal of Infection
- **Study Design**: Prospective Observational Cohort Study

**2. Newcastle-Ottawa Scale (NOS) Quality Assessment**

**1. Selection (2/4)**

| **Criterion** | **Description** | **Score** |
| --- | --- | --- |
| **Representativeness of the Cohort** | **Patients were recruited from a single hospital, which limits generalizability to the broader transplant population.** | **0** |
| **Selection of the Non-Exposed Cohort** | **Non-HZ patients were clearly defined and served as appropriate controls for comparison.** | **1** |
| **Ascertainment of Exposure** | **Risk factors and outcomes were documented through validated medical records, ensuring accuracy.** | **1** |
| **Outcome Was Not Present at Start** | **PHN was assessed post-HZ onset, ensuring it was not present at baseline.** | **1** |

**2. Comparability (1/2)**

| **Criterion** | **Description** | **Score** |
| --- | --- | --- |
| **Comparability of Cohorts** | **Controlled for age and gender but did not adjust for all critical confounders (e.g., pre-transplant VZV history).** | **1** |
| **Control for Additional Factors** | **Did not adjust for certain immunological factors beyond basic demographics and comorbidities.** | **0** |

**3. Outcome (1/3)**

| **Criterion** | **Description** | **Score** |
| --- | --- | --- |
| **Assessment of Outcome** | **PHN diagnosis relied on clinical evaluations but lacked external validation or blinding.** | **0** |
| **Follow-Up Adequacy** | **Median follow-up of 38 months was sufficient to assess PHN outcomes.** | **1** |
| **Completeness of Follow-Up** | **No explicit mention of follow-up losses, but the reported data appears complete for all patients.** | **1** |

**Corrected Total NOS Score**

- **Selection: 2/4**
- **Comparability: 1/2**
- **Outcome: 1/3**
- **Total Score: 4/9**

**Corrected Quality Rating**

- **Low-Moderate Quality Study (Total Score: 4/9)**

**1-18. Basic Information**

- **Title: Increased Incidence of Herpes Zoster and Postherpetic Neuralgia in Adult Patients following Traumatic Brain Injury: A Nationwide Population-Based Study in Taiwan**
- **Authors: Yi-Ching Tung, Hung-Pin Tu, Wen-Chan Tsai, Cheng-Sheng Chen, Chen-Hsiang Su, Hon-Yi Shi, Chih-Lung Lin**
- **Country: Taiwan**
- **Published Year: 2015**
- **Journal: PLOS ONE**
- **Study Design: Retrospective Population-Based Cohort Study**

**2. Newcastle-Ottawa Scale (NOS) Quality Assessment**

**1. Selection (3/4)**

| **Criterion** | **Description** | **Score** |
| --- | --- | --- |
| **Representativeness of the Cohort** | **TBI patients were identified from the Taiwan NHIRD, which is representative of the general population. However, very mild TBI patients were excluded.** | **1** |
| **Selection of the Non-Exposed Cohort** | **Controls were randomly selected from the same national database and matched by age, sex, and index year.** | **1** |
| **Ascertainment of Exposure** | **TBI diagnosis was based on ICD-9 codes from hospitalization data. Accuracy of coding was not independently validated.** | **1** |
| **Outcome Was Not Present at Start** | **Individuals with prior herpes zoster were excluded, ensuring PHN was not present at cohort entry.** | **1** |

**2. Comparability (1/2)**

| **Criterion** | **Description** | **Score** |
| --- | --- | --- |
| **Comparability of Cohorts** | **The analysis controlled for age, sex, and Charlson Comorbidity Index in multivariable Cox models.** | **1** |
| **Control for Additional Factors** | **No psychosocial, behavioral, or nutritional covariates were included despite their known relevance to PHN risk.** | **0** |

**3. Outcome (1/3)**

| **Criterion** | **Description** | **Score** |
| --- | --- | --- |
| **Assessment of Outcome** | **PHN was identified solely using ICD-9 diagnostic codes without clinical verification or standardized pain assessment.** | **0** |
| **Follow-Up Was Long Enough** | **Patients were followed up for 15 years, which is more than sufficient for PHN detection.** | **1** |
| **Completeness of Follow-Up** | **The NHIRD provides continuous tracking through insurance coverage; however, the study did not explicitly report on loss to follow-up.** | **0** |

**Total NOS Score**

- **Selection: 4 / 4**
- **Comparability: 1 / 2**
- **Outcome: 1 / 3**
- **Total Score: 6 / 9**

**Quality Rating**

- **Moderate-Quality Study (Total Score: 6/9)**

**1-19. Basic Information**

- **Title**: Nutrient Deficiencies as a Risk Factor in Taiwanese Patients with Postherpetic Neuralgia
- **Authors**: Jen-Yin Chen, et al
- **Country**: China，Taiwan
- **Published Year**: 2011
- **Journal**: British Journal of Nutrition
- **Study Design**: Cross-Sectional Case-Control Study

**2.**

**Newcastle-Ottawa Scale (NOS) Quality Assessment**

**1. Selection (3/4)**

| **Criterion** | **Description** | **Score** |
| --- | --- | --- |
| **Representativeness of the Cases** | **Cases were drawn from a single hospital population, which limits generalizability.** | **0** |
| **Selection of Controls** | **Controls were matched to cases based on age and gender, recruited from the same population.** | **1** |
| **Definition of Cases** | **PHN was defined using clear clinical criteria based on pain score and duration.** | **1** |
| **Controls Had No History of Outcome** | **Controls were confirmed to have no history of PHN or herpes zoster.** | **1** |

**2. Comparability (1/2)**

| **Criterion** | **Description** | **Score** |
| --- | --- | --- |
| **Comparability of Cases and Controls** | **Controlled for age and gender but did not adjust for other confounders such as comorbidities.** | **1** |
| **Additional Adjustments** | **No additional adjustments for potential confounders beyond demographics.** | **0** |

**3. Exposure (2/3)**

| **Criterion** | **Description** | **Score** |
| --- | --- | --- |
| **Ascertainment of Exposure** | **Plasma and serum nutrient levels were measured using validated laboratory methods.** | **1** |
| **Same Method of Ascertainment for Both Groups** | **Identical procedures for nutrient measurement were applied to cases and controls.** | **1** |
| **Non-Response Rate** | **Non-response rate was not reported or accounted for in the study.** | **0** |

**Total NOS Score**

- **Selection: 3/4**
- **Comparability: 1/2**
- **Exposure: 2/3**
- **Total Score: 6/9**

**Quality Rating**

- **Moderate Quality Study (Total Score: 6/9)**

**1-20. Basic Information**

- **Title**: Peptic Ulcer as a Risk Factor for Postherpetic Neuralgia in Adult Patients with Herpes Zoster
- **Authors**: Jen-Yin Chen, Kuo-Mao Lan, Ming-Jen Sheu, Su-Feng Tseng, Shih-Feng Weng, Miao-Lin Hu
- **Country**: China，Taiwan
- **Published Year**: 2015
- **Journal**: Journal of Medical Virology
- **Study Design**: Retrospective Case-Control Study

**2.**

**OS Scoring**

**1. Selection (2/4)**

**Criterion Description Score**

**Representativeness of the Cases Cases were selected from a single hospital, limiting generalizability to the broader population. 0**

**Selection of Controls Controls were selected from the same population as the cases, matched based on inclusion/exclusion criteria. 1**

**Definition of Cases PHN was clearly defined using ICD-9 codes and clinical review criteria (≥90 days of persistent pain). 1**

**Controls Had No History of Outcome Controls had no history of PHN, confirmed via medical records and ICD-9 codes. 1**

**2. Comparability (1/2)**

**Criterion Description Score**

**Comparability of Cases and Controls Adjustments for age and acute herpetic pain were made in the statistical models. 1**

**Additional Adjustments No additional adjustments were made for confounders like comorbidities (e.g., H. pylori, NSAID use). 0**

**3. Exposure (2/3)**

**Criterion Description Score**

**Ascertainment of Exposure Exposure (e.g., peptic ulcer, H. pylori infection) was assessed using validated medical records. 1**

**Same Method of Ascertainment for Both Groups Identical methods were used to assess exposure in both cases and controls. 1**

**Non-Response Rate Non-response or incomplete record rates were not fully addressed, potentially introducing bias. 0**

**Corrected Total NOS Score**

**Selection: 2/4**

**Comparability: 1/2**

**Exposure: 2/3**

**Total Score: 5/9**

**Corrected Quality Rating**

**Moderate-Quality Study (Total Score: 5/9)**

**1-21. Basic Information**

- **Title**: Plasma Vitamin C Concentrations Were Negatively Associated with Tingling, Prickling, or Pins and Needles Sensation in Patients with Postherpetic Neuralgia
- **Authors**: Li-Kai Wang, Yao-Tsung Lin, Kuo-Chuan Hung, Chia-Yu Chang, Zhi-Fu Wu, Miao-Lin Hu, Jen-Yin Chen
- **Country**: China，Taiwan
- **Published Year**: 2020
- **Journal**: Nutrients
- **Study Design**: Cross-Sectional Study

**Newcastle-Ottawa Scale (NOS) Quality Assessment**

**1. Selection (3/4)**

| **Criterion** | **Description** | **Score** |
| --- | --- | --- |
| **Representativeness of the Cases** | **Cases were recruited from a single center (Chi Mei Medical Center), limiting generalizability.** | **0** |
| **Selection of Controls** | **Not applicable (cross-sectional design, no control group).** | **0** |
| **Definition of Cases** | **Clear definition of PHN using clinical criteria and the LANSS questionnaire.** | **1** |
| **Controls Had No History of Outcome** | **Not applicable.** | **0** |

**2. Comparability (1/2)**

| **Criterion** | **Description** | **Score** |
| --- | --- | --- |
| **Comparability of Cases and Controls** | **Adjustments made for key confounders (smoking, dietary intake, comorbidities) in multivariate analysis.** | **1** |
| **Additional Adjustments** | **Did not adjust for other potential confounders like socioeconomic status or physical activity.** | **0** |

**3. Exposure (2/3)**

| **Criterion** | **Description** | **Score** |
| --- | --- | --- |
| **Ascertainment of Exposure** | **Plasma vitamin C levels measured using validated laboratory methods.** | **1** |
| **Same Method of Ascertainment for Both Groups** | **Not applicable (single group study).** | **0** |
| **Non-Response Rate** | **No non-response or attrition issues reported.** | **1** |

**Corrected Total NOS Score**

- **Selection: 1/4**
- **Comparability: 1/2**
- **Exposure: 2/3**
- **Total Score: 4/9**

**Quality Rating**

- **Moderate Quality Study (Total Score: 4/9)**

**1-22. Basic Information**

- **Title**: Predicting postherpetic neuralgia in elderly primary care patients with herpes zoster: Prospective prognostic study.
- **Authors**: Wim Opstelten, et al
- **Country**: Netherlands
- **Published Year**: 2007
- **Journal**: Pain
- **Study Design**: Prospective Cohort Study

**2. NOS Scoring**

**1. Selection (4/4)**

| **Criterion** | **Description** | **Score** |
| --- | --- | --- |
| **Representativeness of the Cohort** | **Participants were recruited from elderly primary care patients, broadly representative of this population.** | **1** |
| **Selection of the Non-Exposed Cohort** | **Controls were appropriately selected from non-PHN patients within the cohort.** | **1** |
| **Ascertainment of Exposure** | **Exposure (e.g., rash severity, acute pain) was measured using validated clinical tools.** | **1** |
| **Outcome Was Not Present at Start** | **PHN was assessed three months after inclusion, ensuring it was not present at baseline.** | **1** |

**2. Comparability (2/2)**

| **Criterion** | **Description** | **Score** |
| --- | --- | --- |
| **Comparability of Cohorts** | **Adjusted for key confounders such as age, rash severity, and acute pain severity.** | **1** |
| **Additional Adjustments** | **Psychological predictors and serological parameters were considered as additional confounders.** | **1** |

**3. Outcome (2/3)**

| **Criterion** | **Description** | **Score** |
| --- | --- | --- |
| **Assessment of Outcome** | **PHN was defined and measured using VAS questionnaires, but no external validation or blinding was conducted.** | **0** |
| **Follow-Up Adequacy** | **Three months was sufficient for assessing PHN outcomes, given the standard definition of PHN.** | **1** |
| **Completeness of Follow-Up** | **No significant loss to follow-up was reported.** | **1** |

**Corrected Total NOS Score**

- **Selection: 4/4**
- **Comparability: 2/2**
- **Outcome: 2/3**
- **Total Score: 8/9**

**Corrected Quality Rating**

- **High-Quality Study (Total Score: 8/9)**

**1-23. Basic Information**

- **Title**: Postherpetic neuralgia is associated with an increased risk of coronary heart disease: A population-based cohort study.
- **Authors**: Pei-Shan Tsai, et al
- **Country**: Chian，Taiwan
- **Published Year**: 2014
- **Journal**: International Journal of Cardiology
- **Study Design**: Retrospective, population-based matched cohort using Taiwan’s Longitudinal Health Insurance Database (2000)
  1. **Newcastle–Ottawa Scale (NOS) Quality Assessment**

**Selection (4/4)**

| **Criterion** | **Description** | **Score** |
| --- | --- | --- |
| **Representativeness of the Cohort** | **Population-based sample from national insurance registry covering 1 million beneficiaries; minimal selection bias.** | **1** |
| **Selection of the Non-Exposed Cohort** | **Age- and sex-matched non-PHN controls drawn from the same registry in a 1:3 ratio.** | **1** |
| **Ascertainment of Exposure** | **PHN diagnosed via validated ICD-9-CM codes in a comprehensive health-system database.** | **1** |
| **Outcome Not Present at Start** | **Excluded subjects with prior CHD events; PHN onset defines cohort entry.** | **1** |

1. **Comparability (2/2)**

| **Criterion** | **Description** | **Score** |
| --- | --- | --- |
| **Control for Confounders** | **Adjusted for demographic and clinical risk factors (age, sex, comorbidities, medication use, healthcare utilization).** | **1** |
| **Additional Adjustment** | **Further adjustment by propensity score to balance unmeasured confounders between PHN and non-PHN cohorts.** | **1** |

1. **Outcome (3/3)**

| **Criterion** | **Description** | **Score** |
| --- | --- | --- |
| **Assessment of Outcome** | **CHD events identified objectively via ICD-9‐CM diagnostic and procedure codes (MI, PCI, CABG).** | **1** |
| **Follow-up Long Enough** | **Minimum 1-year follow-up is sufficient to capture incident CHD events after PHN onset.** | **1** |
| **Adequacy of Follow-up of Cohorts** | **Use of administrative claims data ensures near-complete follow-up; loss to follow-up negligible.** | **1** |

**Corrected Total NOS Score**

- **Selection: 4 / 4**
- **Comparability: 2 / 2**
- **Outcome: 3 / 3**
- **Total Score: 9 / 9**

**Quality Rating**

- **High-quality cohort study (Total Score: 9 / 9)**

**1-24. Basic Information**

- **Title**: Predictors of pain intensity and persistence in a prospective Italian cohort of patients with herpes zoster: relevance of smoking, trauma, and antiviral therapy.
- **Authors**: Giustino Parruti, et al.
- **Country**: Italy
- **Published Year**: 2010
- **Journal**: BMC Medicine
- **Study Design**: Prospective Cohort Study

**2. Newcastle-Ottawa Scale (NOS) Quality Assessment**

**1. Selection (4/4)**

| **Criterion** | **Description** | **Score** |
| --- | --- | --- |
| **Representativeness of the Cohort** | **Patients were recruited from general practitioners and hospitals, ensuring a broad and representative cohort.** | **1** |
| **Selection of the Non-Exposed Cohort** | **Non-PHN patients were included as a comparison group.** | **1** |
| **Ascertainment of Exposure** | **Exposure (e.g., trauma, smoking) was assessed using validated methods (interviews and medical records).** | **1** |
| **Outcome Was Not Present at Start** | **PHN was not present at baseline as it was assessed during follow-up.** | **1** |

**2. Comparability (2/2)**

| **Criterion** | **Description** | **Score** |
| --- | --- | --- |
| **Comparability of Cohorts** | **Adjusted for major confounders such as age, pain at presentation, and antiviral use.** | **1** |
| **Control for Additional Factors** | **Additional factors, such as smoking and trauma, were included in multivariable analyses.** | **1** |

**3. Outcome (2/3)**

| **Criterion** | **Description** | **Score** |
| --- | --- | --- |
| **Assessment of Outcome** | **Pain and PHN were assessed using a semi-quantitative scale, but no external validation was performed.** | **0** |
| **Follow-Up Adequacy** | **Follow-up was conducted for up to 12 months, which is adequate for assessing PHN.** | **1** |
| **Completeness of Follow-Up** | **Minimal loss to follow-up was reported, with 441 patients completing the study.** | **1** |

**Total NOS Score**

- **Selection: 4/4**
- **Comparability: 2/2**
- **Outcome: 2/3**
- **Total Score: 8/9**

**Quality Rating**

- **High-Quality Study (Total Score: 8/9)**

**1-25. Basic Information**

- **Title**: Predictors of Postherpetic Neuralgia Among Patients With Herpes Zoster: A Prospective Study.
- **Authors**: Mélanie Drolet, et al.
- **Country**: Canada
- **Published Year**: 2010
- **Journal**: The Journal of Pain
- **Study Design**: Prospective Cohort Study

**2.**

**Corrected NOS Scoring**

**1. Selection (4/4)**

| **Criterion** | **Description** | **Score** |
| --- | --- | --- |
| **Representativeness of the Cohort** | **Patients were recruited from diverse Canadian clinics, ensuring a representative sample of HZ cases.** | **1** |
| **Selection of the Non-Exposed Cohort** | **Non-PHN cases were appropriately included within the cohort.** | **1** |
| **Ascertainment of Exposure** | **Exposure factors (e.g., age, acute pain severity) were documented using validated methods.** | **1** |
| **Outcome Was Not Present at Start** | **PHN was defined and assessed after ≥90 days, ensuring it was not present at baseline.** | **1** |

**2. Comparability (2/2)**

| **Criterion** | **Description** | **Score** |
| --- | --- | --- |
| **Comparability of Cohorts** | **Adjusted for major confounders such as age, pain severity, and functional limitations.** | **1** |
| **Control for Additional Factors** | **Multivariable regression models accounted for additional potential confounders (e.g., antiviral use).** | **1** |

**3. Outcome (2/3)**

| **Criterion** | **Description** | **Score** |
| --- | --- | --- |
| **Assessment of Outcome** | **PHN was measured using validated clinical tools and questionnaires, but there was no external validation or blinding.** | **0** |
| **Follow-Up Adequacy** | **Follow-up lasted up to 180 days, sufficient to assess PHN outcomes.** | **1** |
| **Completeness of Follow-Up** | **Minimal loss to follow-up was reported; 89-98% completed questionnaires at each follow-up point.** | **1** |

**Corrected Total NOS Score**

- **Selection: 4/4**
- **Comparability: 2/2**
- **Outcome: 2/3**
- **Total Score: 8/9**

**Corrected Quality Rating**

- **High Quality Study (Total Score: 8/9)**

**1-26. Basic Information**

- **Title: Predictors of Postherpetic Neuralgia in Patients With Herpes Zoster: A Pooled Analysis of Prospective Cohort Studies from North and Latin America and Asia**
- **Authors: Kosuke Kawai, Emmanouil Rampakakis, Tsen-Fang Tsai, et al.**
- **Country: Multinational (Canada, Brazil, Mexico, Argentina, Taiwan, South Korea, Thailand)**
- **Published Year: 2015**
- **Journal: International Journal of Infectious Diseases**
- **Study Design: Pooled Prospective Cohort Study**

**2. Newcastle-Ottawa Scale (NOS) Quality Assessment**

**1. Selection (3/4)**

| **Criterion** | **Description** | **Score** |
| --- | --- | --- |
| **Representativeness of the Cohort** | **Patients aged ≥50 with confirmed HZ rash were enrolled from multiple countries and settings; however, convenience sampling and site-specific differences limit full population representativeness.** | **0** |
| **Selection of the Non-Exposed Cohort** | **Non-PHN patients came from the same cohort, recruited using identical protocols and follow-up schedules.** | **1** |
| **Ascertainment of Exposure** | **Exposure factors such as acute pain severity and functional status were assessed using validated tools (ZBPI, EQ-5D).** | **1** |
| **Outcome Was Not Present at Start** | **PHN (pain score ≥3 after 90 days) was not present at baseline; enrollment was within 14 days of HZ rash onset.** | **1** |

**2. Comparability (1/2)**

| **Criterion** | **Description** | **Score** |
| --- | --- | --- |
| **Comparability of Cohorts** | **Multivariate regression controlled for age, acute pain, walking problems, and social interference.** | **1** |
| **Control for Additional Factors** | **Other important variables (e.g., sex, antiviral timing, comorbidities, psychological distress) were not adjusted in the final model.** | **0** |

**3. Outcome (2/3)**

| **Criterion** | **Description** | **Score** |
| --- | --- | --- |
| **Assessment of Outcome** | **PHN was based on self-reported ZBPI pain score (≥3) after 90 days, without clinical or objective confirmation.** | **0** |
| **Follow-Up Was Long Enough** | **Patients were followed for at least 6 months, sufficient for PHN onset detection.** | **1** |
| **Completeness of Follow-Up** | **No major loss to follow-up reported; follow-up compliance was implied by outcome data.** | **1** |

**Total NOS Score**

- **Selection: 3 / 4**
- **Comparability: 1 / 2**
- **Outcome: 2 / 3**
- **Total Score: 6 / 9**

**Quality Rating**

- **Moderate-Quality Study (Total Score: 6/9)**

**1-27. Basic Information**

- **Title**: Psychosocial Risk Factors for Postherpetic Neuralgia: A Prospective Study of Patients With Herpes Zost.
- **Authors**: Jennifer Katz,et al
- **Country**: USA
- **Published Year**: 2005
- **Journal**: The Journal of Pain
- **Study Design**: Prospective Cohort Study

**2. NOS Scoring**

**1. Selection (2/4)**

| **Criterion** | **Description** | **Score** |
| --- | --- | --- |
| **Representativeness of the Cohort** | **Patients were recruited from dermatology clinics and community physicians, but the sample size (n=102) was relatively small, limiting generalizability.** | **0** |
| **Selection of the Non-Exposed Cohort** | **Non-PHN patients were appropriately included within the cohort for comparison.** | **1** |
| **Ascertainment of Exposure** | **Exposure factors (e.g., pain severity, psychosocial measures) were assessed using validated tools.** | **1** |
| **Outcome Was Not Present at Start** | **PHN was defined and assessed prospectively after ≥4 months, ensuring it was not present at baseline.** | **1** |

**2. Comparability (2/2)**

| **Criterion** | **Description** | **Score** |
| --- | --- | --- |
| **Comparability of Cohorts** | **Adjusted for key confounders such as age, acute pain severity, and psychosocial factors.** | **1** |
| **Control for Additional Factors** | **Personality disorder symptoms and life stressors were considered as additional confounders.** | **1** |

**3. Outcome (2/3)**

| **Criterion** | **Description** | **Score** |
| --- | --- | --- |
| **Assessment of Outcome** | **PHN was assessed using validated questionnaires, but no external validation or blinding was conducted.** | **0** |
| **Follow-Up Adequacy** | **Follow-up duration of 3.5 to 12 months was sufficient to assess PHN outcomes.** | **1** |
| **Completeness of Follow-Up** | **Minimal loss to follow-up; 102 out of 110 participants completed follow-up.** | **1** |

**Corrected Total NOS Score**

- **Selection: 2/4**
- **Comparability: 2/2**
- **Outcome: 2/3**
- **Total Score: 6/9**

**Corrected Quality Rating**

- **Moderate-Quality Study (Total Score: 6/9)**

**1-28. Basic Information**

- **Title**: Quantification of Risk Factors for Postherpetic Neuralgia in Herpes Zoster Patients: A Cohort Study
- **Authors**: Harriet J. Forbes,et al,
- **Country**: United Kingdom
- **Published Year**: 2016
- **Journal**: Neurology
- **Study Design**: Retrospective Cohort Study

**2. NOS Scoring**

**1. Selection (3/4)**

| **Criterion** | **Description** | **Score** |
| --- | --- | --- |
| **Representativeness of the Cohort** | **Patients were recruited from a large, representative database, but the cohort is limited to primary care data, potentially excluding hospitalized cases.** | **0** |
| **Selection of the Non-Exposed Cohort** | **Non-PHN patients were appropriately included within the cohort for comparison.** | **1** |
| **Ascertainment of Exposure** | **Exposure factors were reliably assessed using standardized electronic health records.** | **1** |
| **Outcome Was Not Present at Start** | **PHN was defined as pain persisting ≥90 days after herpes zoster diagnosis, ensuring it was not present at baseline.** | **1** |

**2. Comparability (2/2)**

| **Criterion** | **Description** | **Score** |
| --- | --- | --- |
| **Comparability of Cohorts** | **Adjusted for key confounders such as age, sex, and severe immunosuppression.** | **1** |
| **Control for Additional Factors** | **Adjustments were made for comorbidities, BMI, and antiviral use, accounting for additional confounders.** | **1** |

**3. Outcome (2/3)**

| **Criterion** | **Description** | **Score** |
| --- | --- | --- |
| **Assessment of Outcome** | **PHN was assessed using diagnostic and prescription codes, but no external clinical validation was reported.** | **0** |
| **Follow-Up Adequacy** | **Patients were followed up for ≥365 days, sufficient to capture PHN development.** | **1** |
| **Completeness of Follow-Up** | **No significant loss to follow-up was reported.** | **1** |

**Total NOS Score**

- **Selection: 3/4**
- **Comparability: 2/2**
- **Outcome: 2/3**
- **Total Score: 7/9**

**Quality Rating**

- **Moderate-High Quality Study (Total Score: 7/9)**

**1-29. Basic Information**

- **Title**: Relationships of varicella zoster virus (VZV)-specific cell-mediated immunity and persistence of VZV DNA in saliva and the development of postherpetic neuralgia in patients with herpes zoster.
- **Authors**: Seong Yeon Park, et al
- **Country**: Republic of Korea
- **Published Year**: 2019
- **Journal**: Journal of Medical Virology
- **Study Design**: Prospective Cohort Study

**2. OS Scoring**

**1. Selection (2/4)**

| **Criterion** | **Description** | **Score** |
| --- | --- | --- |
| **Representativeness of the Cohort** | **Patients were recruited from two hospitals; the sample is small and not representative of the general population.** | **0** |
| **Selection of the Non-Exposed Cohort** | **Non-PHN patients were appropriately included as a comparison group within the cohort.** | **1** |
| **Ascertainment of Exposure** | **Exposure factors, including VZV-specific CMI and salivary VZV DNA, were assessed using validated laboratory methods.** | **1** |
| **Outcome Was Not Present at Start** | **PHN was assessed ≥90 days after rash onset, ensuring it was not present at baseline.** | **1** |

**2. Comparability (1/2)**

| **Criterion** | **Description** | **Score** |
| --- | --- | --- |
| **Comparability of Cohorts** | **Adjusted for age and immunocompromised status, which are key confounders.** | **1** |
| **Control for Additional Factors** | **Did not adjust for other potentially important confounders, such as severity of rash or acute pain.** | **0** |

**3. Outcome (2/3)**

| **Criterion** | **Description** | **Score** |
| --- | --- | --- |
| **Assessment of Outcome** | **PHN was assessed using patient-reported outcomes and clinical evaluations, but no external validation or blinding was performed.** | **0** |
| **Follow-Up Adequacy** | **Follow-up was sufficient to assess PHN and other outcomes.** | **1** |
| **Completeness of Follow-Up** | **Minimal loss to follow-up was reported; all participants were included in the final analysis.** | **1** |

**Corrected Total NOS Score**

- **Selection: 2/4**
- **Comparability: 1/2**
- **Outcome: 2/3**
- **Total Score: 5/9**

**Corrected Quality Rating**

- **Moderate-Quality Study (Total Score: 5/9)**

**1-30. Basic Information**

- **Title**: Sleep Shortage Is Associated With Postherpetic Neuralgia Development Through Hyperesthesia and Acute Pain Intensity: A Community-Based Prospective Cohort Study.
- **Authors**: Keiko Yamada, et al.
- **Country**: Japan
- **Published Year**: 2019
- **Journal**: Pain Practice
- **Study Design**: Prospective Cohort Study

**2. NOS Scoring**

**1. Selection (2/4)**

| **Criterion** | **Description** | **Score** |
| --- | --- | --- |
| **Representativeness of the Cohort** | **Large community-based cohort with wide age range; however, the sample is limited to a specific region in Japan, reducing generalizability.** | **0** |
| **Selection of the Non-Exposed Cohort** | **Non-PHN patients were included as a comparison group within the cohort.** | **1** |
| **Ascertainment of Exposure** | **Sleep duration was self-reported, introducing recall and misclassification bias.** | **0** |
| **Outcome Was Not Present at Start** | **PHN was not present at baseline, as outcomes were tracked prospectively.** | **1** |

**2. Comparability (2/2)**

| **Criterion** | **Description** | **Score** |
| --- | --- | --- |
| **Comparability of Cohorts** | **Adjusted for key confounders, including age, depressive tendencies, and alcohol consumption.** | **1** |
| **Control for Additional Factors** | **Additional adjustments were made for neoplasm treatment and BMI.** | **1** |

**3. Outcome (2/3)**

| **Criterion** | **Description** | **Score** |
| --- | --- | --- |
| **Assessment of Outcome** | **PHN was assessed using validated clinical tools and follow-up questionnaires, but no external validation or blinding was performed.** | **0** |
| **Follow-Up Adequacy** | **Follow-up was sufficient (3 years) to capture the development of PHN.** | **1** |
| **Completeness of Follow-Up** | **Minimal loss to follow-up was reported, with comprehensive tracking of outcomes.** | **1** |

**Corrected Total NOS Score**

- **Selection: 2/4**
- **Comparability: 2/2**
- **Outcome: 2/3**
- **Total Score: 6/9**

**Corrected Quality Rating**

- **Moderate-High Quality Study (Total Score: 6/9)**

**1-31. Basic Information**

- **Title**: The risk of postherpetic neuralgia in COVID-19 vaccination-associated herpes zaoster: A retrospective cohort study using TriNetX.
- **Authors**: Sheng-Hsiang Ma, et al
- **Country**: China，Taiwan
- **Published Year**: 2024
- **Journal**: Vaccine
- **Study Design**: Retrospective Cohort Study

**2**. **Newcastle-Ottawa Scale (NOS) Quality Assessment**

**1. Selection (3/4)**

| **Criterion** | **Description** | **Score** |
| --- | --- | --- |
| **Representativeness of the Cohort** | Participants were sourced from the TriNetX database, covering a large and diverse US population. | **1** |
| **Selection of the Non-Exposed Cohort** | Non-COVID-19 vaccination-associated HZ patients were used as controls. | **1** |
| **Ascertainment of Exposure** | COVID-19 vaccination status was recorded using reliable procedural codes. | **1** |
| **Outcome Was Not Present at Start** | Patients with PHN were identified after ≥3 months following HZ onset, ensuring no baseline presence. | **0** |

**2. Comparability (2/2)**

| **Criterion** | **Description** | **Score** |
| --- | --- | --- |
| **Comparability of Cohorts** | Propensity score matching adjusted for age, sex, and comorbidities. | **1** |
| **Control for Additional Factors** | Additional adjustments for antiviral use, malignancy, and other variables were included. | **1** |

**3. Outcome (3/3)**

| **Criterion** | **Description** | **Score** |
| --- | --- | --- |
| **Assessment of Outcome** | PHN was assessed using validated ICD-10 codes and follow-up records. | **1** |
| **Follow-Up Adequacy** | Follow-up extended up to 2 years, sufficient for assessing PHN development. | **1** |
| **Completeness of Follow-Up** | Comprehensive follow-up with minimal missing data or withdrawals. | **1** |

**Corrected Total NOS Score**

- **Selection**: **3/4**
- **Comparability**: **2/2**
- **Outcome**: **3/3**
- **Total Score**: **8/9**

**Quality Rating**

- **High-Quality Study** (Total Score: 8/9)

**1-32. Basic Information**

- **Title**: Use of S-LANSS, a Tool for Screening Neuropathic Pain, for Predicting Postherpetic Neuralgia in Patients After Acute Herpes Zoster Events: A Single-Center, 12-Month, Prospective Cohort Study.
- **Authors**: Soo Ick Cho, et al
- **Country**: South Korea
- **Published Year**: 2014
- **Journal**: The Journal of Pain
- **Study Design**: Prospective Cohort Study

2. **NOS Scoring**

**1. Selection (2/4)**

| **Criterion** | **Description** | **Score** |
| --- | --- | --- |
| **Representativeness of the Cohort** | Patients were recruited from a single dermatology hospital, limiting generalizability. | **0** |
| **Selection of the Non-Exposed Cohort** | Non-PHN patients were appropriately included as a comparison group within the cohort. | **1** |
| **Ascertainment of Exposure** | S-LANSS and VAS were validated tools for assessing neuropathic pain and pain intensity. | **1** |
| **Outcome Was Not Present at Start** | PHN was prospectively assessed, ensuring it was not present at baseline. | **1** |

**2. Comparability (2/2)**

| **Criterion** | **Description** | **Score** |
| --- | --- | --- |
| **Comparability of Cohorts** | Adjusted for confounders like age, severity of pain, and severity of rash. | **1** |
| **Control for Additional Factors** | Included key factors like S-LANSS scores and comorbidities in statistical analyses. | **1** |

**3. Outcome (2/3)**

| **Criterion** | **Description** | **Score** |
| --- | --- | --- |
| **Assessment of Outcome** | PHN was assessed through clinical follow-ups and validated scales (VAS), but no external validation or blinding was conducted. | **0** |
| **Follow-Up Adequacy** | Follow-up of 12 months was sufficient to assess the development of PHN. | **1** |
| **Completeness of Follow-Up** | Minimal loss to follow-up was reported; the final analysis included 305 participants. | **1** |

**Corrected Total NOS Score**

- **Selection**: **2/4**
- **Comparability**: **2/2**
- **Outcome**: **2/3**
- **Total Score**: **6/9**

**Corrected Quality Rating**

- **Moderate-High Quality Study** (Total Score: 6/9)

**1-33. Basic Information**

- **Title**: Study of Risk Factors for Postherpetic Neuralgia
- **Authors**: Hu Jian, et all
- **Country**: China
- **Published Year**: 2022
- **Journal**: National Medical Journal of China
- **Study Design**: Cross-sectional Study

**2. Newcastle-Ottawa Scale (NOS) Quality Assessment**

**1. Selection (2/4)**

| **Criterion** | **Description** | **Score** |
| --- | --- | --- |
| **Representativeness of the Cohort** | **Patients recruited from a single hospital; the study may lack generalizability to other populations.** | **0** |
| **Selection of the Non-Exposed Cohort** | **Non-PHN patients (control group) were appropriately included.** | **1** |
| **Ascertainment of Exposure** | **Exposure (e.g., hypertension, rash location) determined from medical records and interviews.** | **1** |
| **Outcome Was Not Present at Start** | **PHN was defined post-rash resolution, ensuring it was not present at baseline.** | **1** |

**2. Comparability (1/2)**

| **Criterion** | **Description** | **Score** |
| --- | --- | --- |
| **Comparability of Cohorts** | **Adjusted for key confounders like age and immunosuppressive treatments.** | **1** |
| **Control for Additional Factors** | **No additional control for other comorbidities or medications affecting immune function.** | **0** |

**3. Outcome (2/3)**

| **Criterion** | **Description** | **Score** |
| --- | --- | --- |
| **Assessment of Outcome** | **PHN outcome based on consensus diagnostic criteria; no external validation or blinding.** | **0** |
| **Follow-Up Adequacy** | **Duration of follow-up sufficient to assess PHN (≥1 month).** | **1** |
| **Completeness of Follow-Up** | **No significant loss to follow-up reported.** | **1** |

**Corrected Total NOS Score**

- **Selection: 2/4**
- **Comparability: 1/2**
- **Outcome: 2/3**
- **Total Score: 5/9**

**Quality Rating**

- **Moderate Quality Study (Total Score: 5/9)**

**1-34. Basic Information**

- **Title**: A systematic review and meta-analysis of risk factors for postherpetic neuralgia
- **Authors**: Forbes HJ, et al
- **Country**: United Kingdom,
- **Published Year**: 2016
- **Journal**: Pain

| **AMSTAR-2 Domain** | **MEET** | **Comment** |
| --- | --- | --- |
| 1. PICO components clearly defined | Yes | Population, exposures, outcomes all specified |
| 2. Protocol registered before commencement (critical) | No | No protocol or PROSPERO record reported |
| 3. Explanation for inclusion of study designs | Yes | Prospective cohorts and one case–base clearly justified |
| 4. Comprehensive literature search (critical) | Yes | MEDLINE + Embase + grey lit only; |
| 5. Study selection in duplicate | Yes | Two authors independently screened |
| 6. Data extraction in duplicate | Yes | Two authors independently extracted and cross-checked |
| 7. List of excluded studies and justification | Yes | Excluded studies tabulated in supplement |
| 8. Description of included studies | Yes | Characteristics table provided |
| 9. Risk of bias assessment of individual studies (critical) | Yes | Cochrane-style domains assessed for each cohort |
| 10. Report sources of funding for included studies | No | Individual study funding not consistently reported |
| 11. Appropriate meta-analytic methods (critical) | Yes | Fixed/random effects chosen by I²; heterogeneity, sensitivity, subgroup, metaregression |
| 12. Impact of risk of bias on results | Yes | Sensitivity analyses excluding high-risk studies |
| 13. Explanation of heterogeneity | Yes | I², Q and subgroup analyses reported |
| 14. Investigation of publication bias (critical) | Yes | Funnel plot for gender; Egger’s test reported |
| 15. Report potential sources of conflict of interest | Yes | Authors’ conflicts and funding statements included |
| 16. Account for risk of bias when interpreting/discussing results | Yes | Discussion acknowledges study limitations and bias |

**Total items met: 14/16**

**Overall rating: Critically low confidence (≥2 critical flaws)**

**AMSTAR-2 score: 14/16**

**1-35. Basic Information**

- **Title**: A systematic review and meta-analysis of independent risk factors for postherpetic neuralgia
- **Authors**: Zhou H. et al.
- **Country**: China
- **Published Year**: 2021
- **Journal**: Annals of Palliative Medicine

| **Item** | **Domain** | **Meet** | **Comment** |
| --- | --- | --- | --- |
| 1 | Did the research questions and inclusion criteria include PICO components? | Yes | Population, risk factors, comparator and PHN outcome clearly specified. |
| 2 | Was a protocol registered before the review commenced? | No | No PROSPERO or other protocol registration was reported. |
| 3 | Did the authors justify their selection of study designs for inclusion? | Yes | Inclusion of cohort studies was clearly explained. |
| 4 | Did the authors use a comprehensive literature search strategy? | No | Only PubMed was searched; no additional databases, grey literature, or non–English sources. |
| 5 | Was study selection performed in duplicate? | Yes | Two reviewers independently screened records, with disagreements resolved by a third. |
| 6 | Was data extraction performed in duplicate? | Yes | Two reviewers independently extracted data, with consensus procedures for discrepancies. |
| 7 | Did the authors provide a list of excluded studies and justify exclusions? | No | Only a PRISMA flow diagram was provided; no table or appendix listing excluded studies with reasons. |
| 8 | Did the authors describe included studies in adequate detail? | Yes | Table 1 presents study characteristics, PHN definitions, and risk-factor methods for all 14 cohorts. |
| 9 | Did the authors use a satisfactory technique for assessing risk of bias in individual studies? | Yes | NOS was applied to each cohort study, with scores reported and discrepancies resolved by a third reviewer. |
| 10 | Did the authors report on the sources of funding for the included studies? | Yes | Most original studies’ funding sources are listed in the characteristics table. |
| 11 | If meta-analysis was performed, did the authors use appropriate methods for statistical combination of results? | Yes | Fixed or random effects models chosen based on I²; heterogeneity tests, sensitivity, subgroup, and meta-regression analyses conducted. |
| 12 | Did the authors assess the potential impact of risk of bias in individual studies on the review results? | Yes | Sensitivity analyses excluding high-bias studies demonstrated result stability. |
| 13 | Did the authors account for risk of bias when interpreting/discussing the review results? | Yes | The discussion section explicitly addresses study limitations and their likely impact on conclusions. |
| 14 | Did the authors provide a satisfactory explanation for any heterogeneity observed? | Yes | Sources of heterogeneity (age, pain severity, study design) were explored via subgroup and meta-regression analyses. |
| 15 | If quantitative synthesis was performed, did the authors investigate publication bias and discuss its likely impact? | Yes | A funnel plot was shown and Egger’s test reported (p=0.054), indicating minimal small-study effects. |
| 16 | Did the authors report any potential sources of conflict of interest, including funding they received for the review? | Yes | A conflict-of-interest statement is included, and review funding is declared. |

**Failed critical domains: 2 (protocol), 4 (search), 7 (excluded-studies list)**

**Total items met: 13/16**

**Overall rating: Critically low confidence (≥2 critical flaws)**

**1-36. Basic Information**

- **Title**: Association of the incidence of postherpetic neuralgia with early treatment intervention of herpes zoster and patient baseline characteristics: A systematic review and meta analysis of cohort studies
- **Authors**: Ding S. et al
- **Country**: China
- **Published Year**: 2024
- **Journal**: Int. J. Infect. Dis.

| **Item** | **Domain** | **Meet** | **Comment** |
| --- | --- | --- | --- |
| 1 | Did the research questions and inclusion criteria include PICO components? | Yes | Population, exposures, comparators and PHN outcome all clearly defined. |
| 2 | Was a protocol registered before the review commenced? | Yes | PROSPERO registration CRD42023486252 reported. |
| 3 | Did the authors justify their selection of study designs for inclusion? | Yes | Inclusion of prospective and retrospective cohorts clearly explained. |
| 4 | Did the authors use a comprehensive literature search strategy? | Yes | Searched seven databases (English + Chinese) with no language restrictions. |
| 5 | Was study selection performed in duplicate? | Yes | Two investigators independently screened, discrepancies resolved by a third. |
| 6 | Was data extraction performed in duplicate? | Yes | Two investigators independently extracted data with consensus procedures. |
| 7 | Did the authors provide a list of excluded studies and justify exclusions? | Yes | Only a PRISMA flowchart is shown; |
| 8 | Did the authors describe included studies in adequate detail? | Yes | Supplementary Table 1 lists key characteristics for all 53 cohorts. |
| 9 | Did the authors use a satisfactory technique for assessing risk of bias in individual studies? | Yes | NOS was applied to each cohort and scores reported. |
| 10 | Did the authors report on the sources of funding for the included studies? | No | Funding sources of the original cohort studies were not systematically reported. |
| 11 | If meta-analysis was performed, did the authors use appropriate methods for statistical combination of results? | Yes | Random‐effects model chosen; heterogeneity (I²), sensitivity, subgroup and meta‐regression. |
| 12 | Did the authors assess the potential impact of risk of bias in individual studies on the review results? | Yes | Sensitivity analyses excluding high‐bias studies demonstrated robustness. |
| 13 | Did the authors provide a satisfactory explanation for any heterogeneity observed? | Yes | Heterogeneity sources explored via subgroup and meta‐regression analyses. |
| 14 | If quantitative synthesis was performed, did the authors investigate publication bias and discuss its likely impact? | Yes | Funnel plots and Egger’s test (p=0.054) were reported for key outcomes. |
| 15 | Did the authors report any potential sources of conflict of interest, including funding they received for the review? | Yes | A conflict‐of‐interest statement and review funding are declared. |
| 16 | Did the authors account for risk of bias when interpreting/discussing the review results? | Yes | Discussion explicitly addresses study limitations and bias impact. |

**Total items met: 15/16**

**Overall rating: With one critical flaw, the review is rated High confidence.**
