# Supplementary material for: Risk factors for postherpetic neuralgia: a meta-analysis based on demographic, clinical features, and treatment characteristics
Source: Front Immunol. 2025 Oct 1;16:1667364. doi: 10.3389/fimmu.2025.1667364 (PMC12521459; doi:10.3389/fimmu.2025.1667364)
Supplement: Supplementary file 4 [file Table3.docx]

Sensitivity analysis (Age)
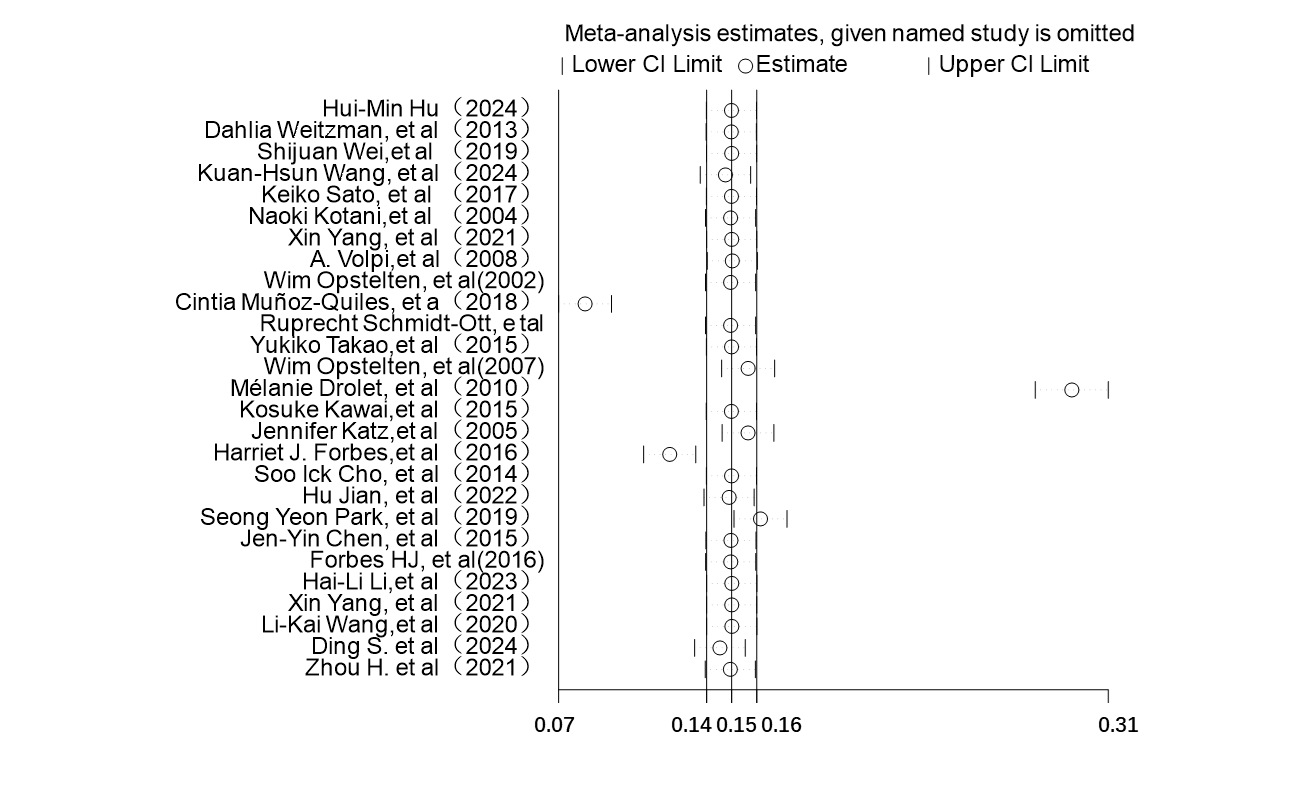


Egger’s test (Age)


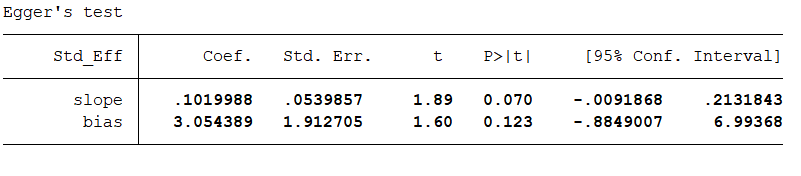


Sensitivity analysis (Gender)

Egger’s test (Gender)


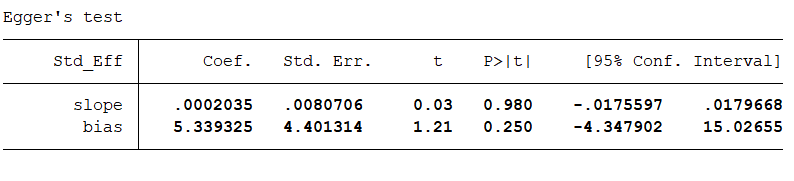


Sensitivity analysis (Social economic)


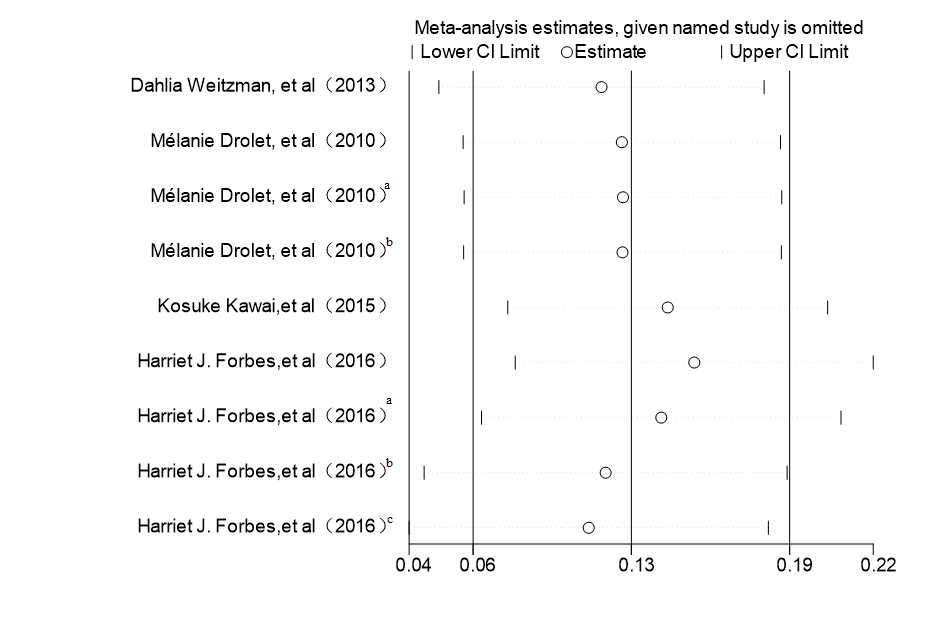


Egger’s test (Social economic)


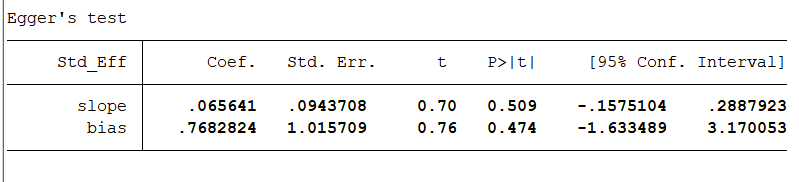


Sensitivity analysis (Life history)


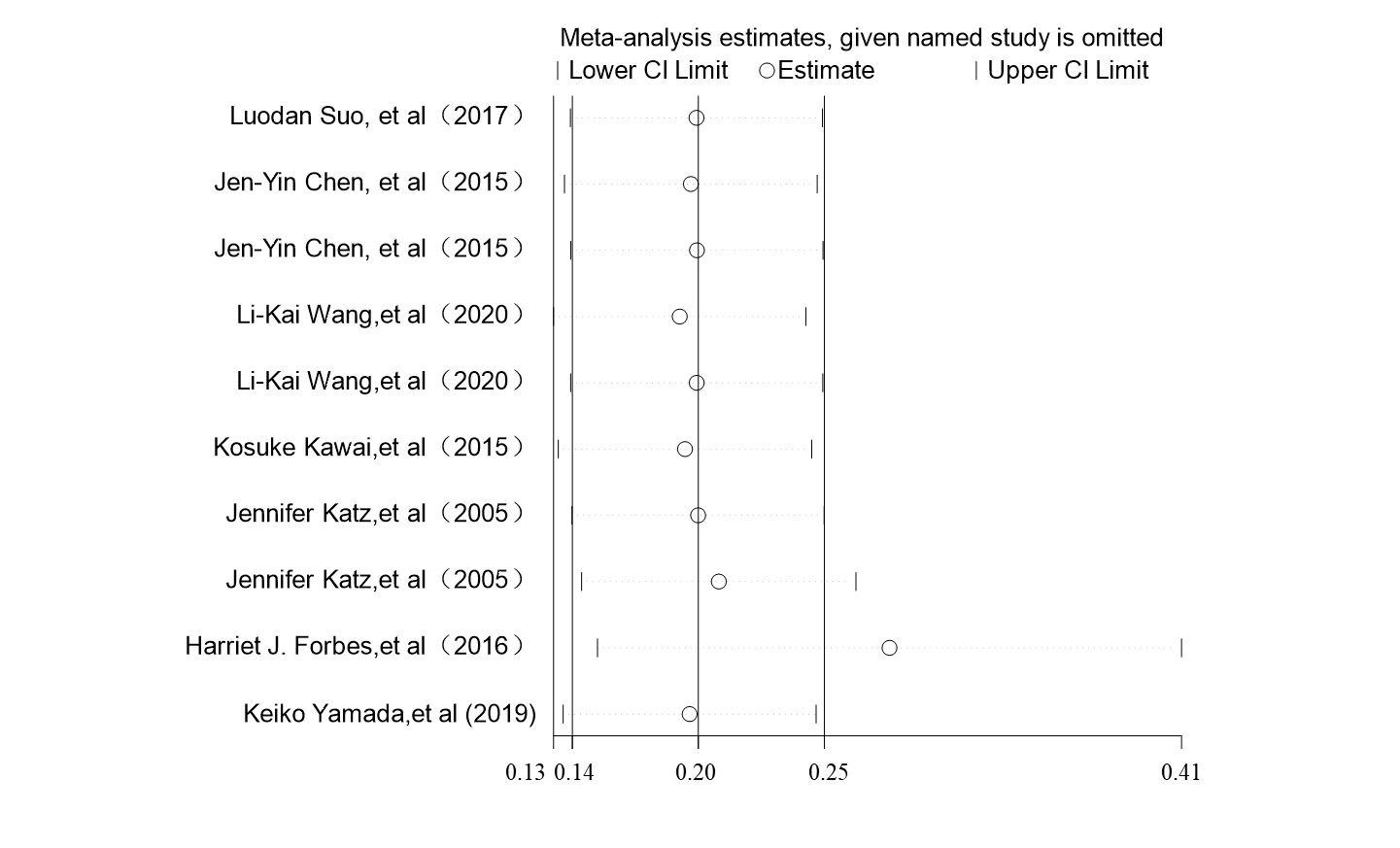


Egger’s test (Life history)


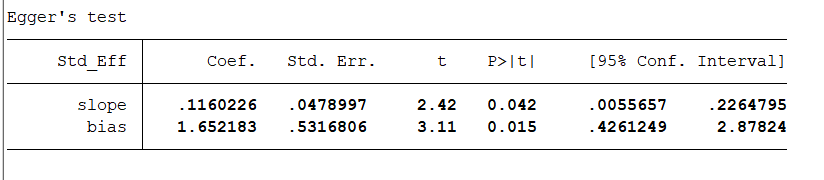


Sensitivity analysis (Clinical symptom)


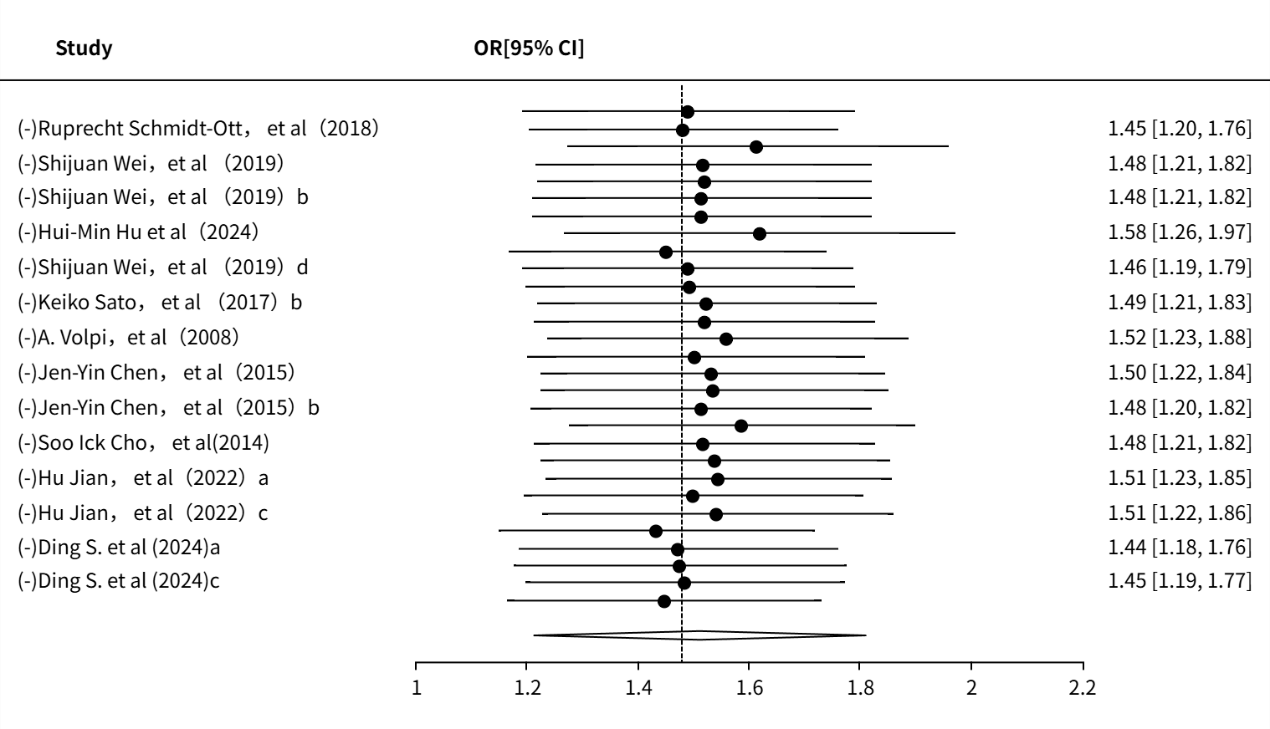


Egger’s test (Clinical symptom)


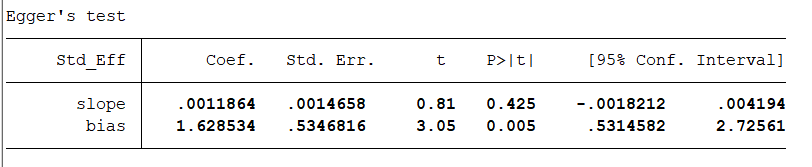


Sensitivity analysis (Pain)


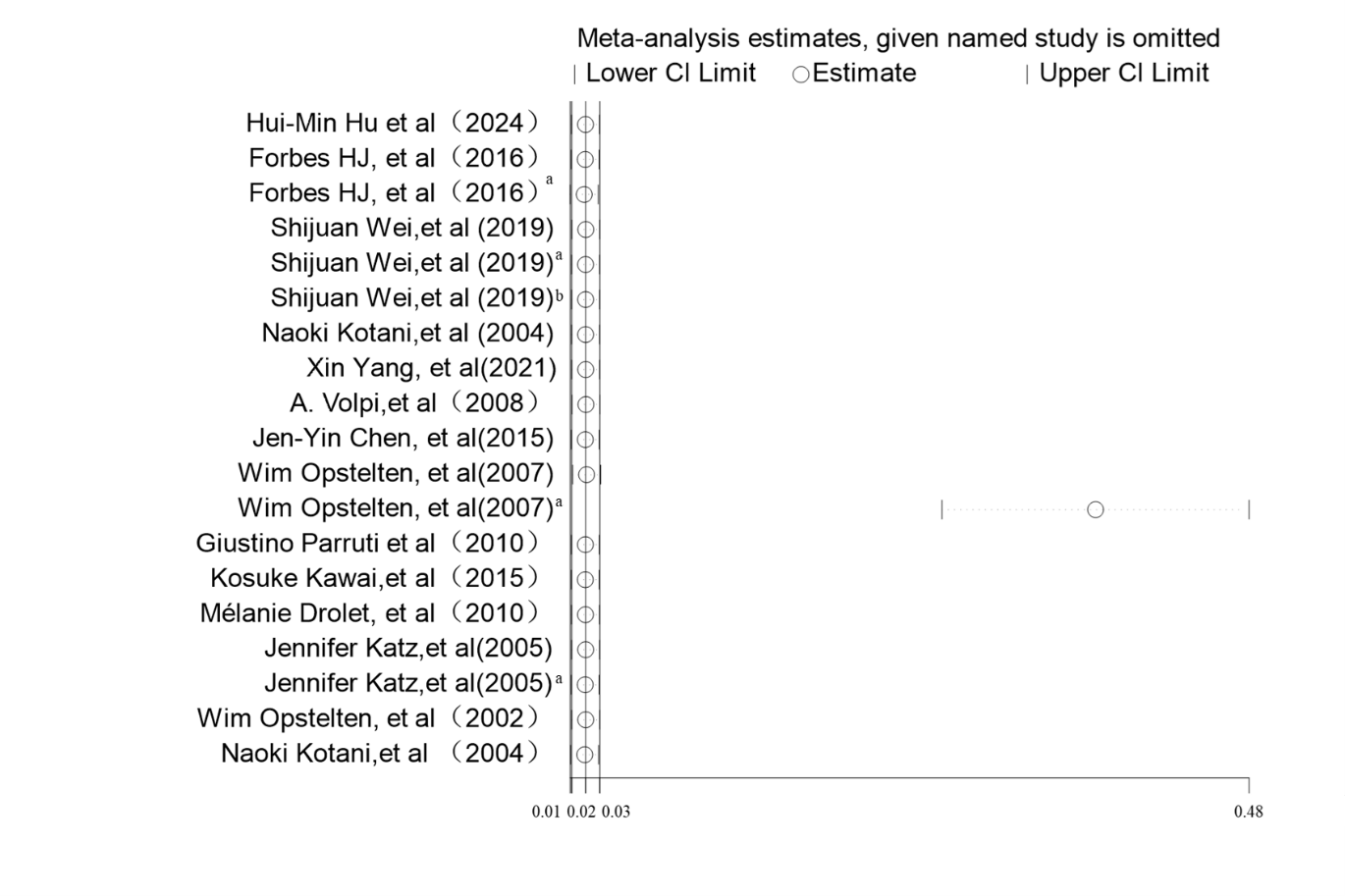


Egger’s test (Pain)


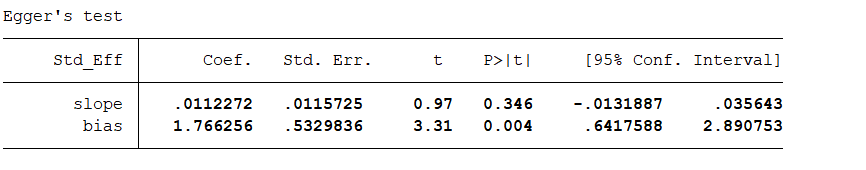


Sensitivity analysis (Clinical index)


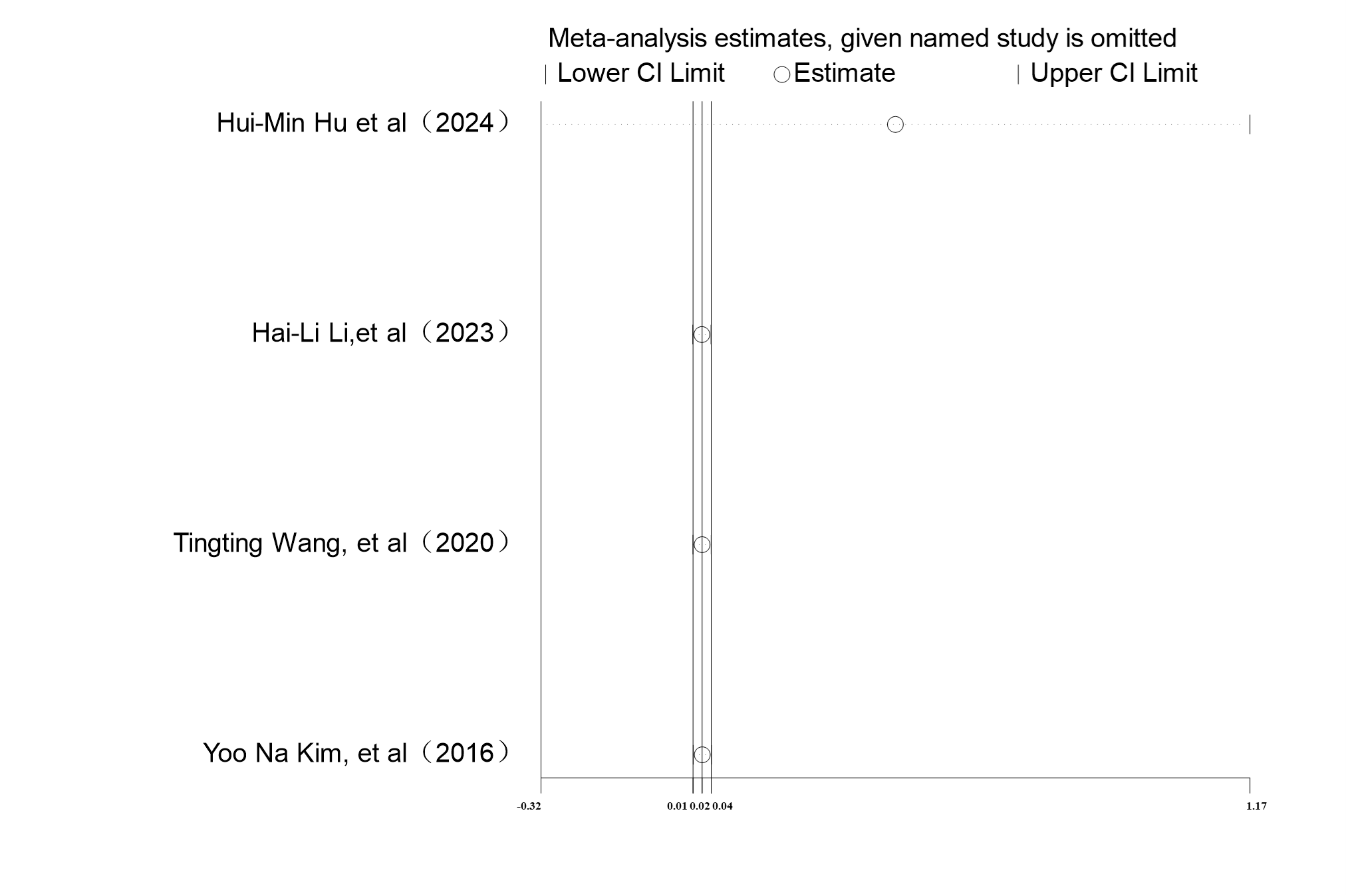


Egger’s test (Clinical index)


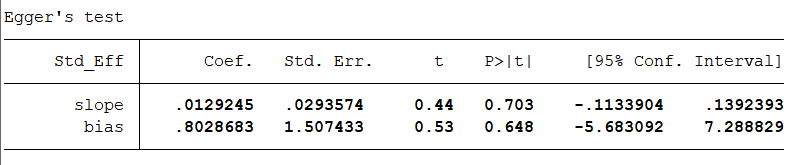


Sensitivity analysis (Therapy)


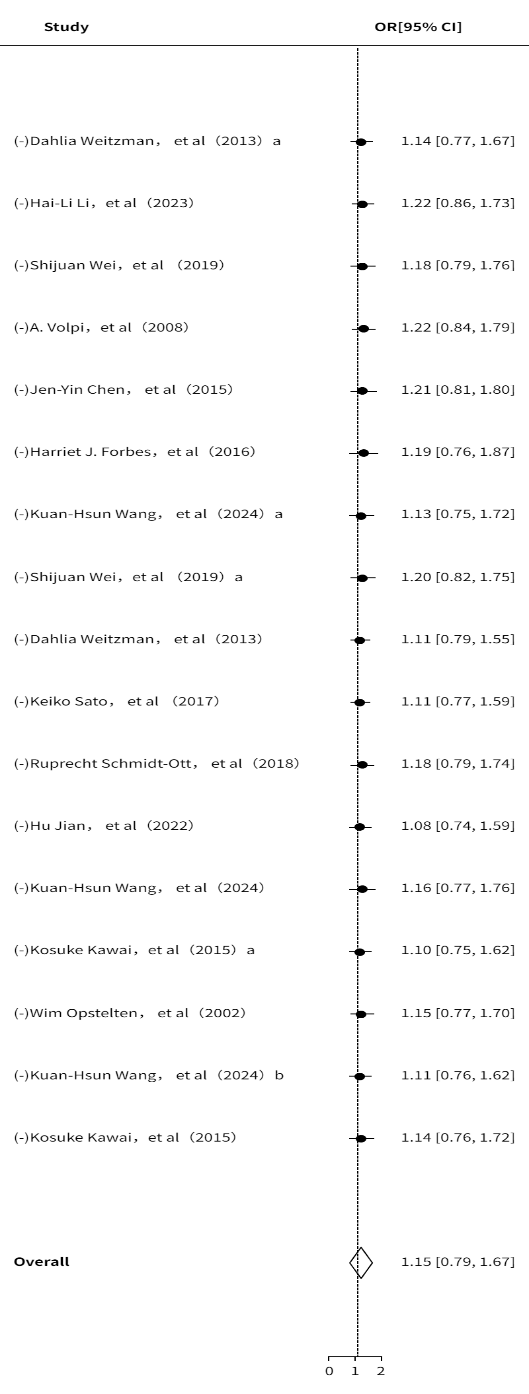


Egger’s test (Therapy)


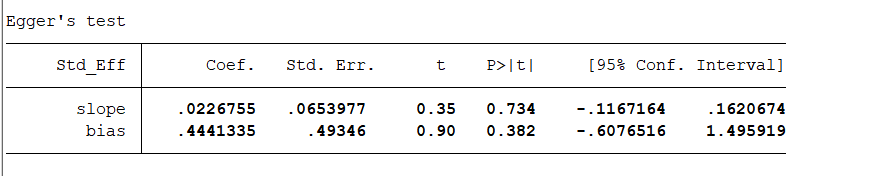


Sensitivity analysis (Virus)


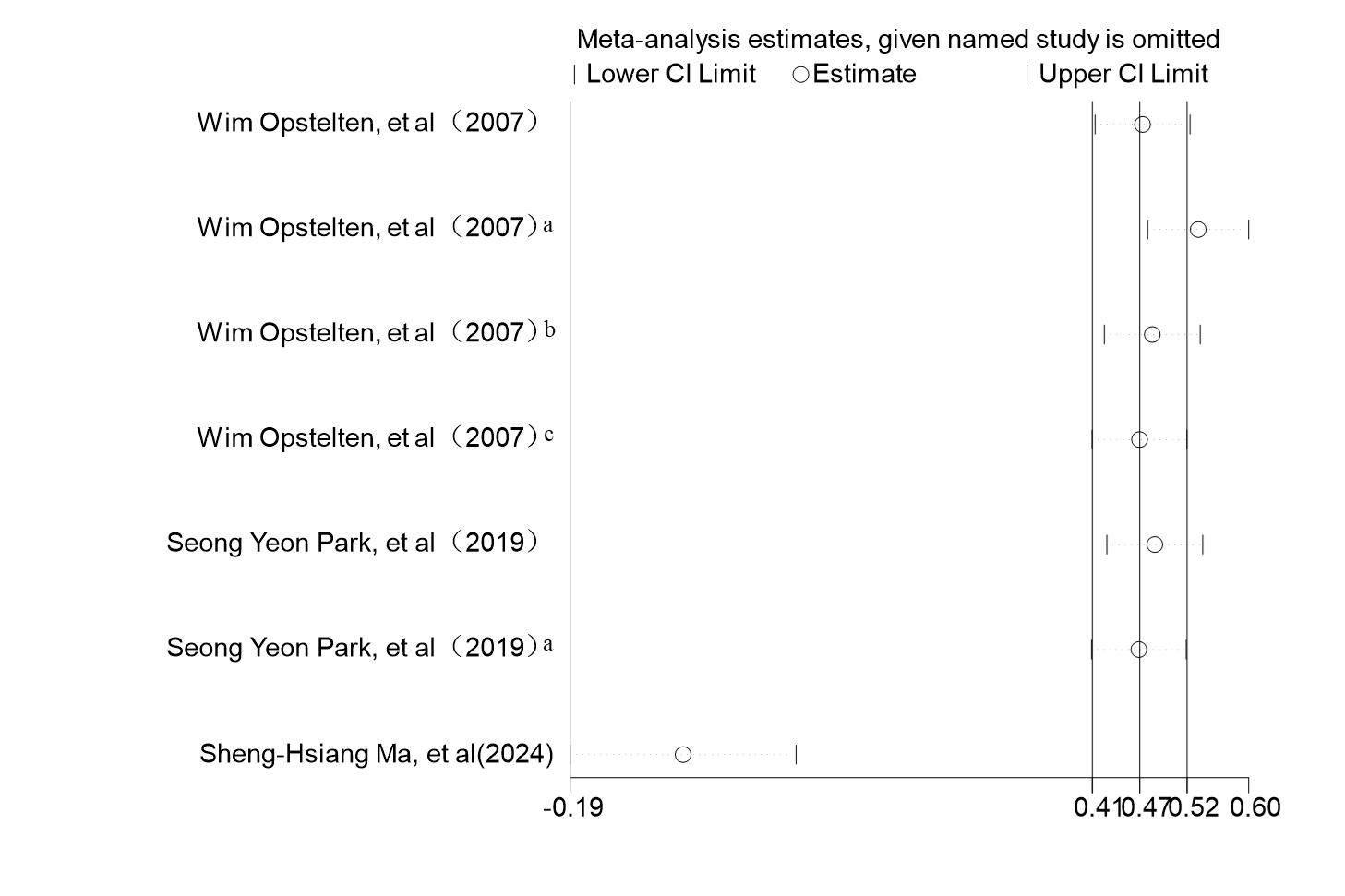


Egger’s test (Virus)


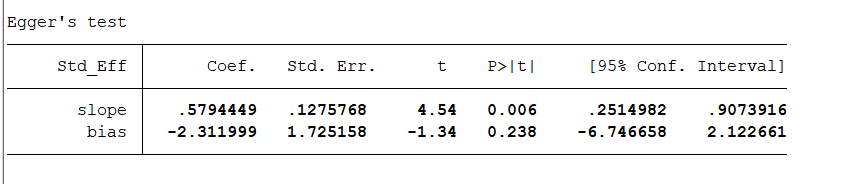


Sensitivity analysis (DM)


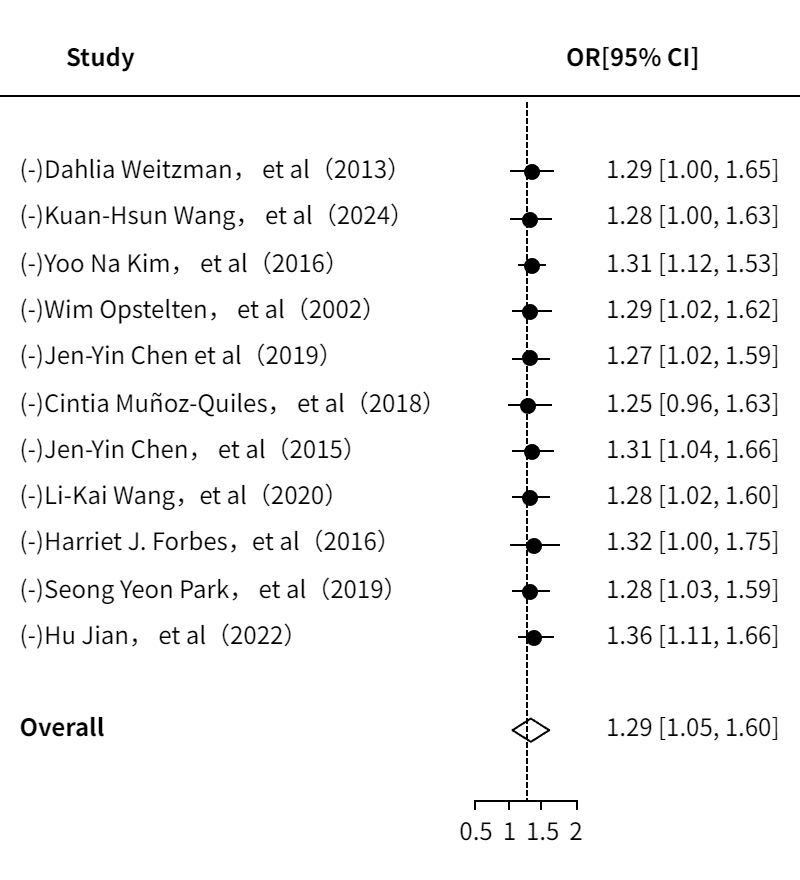


Egger’s test (DM)

| Egger test |  |  |  |  |  |
| --- | --- | --- | --- | --- | --- |
|  |  | Coef. | Std. err. | t | *p* |
|  | Slope bias | -0.640 | 0.637 | -1.004 | 0.342 |

Sensitivity analysis (COPD)


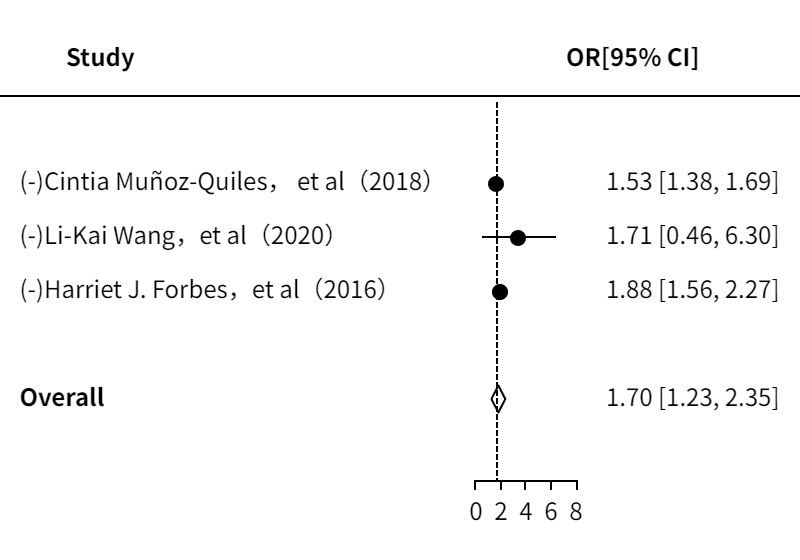


Egger’s test (COPD)

| Egger test |  |  |  |  |  |
| --- | --- | --- | --- | --- | --- |
|  |  | Coef. | Std. err. | t | *p* |
|  | Slope bias | -1.647 | 2.391 | -0.689 | 0.616 |

Egger’s test (COPD)

| Egger test |  |  |  |  |  |
| --- | --- | --- | --- | --- | --- |
|  |  | Coef. | Std. err. | t | *p* |
|  | Slope bias | -0.780 | 1.442 | -0.541 | 0.643 |
