# Supplementary material for: Risk factors for postherpetic neuralgia: a meta-analysis based on demographic, clinical features, and treatment characteristics
Source: Front Immunol. 2025 Oct 1;16:1667364. doi: 10.3389/fimmu.2025.1667364 (PMC12521459; doi:10.3389/fimmu.2025.1667364)
Supplement: Supplementary file 5 [file Table4.docx]

Search strategies：

Medicine/Pubmed：

(("Postherpetic neuralgia"[MeSH] OR PHN[Title/Abstract] OR "postherpetic neuralgia"[Title/Abstract] OR "post-herpetic neuralgia"[Title/Abstract] OR "post herpetic neuralgia"[Title/Abstract] OR "postherpetic pain"[Title/Abstract] OR "post-herpetic pain"[Title/Abstract] OR "post herpetic pain"[Title/Abstract] OR "neuralgia"[MeSH] AND "Herpes Zoster"[MeSH]) OR ("Neuralgia"[MeSH] OR "Pain"[MeSH] OR pain[Title/Abstract] AND ("Herpes Zoster"[MeSH] OR zoster[Title/Abstract] OR shingles[Title/Abstract] OR zona[Title/Abstract] OR VZV[Title/Abstract])))

AND ("Risk Factors"[MeSH] OR "Epidemiologic Studies"[MeSH] OR "Odds Ratio"[MeSH] OR "Multivariate Analysis"[MeSH] OR "Logistic Models"[MeSH] OR "Prevalence"[MeSH] OR "Incidence"[MeSH] OR "odds ratio"[Title/Abstract] OR "risk ratio"[Title/Abstract] OR "relative risk"[Title/Abstract] OR risk factor[Title/Abstract] OR predict[Title/Abstract] OR correlate[Title/Abstract] OR etiology[Title/Abstract] OR aetiology[Title/Abstract] OR incidence[Title/Abstract] OR rate[Title/Abstract])OR(("Neuralgia/etiology"[MeSH] OR "Pain/etiology"[MeSH]) AND ("Herpes Zoster"[MeSH] OR zoster [Title/Abstract] OR shingles [Title/Abstract] OR zona [Title/Abstract] OR VZV[Title/Abstract]))

AND"HUMANS"[MeSH]; limited to language: ENGLISH

Embase

(("Postherpetic neuralgia"[exploded subject heading] OR "PHN"[Title or abstract] OR "postherpetic neuralgia"[Title or abstract] OR "post-herpetic neuralgia"[Title or abstract] OR "post herpetic neuralgia"[Title or abstract] OR "postherpetic pain"[Title or abstract]OR "post-herpetic pain"[Title or abstract] OR "post herpetic pain"[Title or abstract] OR "Neuralgia"[exploded subject heading] OR "Pain"[exploded subject heading] OR "neuralgia"[Title or abstract] OR "pain"[Title or abstract]) AND ("herpes zoster"[exploded subject heading] OR "zoster"[Title or abstract] OR "shingles"[Title or abstract] OR "zona"[Title or abstract] OR "VZV"[Title or abstract]))

AND ( "Risk factor"[exploded subject heading] OR "Epidemiology"[exploded subject heading] OR "Odds ratio"[exploded subject heading] OR "Multivariate analysis"[exploded subject heading] OR "Statistical model"[exploded subject heading] OR "Prevalence"[exploded subject heading] OR "Incidence"[exploded subject heading] OR "odds ratio"[Title or abstract] OR "risk ratio"[Title or abstract] OR "relative risk"[Title or abstract] OR "risk"[Title or abstract] OR "risk factor"[Title or abstract] OR "predict"[Title or abstract] OR "correlate"[Title or abstract] OR "etiol"[Title or abstract] OR "aetiol"[Title or abstract] OR "incidence"[Title or abstract]OR "rate"[Title or abstract]) OR( ("Neuralgia/etiology"[subject heading] OR "Pain/etiology"[subject heading])AND ("herpes zoster"[exploded subject heading] OR "zoster"[Title or abstract] OR "shingles"[Title or abstract] OR "zona"[Title or abstract] OR "VZV"[Title or abstract]))

AND"HUMANS"[subject heading]; limited to language: ENGLISH
